# Photoredox catalysis harvesting multiple photon or electrochemical energies

**DOI:** 10.3762/bjoc.19.81

**Published:** 2023-07-28

**Authors:** Mattia Lepori, Simon Schmid, Joshua P Barham

**Affiliations:** 1 Fakultät für Chemie und Pharmazie, Universität Regensburg, Universitatsstraße 31, 93040 Regensburg, Germany

**Keywords:** consecutive photoinduced electron transfer, electro-activated photoredox catalysis, photoelectrochemistry, photoredox catalysis, radical ions

## Abstract

Photoredox catalysis (PRC) is a cutting-edge frontier for single electron-transfer (SET) reactions, enabling the generation of reactive intermediates for both oxidative and reductive processes via photon activation of a catalyst. Although this represents a significant step towards chemoselective and, more generally, sustainable chemistry, its efficacy is limited by the energy of visible light photons. Nowadays, excellent alternative conditions are available to overcome these limitations, harvesting two different but correlated concepts: the use of multi-photon processes such as consecutive photoinduced electron transfer (conPET) and the combination of photo- and electrochemistry in synthetic photoelectrochemistry (PEC). Herein, we review the most recent contributions to these fields in both oxidative and reductive activations of organic functional groups. New opportunities for organic chemists are captured, such as selective reactions employing super-oxidants and super-reductants to engage unactivated chemical feedstocks, and scalability up to gram scales in continuous flow. This review provides comparisons between the two techniques (multi-photon photoredox catalysis and PEC) to help the reader to fully understand their similarities, differences and potential applications and to therefore choose which method is the most appropriate for a given reaction, scale and purpose of a project.

## Review

### 1 Introduction

**Figure 1:**
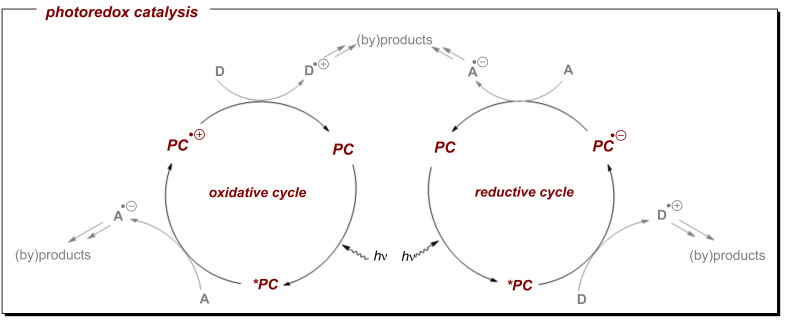
Oxidative and reductive activations of organic compounds harvesting photoredox catalysis.

Owing to the unique reactivity patterns of free radicals that often provide access to new dimensions of synthetic chemical space, the field of single electron transfer (SET) in organic synthesis has expanded considerably in the past two decades. Among this area, photoredox catalysis (PRC) is highly attractive due to its abilities i) to generate reactive intermediates under mild conditions for both oxidative and reductive reactions and ii) to use photons as traceless reagents to drive reactions in a “greener” manner [1–6]. As depicted in Figure 1, for an oxidative PRC cycle, the excited photocatalyst (*PC) firstly undergoes oxidative quenching by SET with an electron acceptor (**A**), leading to **PC^•+^** and **A^•−^**. The ground state photocatalyst is then regenerated by an SET reaction with an electron donor (**D**), affording also **D^•+^**. Both species described can be further involved in various organic transformations to form the target products (or byproducts). In a complementary manner, *PC generates **A^•−^** and **D^•+^** within a reductive quenching cycle via SET reactions. The milder conditions that PRC enjoys to access potent redox agents guarantees sustainable and safer processes when compared to classical methods of equivalent redox power. For example, in the context of deeply reductive reactions, dissolving alkali metal conditions have remained the most commonly employed both in academia and industry for over a century and even to date continue to be used despite their hazards, poor selectivity and chemical waste [7–10]. Nowadays, excellent alternative conditions are available via PRC (vide infra).

However, even if PRC provides elegant methods to circumvent these issues, it comes with its own set of limitations. In particular, the accessible energy for photocatalytically-driven transformations is generally limited by the energy of a single visible light photon (400–700 nm; 1.8–3.1 eV). In addition, this energy is also diminished by as much as ≈25% through vibrational relaxation, internal conversion and intersystem crossing [11] and hence, many highly stabilized molecules including important feedstock molecules such as arenes, haloarenes or olefins remain inert to direct photoredox activation powered by visible light [12]. Irradiation with UV photons that intrinsically possess higher energy, however, is generally unfavorable due to the high expense and thermal footprint of the reactors. Although most organic molecules directly absorb photons in the UV region, side reactions and selectivity issues arise upon direct excitation of organic molecules. In recent years, two conceptually distinct but mechanistically related strategies have emerged that enable access to excited state catalysts wielding i) higher redox power than standard monophotonic photoredox catalysts and ii) energy that parallels the energy of UV-driven transformations, but under cheaper, safer conditions and in a more selective manner by indirect substrate activation via a catalyst. These are: a) multi-photon processes that accumulate visible light photon energies for electron transfer processes and b) photoelectrochemistry (PEC) in which electronic and photonic energies are either compiled or productively utilized. This Review summarizes key examples of both strategies, presents their respective advantages and drawbacks and aims to draw comparisons that can help readers decide which strategy is a more suitable fit for a given purpose. In order to do so, the scope of our Review is thus restricted to *electron transfer* redox processes and does not include energy transfer or atom/group transfer processes. Particularly interesting are instances where the *same active catalytic intermediate is proposed* in conPET and PEC reactions (e.g., a photoexcited radical anion), yet *different reactivity outcomes arise*; the underlying reasons for such are discussed. Finally, we provide our perspective on current challenges and target areas for future exploration.

#### 1.1 Multi-photon processes

**Figure 2:**
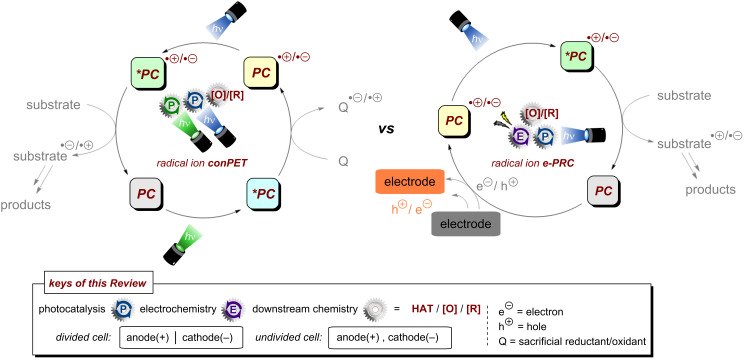
General catalytic cycles of radical ion conPET (left) and radical ion e-PRC (right).

As mentioned, the energy accessible for a PRC reaction relying on a single visible photon is limited and does not suffice for many desirable target organic substrates. Direct cumulative absorption of visible light photons by a given molecule is extremely challenging, since the short lifetimes of excited states generally do not allow their accumulation in appreciable concentrations to absorb a subsequent photon and be further photoexcited. In biological photosynthesis – nature’s omnipresent example of light-driven reactions – this limitation is overcome by transferring the energy of an initial photoexcitation process at photosystem II (**PSII**) via an electron transfer chain to photosystem I (**PSI**) where a second photoexcitation occurs [13–14]. Mimicry of this “Z-scheme” led to a seminal disclosure the concept of consecutive photoinduced electron transfer (conPET) by König and co-workers in 2014 for the generation of super-reductants [15] and by Wagenknecht in 2018 for the generation of super-oxidants [16]. Herein, initial excitation of the photocatalyst by a single photon is followed by reduction or oxidation by a sacrificial SET donor (e.g., Et_3_N [15]) or acceptor (e.g., SF_6_ [16]) to yield the catalyst radical anion or radical cation. As a semi-stable, higher energy ground-state entity, this can accumulate in sufficient concentration under the reaction conditions to absorb another photon and thereby generate a super-reducing or super-oxidizing excited state (Figure 2 left). In addition to ‘radical ion’ conPET, this Review will also cover deviating variants such as neutral (acridine) radical conPET as well as polysulfide or ‘tandem’ photoredox catalysis that similarly rely on the absorption of two photons to access activated catalyst states that engage redox-inert substrates.

Other two-photon processes where the photoredox-active species is generated by an initial energy transfer process – such as triplet-triplet annihilation (TTA) upconversion – are excluded from this Review as i) they are comprehensively and elegantly reviewed elsewhere [17], and ii) comparisons are not straightforward to make with PEC, a main theme of this Review. Protocols for sensitization-initiated electron transfer (SenI-ET) relying on a dual catalytic system of transition-metal based photocatalysts and pyrenes to generate highly reductive species are also excluded as such reported transformations are now equally achievable by a single catalyst entity [18–21].

#### 1.2 Photoelectrochemistry (PEC)

Another important vehicle for SET is synthetic organic electrochemistry (SOE) [22–23]. While undoubtedly powerful, electrochemistry can suffer limitations in reaction selectivity because the constant application of high magnitude potentials can lead to uncontrolled reactions due to the accumulation of reactive intermediates within proximity of the electrode surface. Compared to homogeneous photocatalytic processes that lend themselves to high selectivity for taming radical intermediates by taking place in bulk solution, direct electrolytic reactions taking place at the heterogeneous interface presents an additional layer of complexity to mechanistic understanding and conferring selectivity. Nonetheless, SOE has enjoyed a dramatic rise in popularity in the last decade [24–27], partly driven by reactor standardization but also thanks to developments in technology (flow, alternating polarity) and understanding that fundamentally improve selectivity. Among these is its innovative merger with PRC (synthetic PEC) in a fashion that tackles the issues of both parent techniques and has risen to the forefront of methods for SET chemistry.

In the context of synthetic molecular photoelectrochemistry, there are various sub-fields classified depending on how the electrochemical and photochemical steps interplay in the mechanism. This Review’s main focus is on electrochemically mediated photoredox catalysis (e-PRC), where the electrochemical and photochemical steps are intimately involved within the same catalytic cycle, as subsequent steps. This broadly separates into two subcategories, “radical ion e-PRC” (Figure 2, right) and “recycling e-PRC”. Radical ion e-PRC typically implicates electrogenerated radical ion doublet states which are photoexcited to yield super-oxidants or super-reductants while recycling e-PRC involves the turnover of a ‘standard’ (typically closed-shell) photoredox catalyst (**PC**) by means of anodic oxidation or cathodic reduction [28–29]. Furthermore, a series of new protocols using decoupled photoelectrochemistry (*d*PEC), where electrochemical and photochemical components have separate, discrete roles will be presented. This review excludes interfacial photoelectrochemistry (*i*PEC) processes, where reactions occur at photoelectrode surfaces. These are reviewed exhaustively elsewhere [28].

**Figure 3:**
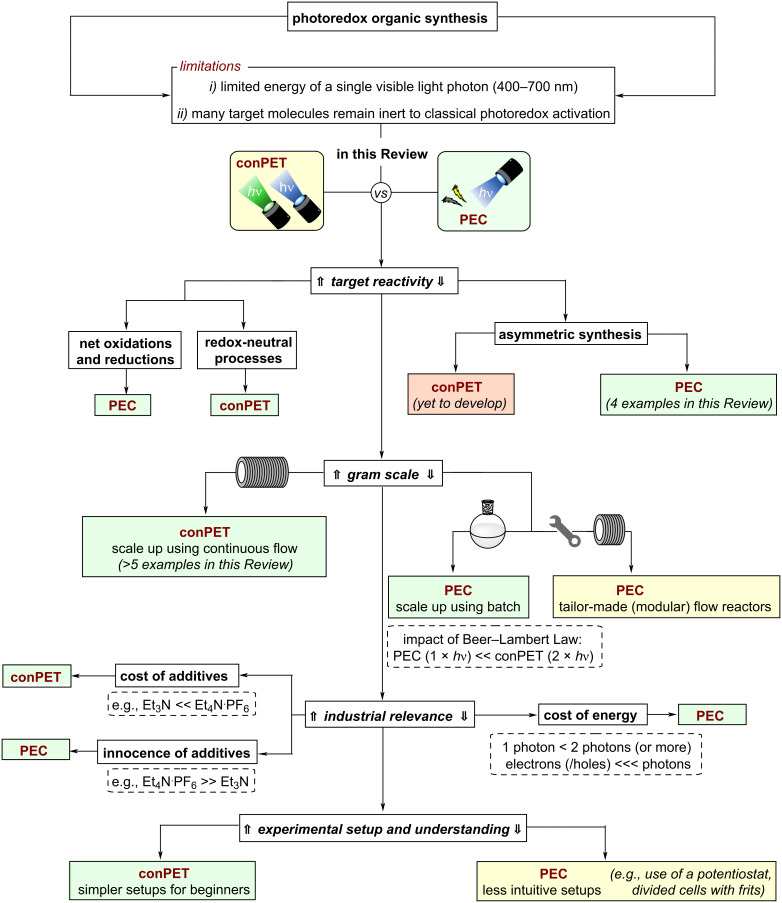
“Beginner’s guide”: comparison between advantages, capacities, and prospectives of conPET and PEC.

In recent years, both multi-photon processes and PEC developed from conceptually interesting techniques into widely applicable and well-developed methods capable of efficiently mediating and enabling difficult chemical transformations. Following a temporal order of discoveries, the reader will find the first section dedicated to reductive substrate activations via conPET and other multi-photon processes followed by more recently developed protocols for oxidative conPET. Hereafter, to provide continuity and highlight differences and comparisons between the two techniques, the Review will then focus attention on oxidative substrate activations in PEC before reductive activation examples of this field. Before plunging into the details of the two techniques, Figure 3 offers some advice for newcomers, a “Beginner’s guide” flowchart, to understand which of the two techniques might be more appropriate in certain contexts of application. Broadly speaking, it can be concluded that carrying out reactions with conPET, both in academia and industry, has a lower barrier of accessibility due to more intuitive/standardized reactor setups. Essentially, the reactor setup can be identical to standard photoredox catalysis reaction setups, although it should be noted that since there are two photoactive species with often different absorption bands or different extinction coefficients at any given wavelength, it is necessary to use i) polychromatic wavelength which is less well-defined or ii) dual(/multi) wavelength LEDs that complicate the setup. The simpler reaction setups and lack of a heterogeneous surface (electrodes) can make the mechanistic investigation more accessible. While quantum parameters (quantum yield, quadratic relationships with light intensity, etc) have been touched upon for a monochromatic light source, the impact of relative intensities of different wavelengths (λ_max,ex_ of **PC** and **PC^•^**^−/+^) has never been investigated.

Notwithstanding the above, for longer-term industrial purposes, PEC is ultimately more suitable because only one species need be photoactive, and in purely economic terms generating one photon is cheaper than two (or more). Electrons (/holes, in the form of applied potential) are cheaper than photons as well. Regarding the additives necessary for the processes, even if electrolytes needed to reduce the Ohmic drop in PEC reactions are more expensive or more abundant than sacrificial reductants/oxidants employed in conPET (e.g., tetraalkylammonium salts vs trialkylamines), it is worth emphasizing that electrolytes, generally, are chemically innocent to undesired reactions, whereas the byproducts of sacrificed amines may be involved in processes that lower the efficiency and selectivity of the reactions (vide infra*,* conPET section).

Electrolytes have the potential to be i) aqueous-separated and recovered in batch, or ii) decreased, even ultimately eliminated by flow reactors as an engineering control.

Regarding purely the chemical reactivity and scope of applications, the most marked difference between the two types of processes, however, is that conPET is more appropriate for redox-neutral reactions, whereas PEC is more appropriate for net oxidations or reductions due to the radical polar crossover nature of its reactivity [30–31]. In the former, the neutral photoexcited catalyst must be able to engage substrates/intermediates in PET to achieve a redox neutral process. In the latter, following the first photoinduced electron transfer (PET) step a subsequent electrochemical SET occurs in the same redox direction and this subsequent SET is user-tunable by the applied cell potential. These divergent reactivity features make the two techniques totally complementary, allowing the exploration of a large portion of SET-driven organic transformations using at least one of them at a time. Since ‘radical ion’ conPET/e-PRC are proposed to involve the same radical ion catalyst intermediate, the same catalyst can in principle be repurposed for either technique, and mechanistic learnings will thus be highly transferrable between the fields.

Although asymmetric transformations are yet to be achieved using conPET, the PEC section of this Review will also describe pioneering first efforts in this direction [32–33]. Finally, both techniques are amenable to large-scale synthesis and ideally integrated with state-of-the-art reactor technology platforms, such as continuous flow reactors and high throughput screening plates. Various examples of scalability will be highlighted in this Review, with a particular emphasis on the challenges and areas for improvement, such as the standardization of reactors capable to conjugate applied potential and light irradiation either in different modules or within the same flow path.

### 2 conPET in organic synthesis

#### 2.1 Reductive activation

**2.1.1 C(sp^2^)–X activation:** In the rise of visible light-mediated PRC, the generation of aryl radicals for C(sp^2^)–C(sp^2/3^) couplings under mild conditions (room temperature, visible light activation of a catalyst) was heavily investigated [34–36]. However, initially the procedures were generally limited to electron-poor arenes like diazonium/iodonium salts or aryl iodides with electron-withdrawing substituents as aryl radical precursors, due to the limited accessible reducing power of photocatalysts that relied on a monophotonic excitation event. However, the vast majority of inexpensive, commercially available aryl halides are chlorides [37–38], with potentials for reduction that almost exclusively lie beyond the threshold of monophotonically-excited photoredox catalysts (i.e., more deeply negative than *E*_1/2_ = −2.0 V vs SCE). Considering this, state-of-the-art developments have focused on the generation of super-reductants (*E_1/2_ > −2.0 V vs SCE) that accumulate the energy of multiple photons. Case studies will now be presented.

Hereafter, while we quote the excited state redox potentials from the report in question, it should be noted that these are estimates associated with uncertainties especially in the case of excited radical ions which oftentimes exhibit unusual wavelength dependencies on catalytic efficiency. Light source wavelengths/input powers (radiant flux is rarely reported) are quoted if available, readers are directed to the report in question for details. When not available, the qualitative description is used as per the report in question (e.g., ‘blue LEDs’).

**Figure 4:**
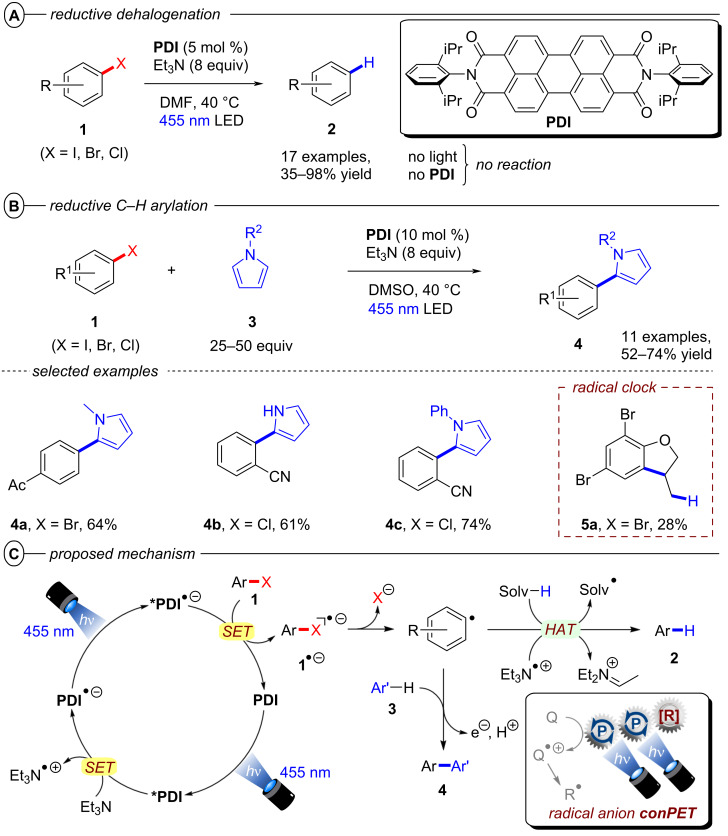
A) conPET reductive dehalogenation of aryl halides with **PDI**. B) Reductive C–H arylation with pyrroles (top) and selected examples from the substrate scope (bottom). C) Proposed mechanism.

The König group first reported a photocatalytic approach to C(sp^2^)–X activation harnessing multiple photon energies in their seminal work on perylene diimide (**PDI**) catalysts [15]. In their proposed consecutive photoinduced electron transfer (conPET) mechanism (Figure 4C), **PDI** is photoexcited and reductively quenched by Et_3_N to form its stable, colored radical anion **PDI^•−^** that can be photoexcited again to generate an even stronger reductant; *PDI^•−^ (*E_1/2_ = −1.87 V vs SCE) [34]. A SET process to the aryl halide regenerates neutral **PDI** and forms the aryl halide’s radical anion, which then undergoes C(sp^2^)–X bond fission to afford the aryl radical as a reactive intermediate. The aryl radical then either reacts via hydrogen atom transfer (HAT) with solvent molecules or Et_3_N**^•^**^+^ in an overall dehalogenation to furnish product **2**, or it is trapped with pyrrole derivatives **3** in a C–C bond formation to afford arylated products **4**. Based on the ultrashort lifetime of *PDI^•−^ (τ = 145 ps), the notion of its photochemistry has attracted skepticism and it has been suggested decomposition products of *PDI^•−^ may instead serve as reductants as a theme of ongoing debate [39]. Nonetheless, this protocol enabled the reduction of various electron-poor aryl iodides and aryl bromides and, for the first time, the reduction of aryl chlorides (albeit electron-poor ones) via visible light PRC in good to excellent yields (35–98%) (Figure 4A). Notably, the protocol was also applicable to 4-iodotoluene as a moderately deactivated aryl iodide and the C(sp^2^)–I bond cleavage occurred chemoselectively in the presence of a C(sp^2^)–Br bond. *N*-Methylpyrrole and various other substituted pyrroles could be applied as trapping agents for electron-poor aryl halides and the coupling products were obtained in good yields (52–74%) (Figure 4B). To suppress the rapid HAT with solvent DMF that yields the dehalogenated product, DMSO was chosen as solvent for the C–H arylation. When applying the catalytic protocol to 2-allyloxy-1,3,5-tribromobenzene, the 5-*exo*-*trig* cyclized product **5a** was obtained – albeit only in 28% yield – corroborating a radical mechanism. **PDI** catalysts have since found applications in other chemical transformations, their photophysical properties have been investigated further [40], and new variants [41] including heterogeneous versions have been introduced [42–44].

**Figure 5:**
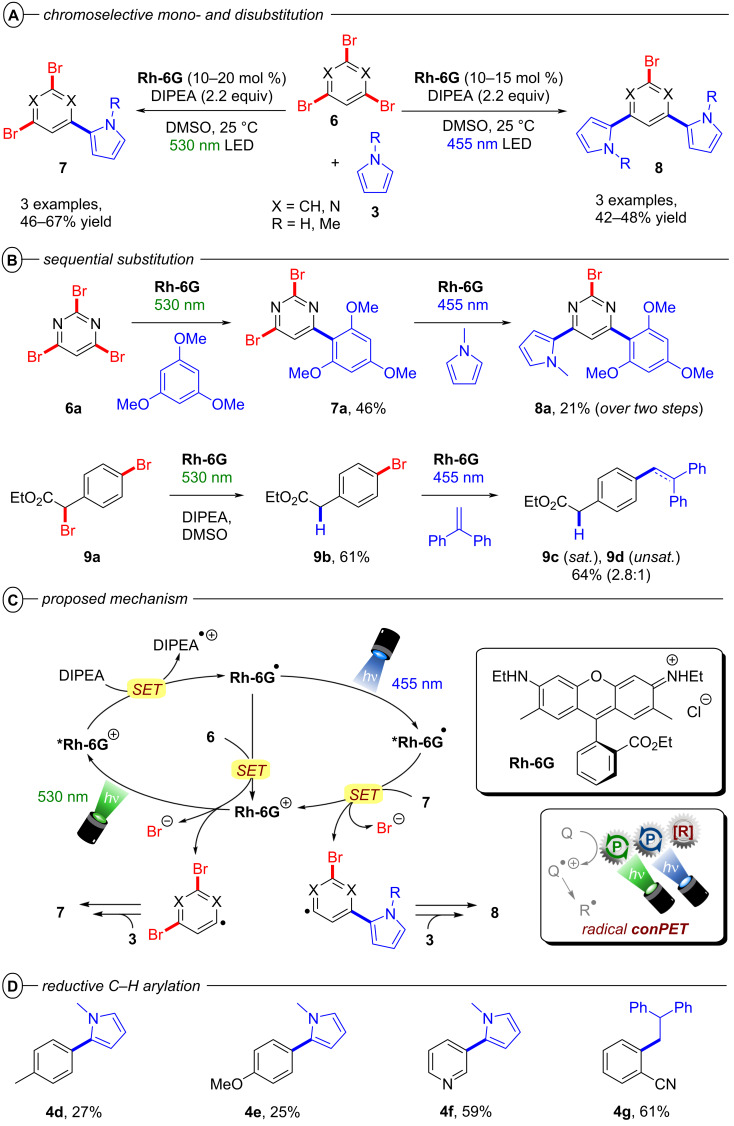
A) Chromoselective mono- and disubstitution or polybrominated pyrimidines with pyrroles. B) Sequential substitution with distinct trapping reagents. C) Proposed mechanism. D) Selected examples from the substrate scope of the C–H arylation via monosubstitution of aryl halides.

Since radical ion conPET chemistry gives access to different reducing species of the same catalyst and both are photoactive, König and co-workers developed a synthetic protocol that allows the chromoselective (wavelength-dependent) regulation of catalytic behavior and thus enabling controlled bond activations [45]. Regarding the reductive C–C arylation, the application of the xanthene dye rhodamine 6G (**Rh-6G**) as a catalyst for the reduction of heteroarenes bearing two or three bromine atoms (e.g., **6**) under irradiation with green light (λ = 530 nm) gave monosubstituted products (e.g., **7**) whereas irradiation with blue light (λ = 455 nm) provided disubstituted products **8** (Figure 5A). Additionally, adding a different trapping reagent before switching from green to blue light allows for a sequential and controlled substitution in a one-pot reaction (Figure 5B). 2,4,6-Tribromopyrimidine (**6a**), whose core pyrimidine structure can be found in many biologically active compounds, could be sequentially substituted with 1,3,5-trimethoxybenzene and *N*-methylpyrrole to give **8a**. The protocol also enabled the selective reductive dehalogenation at the benzylic position of **9a** with green light while the C(sp^2^)–Br bond remained untouched. Subsequent irradiation with blue light gave the sequentially substituted products **9c** and **9d**. As with **PDI**, the xanthene dye rhodamine 6G (**Rh-6G**) can undergo reductive quenching upon excitation with green or blue light (Figure 5C). Considering that **Rh-6G** describes a chloride salt, the photocatalyst itself is a monocationic species (**Rh-6G^+^**) that forms a neutral radical (**Rh-6G^•^**) upon reductive quenching. The radical **Rh-6G^•^** itself (*E*_1/2_ = −1.0 V vs SCE) can directly reduce certain aryl bromides or other substrates with sufficiently accessible reduction potentials whereas a second excitation with blue light yields the excited state *Rh-6G^•^ that can reduce substrates with much more negative reduction potentials (*E*^p^_red_ < −2.4 V vs SCE). The authors also demonstrated the applicability of **Rh-6G** for reductive arylation reactions (Figure 5D). While the use of **PDI** was mostly limited to electron-poor aryl halides, *Rh-6G^•^ could reach a step further and reductively activate electron-rich aryl bromides such as 4-bromotoluene and 4-bromoanisole, albeit providing low (27% and 25%) yields of the coupled products **4d** and **4e**, respectively.

**Figure 6:**
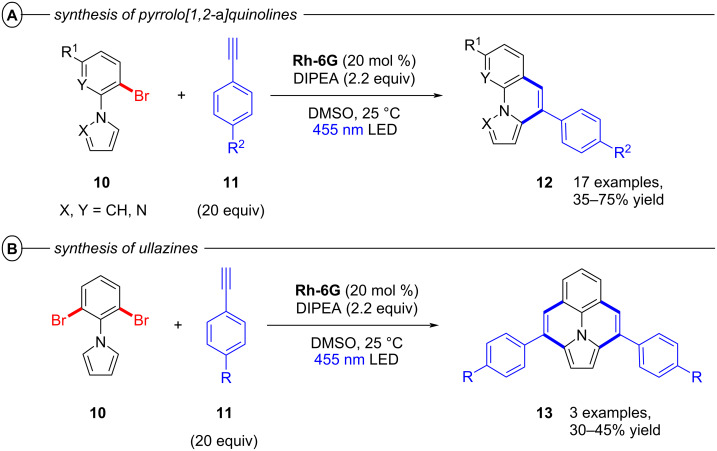
A) Synthesis of pyrrolo[1,2-*a*]quinolines. B) Synthesis of ullazines.

**Figure 7:**
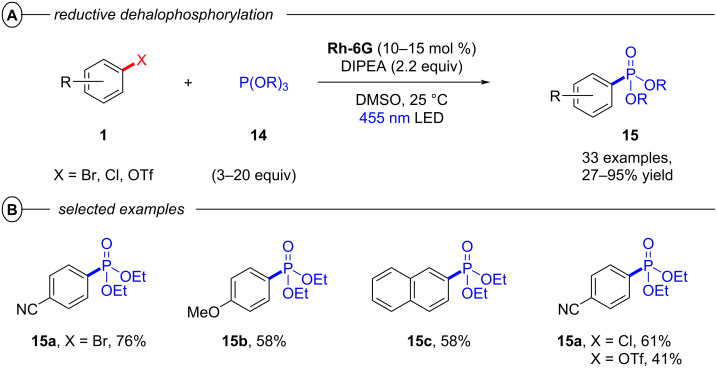
A) Reductive phosphorylation of aryl halides via conPET. B) Selected examples from the substrate scope.

Building on this work, König and co-workers also demonstrated the synthesis of pyrrolo[1,2-*a*]quinolines (**12**) and ullazines (**13**) from *N*-arylpyrroles (**10**) with arylalkynes (**11**) using **Rh-6G** (Figure 6) [46]. Additionally, the König group also used **Rh-6G** as a catalyst for a photo-Arbuzov reaction to generate arylphosphonates (**15**) from aryl halides and trialkylphosphites (**14**) via a similar conPET mechanism (Figure 7) [47]. Notably, even 4-bromoanisole could be reductively activated and phosphorylated in 58% yield (**15b**).

**Figure 8:**
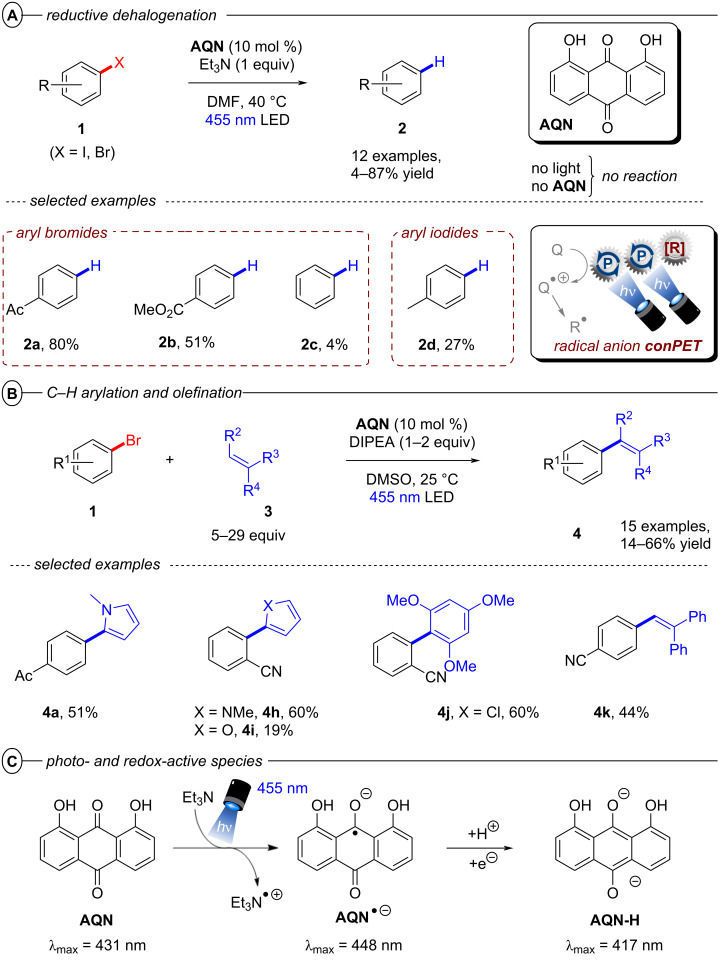
A) Reductive dehalogenation of aryl halides via conPET and selected examples from the substrate scope. B) Reductive C–H arylation and olefination (top) and selected examples from the substrate scope (bottom). C) Photo- and redox-active species of **AQN**.

Reports from Eggins [48], Lund and Eriksen [49] have shown that upon excitation, the radical anions of anthraquinones – a class of organic dyes widely applied as catalysts in organic PRC [50] – are capable of reducing aryl halides with deeply negative reduction potentials. Starting from these premises, the König group in 2017 demonstrated the use of 1,8-dihydroxyanthraquinone (**AQN**) as a suitable conPET catalyst for reductive dehalogenations (Figure 8A), C–H arylations and olefinations of aryl halides (Figure 8B) [51]. In addition to the classical conPET mechanism involving the formation of *AQN^•−^, the authors also confirmed formation of the semiquinone anion **AQN-H^−^** via formal addition of a hydrogen atom (e.g., through protonation and successive reduction or HAT) that upon excitation also acts as a super-reductant (Figure 8C).

**Figure 9:**
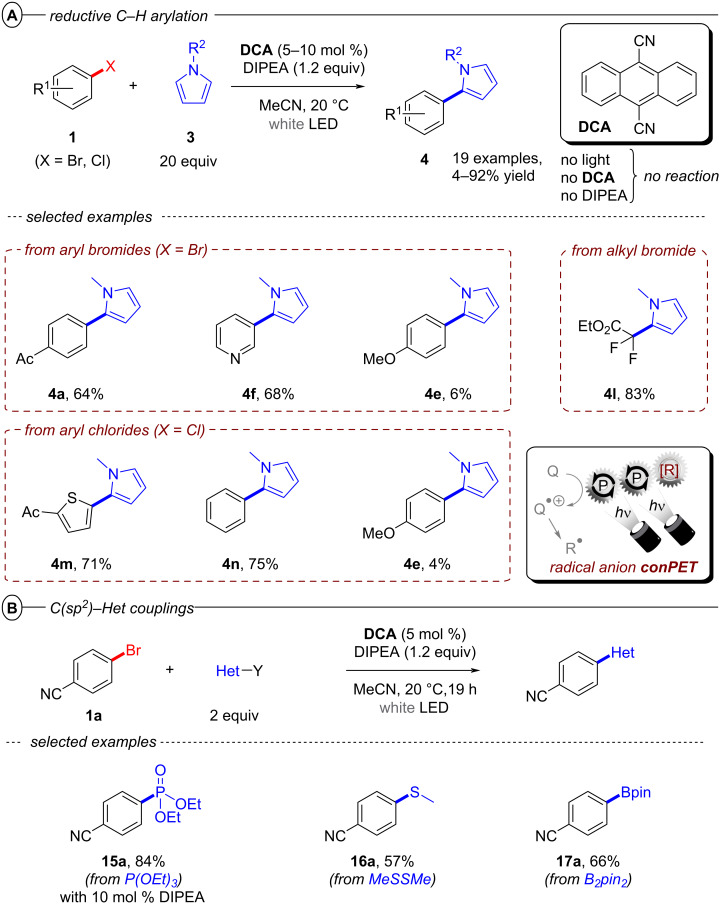
A) Reductive C–H arylation of aryl halides via conPET (top) and selected examples from the substrate scope (bottom). B) Methodology extension to C(sp^2^)–Het(Arene) couplings (top) and selected examples from the substrate scope (bottom).

Simultaneously, Jacobi von Wangelin, Pérez-Ruiz and co-workers introduced the structurally related 9,10-dicyanoanthracene (**DCA**) as a conPET catalyst. Excitation of PET-generated radical anion **DCA^•−^** generates *DCA^•−^ as a super-reductant capable of reducing aryl bromides and chlorides [52]. Due to minimal overlap in the absorption spectral bands of **DCA** and **DCA^•−^**, a cold-white LED (λ = 410–700 nm) was used for polychromatic irradiation. The protocol for reductive C–H arylations with pyrroles was applicable to electron-poor aryl halides including various heterocyclic halides, affording their products in poor to excellent yields (4–92%) (Figure 9A). Regarding the unsatisfactory results, the coupled products of the strongly deactivated 4-bromoanisole (*E*^p^_red_ = −2.75 V vs SCE) and 4-chloroanisole (*E*^p^_red_ = −2.88 V vs SCE) were only obtained in yields of 6% and 4%, respectively, which the authors attributed to insufficient redox power of *DCA^•−^ in its D_1_ state (*E_1/2_ = −2.60 V vs SCE) [53]. These results strongly contrast to the work of Lambert and Lin on e-PRC reductions with **DCA** (vide infra) where these exact electron-rich aryl halides could be engaged successfully.

This contrast suggests either i) a different active species was involved in the latter report (vide infra: Figure 60 in PEC reductive activations) or ii) that the higher steady-state concentrations of **DCA^•−^** available by electrogeneration favor a preassembly with Ar−X that i) upon photoexcitation accesses excited states higher than the first (D_1_) to bolster reactivity and/or ii) following PET assists in the Ar−X**^•^**^−^ fragmentation step. The scope was expanded using triethylphosphite (P(OEt)_3_), dimethyl disulfide (MeSSMe) and bis(pinacolato)diboron (B_2_pin_2_) as trapping agents for C(sp^2^)–Het(Arene) couplings (Figure 9B).

**Figure 10:**
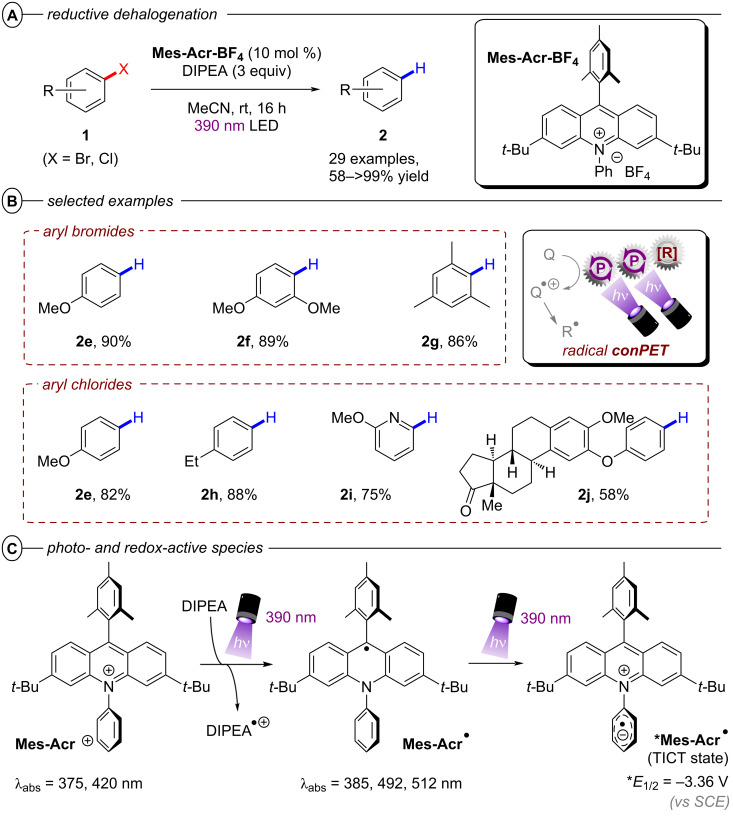
A) Reductive hydrodehalogenation of aryl halides with **Mes-Acr-BF_4_**. B) Selected examples from the substrate scope. C) Photo- and redox-active species of **Mes-Acr-BF_4_**.

The successful activation of electron-neutral and electron-rich aryl halides via conPET mostly remained an unsolved challenge until the Nicewicz group in 2020 disclosed a modified acridinium (Fukuzumi) salt **Mes-Acr-BF_4_** as a suitable conPET catalyst. Following the conPET catalytic cycle, the **Mes-Acr^+^** cation is excited and reductively quenched by DIPEA to yield the acridine radical **Mes-Acr^•^** (Figure 10C) [54]. Upon excitation to its twisted intramolecular charge-transfer (TICT) state, **Mes-Acr^•^** has an excited-state half potential (*E_1/2_ = −3.36 V vs SCE) even more negative than alkali metals including lithium, making it one of the most potent chemical reductants ever reported.

Owing to this exceptional reductive redox power, hydrodehalogenation of various electron-poor and electron-rich aryl bromides and chlorides including 4-bromoanisole and 4-chloroanisole (**2e**), 1-bromo-2,4- dimethoxybenzene (**2f**) and 4-ethylchlorobenzene (**2h**) occurred in good to excellent yields (58–99%) (Figure 10B).

**Figure 11:**
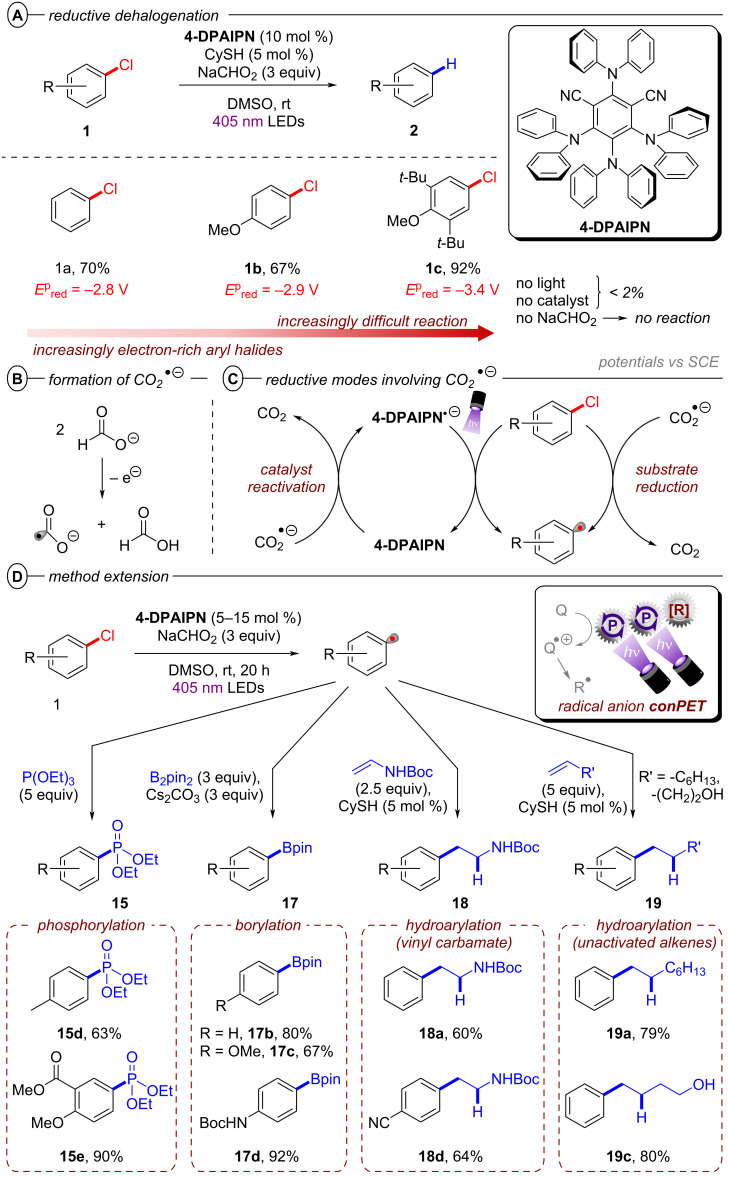
A) Reductive hydrodechlorination of aryl chlorides with **4-DPAIPN**. B) Proposed formation of CO_2_**^•^**^−^. C) Potential reductive modes involving CO_2_^•−^. D) Extension to phosphorylation, borylation and hydroarylation of olefins.

Based on their exciting success with isophthalonitrile derived compounds as electron-primed photocatalysts in e-PRC (vide infra, Figure 69), the Wickens group developed a conPET protocol using 2,4,5,6-tetrakis(diphenylamino)isophthalonitrile (**4-DPAIPN**) for the reduction of electron-rich aryl chlorides [55]. With **4-DPAIPN** as an electron-primed photocatalyst, substrates with reduction potentials as deep as *E*^p^_red_ = −3.4 V vs SCE (**1c**) were readily reduced and dehalogenated products obtained in excellent yields (70–92%) (Figure 11A). Sodium formate was found to be a more efficient terminal reductant than trialkylamines which the authors attributed to the formation of a carbon dioxide radical anion (CO_2_^•−^) upon oxidation of the formate via SET to **4-DPAIPN** and successive deprotonation by a second formate anion (Figure 11B). Due to its reducing nature, CO_2_^•−^ (*E*^0^ = −2.2 V vs SCE) may promote the photoreductant activity either by reducing another equivalent of photocatalyst or the direct reduction of sufficiently electron-poor aryl halide substrates (Figure 11C). While trialkylamines like Et_3_N or DIPEA have been proven to be suitable terminal reductants in conPET chemistry [15,45–46,51,54], they may still decrease photoreductant activity via back electron transfer [56], in contrast to CO_2_^•−^ which has an entropic driving force (evolution of CO_2_). Triethylphosphite P(OEt)_3_ and bis(pinacolato)diboron B_2_pin_2_ were successfully applied as trapping reagents for redox-neutral photo-Arbuzov and borylation reactions with good to excellent yields (Figure 11D). Additionally, the authors were able to perform the net-reductive hydroarylation of *tert*-butyl vinylcarbamate and unactivated alkenes like 1-octene and 3-buten-1-ol although the vinyl carbamate substrate (*E*^p^_red_ = −2.2 V vs SCE) is significantly easier to reduce than most aryl chlorides. This selectivity, especially considering the need for an excess of the vinyl carbamate, might indicate a preassembly between **4-DPAIPN^•−^** and the aryl chloride.

**Figure 12:**
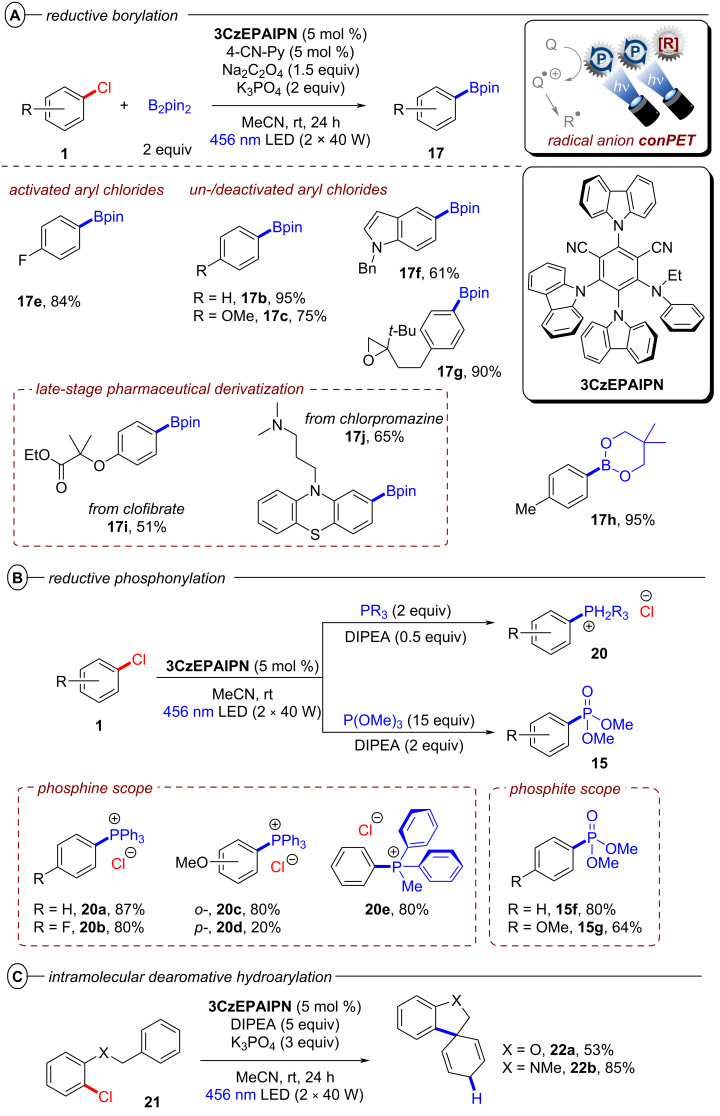
A) Reductive conPET borylation with **3CzEPAIPN** (top) and selected examples from the substrate scope (bottom). B) Phosphonylation scope. C) Intramolecular dearomative hydroarylation scope.

Simultaneously, Zhou, Wu and co-workers demonstrated 2,4,5-tri(9*H*-carbazol-9-yl)-6-(ethyl(phenyl)amino)isophthalonitrile (**3CzEPAIPN**) as yet another isophthalonitrile derived photocatalyst suitable for conPET chemistry [57]. Similar to **4-DPAIPN**, both electron-poor and electron-rich aryl chlorides with reduction potentials up to *E*^p^_red_ = −2.94 V vs SCE were readily reduced by *3CzEPAIPN^•−^. The authors demonstrated an impressive scope of borylation reactions with B_2_pin_2_ as well as other boronate esters (**17h**) and several examples of late-stage functionalization (**17i** and **17j**) (Figure 12A). Interestingly, sodium oxalate could be used as the electron donor provided a catalytic loading of 4-cyanopyridine was added. Although the role of the latter species was not proposed by authors, it is more facile to reduce than an aryl chloride so could act as an electron shuttle (potentially via a π-stacking assembly).

**Figure 13:**
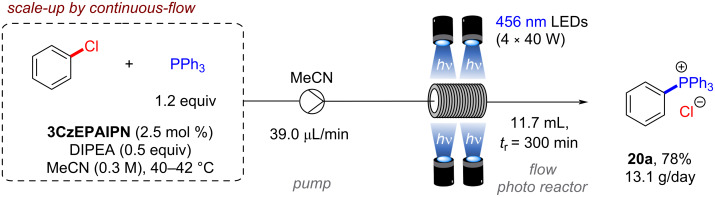
Scale-up of conPET phosphorylation with **3CzEPAIPN**.

The synthetic scope was extended to C(sp^2^)–P bond formations by trapping with phosphines or phosphites (Figure 12B), and in all these cases DIPEA was used as the electron donor (0.5–5 equiv). Arylphosphonium chlorides **20** that are widely used as reagents, organocatalysts, or phase transfer reagents [58–61] were synthesized from aryl chlorides in various yields (20–87%) under mild photocatalytic conditions whereas previously reported protocols typically relied on transition metal catalysis or high temperature processes [62–64]. Arylphosphonates **15** were obtained in a photo-Arbuzov reaction by trapping with trimethylphosphite in good to excellent yields (62–88%) (Figure 12B). Additionally, intramolecular trapping via dearomative hydroarylation gave access to spirocyclic cyclohexadienes bearing dihydrobenzofuran and indoline scaffolds (**22a**,**b**) via a radical-polar crossover mechanism (Figure 12C) [65], showcasing the power of conPET in dearomatization reactions. Finally, the synthesis of tetraphenylphosphonium chloride (**20a**) could be scaled up efficiently in an operationally very simple continuous-flow setup with only 2.5 mol % of photocatalyst and a productivity of 13.1 g/day (Figure 13). Of note, only 0.5 equiv of DIPEA was required for all reactions of aryl chlorides with triarylphosphines, suggesting the intermediate tetraarylphosphine radical reduces DIPEA^•+^ to regenerating DIPEA.

**Figure 14:**
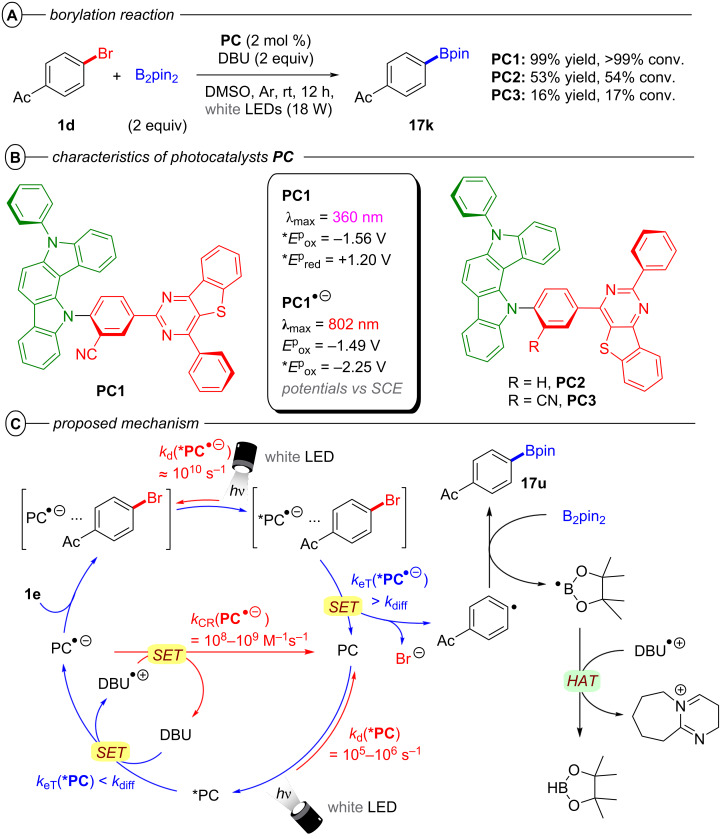
A) Borylation of **1d**. B) Characteristics and structure of **PC1** with green and red parts showing the localization of HOMO and LUMO, respectively. C) Full mechanism for the conPET borylation of **1d** with blue and red arrows indicating activating and deactivating pathways, respectively.

Even though various organic compounds have been successfully implemented as radical (anion) photocatalysts for Ar–X bond activation in the reports of König, Jacobi von Wangelin and Pérez-Ruiz, Nicewicz, Wickens, Zhou and Wu above [15,45–46,51,54–57,66], the underlying mechanism has largely remained elusive. While Kasha’s rule is classically applied only for photophysical phenomena stating that emission events generally occur only from the lowest excited state of a certain multiplicity due to very fast relaxation via internal conversion (IC) and vibronic relaxation [67–69], it can also be adapted to photochemical reactions stating that outer sphere ET events generally occur only from the lowest excited state due to the same relaxation pathways [69]. It has been largely proven that this limitation is circumvented by the involvement of excited radical anions and two excitation processes; to access molecular orbitals beyond the frontier orbitals of the neutral photocatalyst and thus, higher redox potentials. However, the identity of the key intermediate has remained a matter of debate [40,70–71]. Full elucidations of the mechanism toward confirming the key(/main) active catalyst species and possible deactivation pathways are incredibly important for the development of new radical ion catalysts with improved photon economies and novel applications. Lee, Cho, You, and co-workers recently disclosed a fully elucidated mechanism of the reductive borylation of aryl halides using three newly developed photocatalysts bearing indolocarbazole electron donor and benzothienopyrimidine electron acceptor moieties (Figure 14A and B) [72].

In general, three possible pathways can lead to catalyst deactivation and thus, kinetically limit the overall photon economy (Figure 14C, red arrows). Firstly, both photoinduced electron transfer steps are competing with the intrinsic relaxation of the excited states *PC and *PC^•^^−^. In particular, the latter is commonly a very short-lived species (e.g., τ_obs_(*PC1^•^^−^) = 64 ps vs τ_obs_(*PC1) = 2.2 µs). Secondly, even if the initial PET generation of **PC^•^**^−^ succeeds, it can be quickly reversed by charge recombination via unproductive back electron transfer (**PC^•^**^−^ + D^•+^ → **PC** + D), preventing the second excitation. The prevalence of this charge recombination process in conPET effectively regulates a lower steady-state concentration of active photocatalyst compared to PEC where the electrochemical reduction to **PC^•^**^−^ ensures higher concentrations that are directly user-influenced. Upon activation, **PC1** could successfully reduce various aryl halides generating borylated products in modest to excellent (30–99%) yields. Control experiments confirmed that light, catalyst and DBU as a sacrificial electron donor were all essential for product formation. A diminished yield of 19% under aerobic conditions indicates the involvement of a triplet excited state. Addition of i) 2,2,6,6-tetramethylpiperidine-*N*-oxide (TEMPO) as a free-radical quencher or ii) 1,4-dinitrobenzene as an electron trap inhibited product formation which corroborates the involvement of free radicals. The authors argued against radical chain propagation on the basis of lack of reactivity in the dark during the light ON-OFF cycle experiments (we note that this does not rule out chain propagation with an efficient chain death). Investigations of the photon stoichiometry by elucidation of the relation between product yield and light intensity, as well as the insufficient reductive power of both *PC1 (*E_1/2_ = −1.56 V vs SCE) and **PC1^•^**^−^ (*E*_1/2_ = −1.49 V vs SCE) for the reduction of **1d** (*E*^p^_red_ = −1.66 V vs SCE) confirmed the borylation is indeed a two-photon process. DBU was found to quench the steady-state fluorescence of *PC1 with a quenching rate constant two orders of magnitude smaller than the diffusion rate constant in DMSO at 298 K and one order of magnitude greater under the borylation reaction conditions (i.e., 0.20 M DBU) than the intrinsic decay rate of *PC1. Since no quenching by **1d** or B_2_pin_2_ could be observed, the formation of **PC1^•^**^−^ can be attributed exclusively to the thermodynamically favored reductive quenching of *PC1 by DBU. Nanosecond laser flash photolysis techniques were employed to directly monitor the back electron transfer. Second-order kinetics analyses revealed that rapid charge recombination (e.g., *k*_CR_ (**PC1^•^**^−^) = 2.6 × 10^8^ M^−1^ s^−1^) is a significant deactivation pathway in the generation of the key intermediate. This deactivation by back electron transfer taking place in the Marcus-inverted region of electron transfer can be significantly suppressed by using photocatalysts with a more negative reduction half potential *E*_1/2_ (**PC**/**PC^•^**^−^) [73–74].

The involvement of an excited state radical anion *PC1^•^^−^ was further supported by analysis of the product quantum yield (QY). The QY exceeded the theoretical limit of a single-photon process when only taking the absorption of **PC1** into account but gave a reasonable value (Φ_prod_ = 8.2%) for the two-photon process involving excitation of both **PC1** and **PC1^•^**^−^. Electron transfer from *PC1^•^^−^ (*E_1/2_ = −2.25 V vs SCE) to **1d** is thermodynamically favored. While **1d** does not quench the distinctive absorption band (λ_max_ = 802 nm) of **PC1^•^**^−^ (electrochemically generated) in the absence of light, this absorption band rapidly vanished upon irradiation with red light (λ_max_ = 630 nm), corroborating *PC1^•^^−^ as the key catalytic species. The rate constants for SET from *PC^•^^−^ to **1d** were obtained by transient absorption spectroscopy with femtosecond pulsed laser excitation and were 2–3 orders of magnitude greater (e.g., *k*_eT_ (*PC1^•^^−^) = 6.8 × 10^10^ s^−1^) than the diffusion rate in DMSO (*k*_diff_ = 4.0 × 10^8^ s^−1^ of 0.12 M **1d**) confirming a preassociation of **PC1^•^**^−^ and the substrate prior to PET. This is further supported by the inability of *PC3 (*E*_1/2_ = −1.76 V vs SCE) to reduce **1d** in the absence of DBU although this is thermodynamically favored. Additionally, the UV–vis–NIR absorption spectrum of a mixture of **PC1^•^**^−^ and **1d** does not fit the mathematical sum of absorption spectra of both individual compounds but does in fact show additional charge-transfer bands from the preassembly. After electron transfer from *PC1^•^^−^ to **1d**, the C(sp^2^)–Br bond is cleaved and the aryl radical readily reacts with B_2_pin_2_ in a radical substitution reaction yielding the borylated product **17k** and a Bpin radical that is subsequently quenched to HBpin by HAT from DBU^•+^.

**Figure 15:**
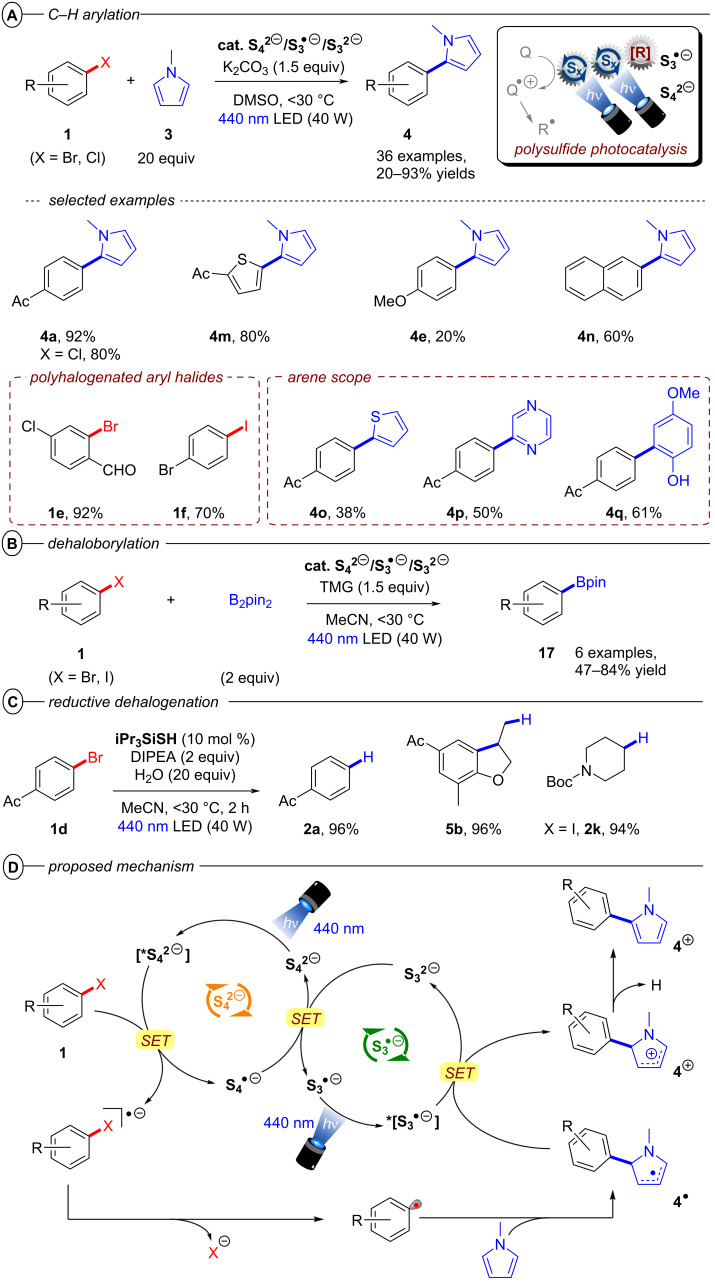
A) Reductive C–H arylation scope with polysulfide conPET (top) and selected examples from the substrate scope (bottom). B) Reductive dehaloborylation. C) Reductive hydrodehalogenation. B) Polysulfide conPET mechanism.

As an alternative to organic radical anion conPET, the Chiba group reported the use of homoatomic polysulfide anions as cheap, readily available and potent photocatalysts [75]. Based on the ground state redox potentials and the visible light absorptions of **S_4_^2−^** and **S_3_^•−^**, the authors developed a catalytic system that employs these species as photoexcited reductants and oxidants in an elegant redox interplay of the **S_4_^•−^**/**S_4_^2−^** and **S_3_^•−^**/**S_3_^2−^** redox couples (Figure 15D). Irradiation of **S_4_^2−^** with blue light generates the potent reductant *[S_4_^2−^] enabling the single electron reduction of aryl halides while simultaneously generating **S_4_^•−^**. Upon C(sp^2^)–X bond cleavage, an aryl radical is formed and trapped by a trapping reagent such as *N*-methylpyrrole, yielding the open-shell species **4^•^**. Upon irradiation of **S_3_^•−^**, the excited species *[S_3_^•−^] oxidizes **4^•^** to the corresponding carbocation **4^+^** while simultaneously generating **S_3_^2−^**. Subsequent deprotonation of **4^+^** yields the C–H arylated product **4** while SET between **S_4_^•−^** and **S_3_^2−^** regenerates the catalytically active polysulfide species **S_4_^2−^** and **S_3_^•−^** and closes both catalytic cycles. Showing high versatility, the direct application of commercially available potassium (poly)sulfide (K_2_S_x_) with H_2_O, the top-down generation from elemental sulfur (S_8_) with sodium *tert*-butoxide (NaO*t*-Bu), and the bottom-up generation from lithium sulfide (Li_2_S) or triisopropylsilanethiol (iPr_3_SiSH) were all suitable methods of catalyst generation for the reduction of aryl halides. Compared to conPET chemistry with organic photocatalysts, no terminal reductants like trialkylamines or formates were required for redox-neutral transformations like the C–H arylation, borylation or phosphorylation owing to the interplay between the two polysulfide redox couples. However, K_2_CO_3_ was needed to quench liberated protons.

A large variety of electron-poor aryl bromides bearing different functional groups readily underwent SET reductions to give biaryl cross-coupled products **4** in poor to excellent yields (20–93%) (Figure 15A). The protocol was also applicable to both electron-rich and electron-poor heteroaryl halides. Due to the inherently higher C(sp^2^)–Cl bond dissociation energies of reductively more inert aryl bromides or aryl chlorides [76], the bottom-up generation of polysulfides from Li_2_S or iPr_3_SiSH was found to provide better results than the use of K_2_S_x_. Unactivated aryl bromides such as 4-bromobiphenyl, 2-bromonaphthalene and 4-bromoanisole could also be reduced, but the sluggish reaction of 4-bromoanisole to **4e** (20% yield) indicates the limit of the reductive power of the polysulfide catalyst system. Notably, several polyhalogenated aromatics could be chemoselectively engaged at one C–X bond, even for 1-bromo-4-iodobenzene (**1f**). Apart from *N*-methylpyrrole, other substituted pyrroles, thiazine (**4o**), pyrazine (**4p**) and electron-rich benzenes (**4q**) were found to be suitable trapping reagents with varying efficiency. The polysulfide catalyst system was also efficiently applied for a dehaloborylation with B_2_pin_2_ (Figure 15B) and a net-reductive hydrodehalogenation (Figure 15C).

**Figure 16:**
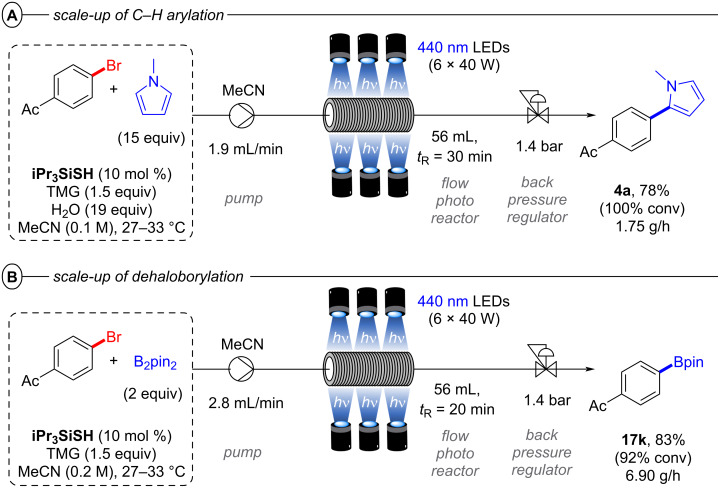
Scale-up of A) C–H arylation and B) dehaloborylation with polysulfide photocatalysis in continuous-flow.

Both the C–H arylation (Figure 16A) and the dehaloborylation (Figure 16B) of aryl chlorides were smoothly transferred to continuous-flow providing products **4a** and **17k** in very good yields and gram-scale per hour productivities demonstrating the ease of scaling up conPET reactions in continuous flow. In general, standardized flow photoreactors which are already widely available enable immediate integration of conPET reactions. On the other hand, PEC reactions require tailor-made reactors that present technical challenges, although in principle these challenges are surmountable by adapting engineering from the already well-established fields of PEC water splitting/fuel cells/photovoltaic fields. So far, the examples of large-scale processes with PEC are limited to the use of recirculated flow or batch (vide infra).

**Figure 17:**
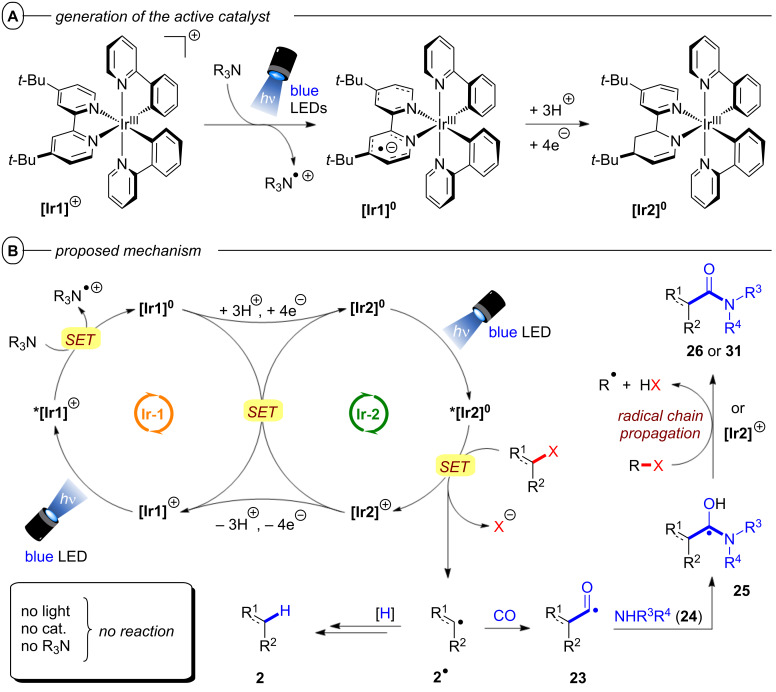
A) Formation of **[Ir1]^0^** and **[Ir2]^0^** upon PET between **[Ir1]^+^** and Et_3_N. B) Mechanism of multi-photon tandem photocatalysis for the hydrodehalogenation and the carbonylative amidation of aryl halides.

As another alternative to organic photocatalysts, the Polyzos group presented a tandem photocatalytic sequence applying **[Ir^III^(ppy)_2_(dtbbpy)]PF_6_** (**[Ir1]^+^**) in combination with Et_3_N to accumulate the energy of two visible-light photons [77]. In their previous work, the Polyzos group discovered the capability of **[Ir1]^+^** to reduce diarylimines via SET in presence of Et_3_N albeit the large difference in the oxidation potential of **[Ir1]^0^** (*E*_1/2_ = −1.47 V vs SCE) and the reduction potentials of imines (e.g., *E*^p^_red_ = −2.18 V vs SCE for *N*-(diphenylmethylene)-1-phenylmethanamine) [78]. Spectroscopic investigations later revealed that the change in absorption and luminescence of deaerated solutions of **[Ir1]^+^** and Et_3_N were neither related to i) the formation of **[Ir1]^0^** via a single-excitation reductive quenching photocatalytic cycle nor ii) *[Ir1]^0^ via a conPET mechanism. Rather, changes related to a chemical transformation of the dtbbpy ligand of the catalyst under the reaction conditions. Charge neutrality and diamagnetism of the new catalyst species, as well as loss of the *C*_2_*_v_* symmetry of **[Ir1]^+^**, indicated the nonsymmetric transformation of the dtbbpy ligand to a monoanionic ligand. Extensive NMR analysis confirmed that upon the formation of **[Ir1]^0^** via SET from Et_3_N to *[Ir1]^+^, partial saturation of the dtbbpy ligand generates **[Ir2]^0^** and initiates the second catalytic cycle (Figure 17A). Upon excitation with blue light, *[Ir2]^0^ reduces aryl halides via SET and is simultaneously oxidized to the Ir^IV^ species **[Ir2]^+^** (Figure 17B). **[Ir2]^+^** then undergoes SET with **[Ir1]^0^** to regenerate both **[Ir2]^0^** and **[Ir1]^+^**, thereby closing both catalytic cycles.

**Figure 18:**
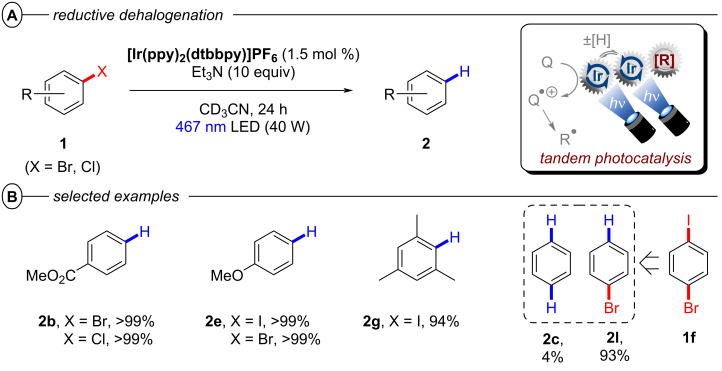
A) Reductive hydrodehalogenation of aryl halides via multi-photon tandem photocatalysis. B) Selected examples from the substrate scope.

An alternative pathway for the regeneration of **[Ir1]^+^** via proton-coupled electron transfer (PCET) from **[Ir2]^+^** could not be ruled out. With no trapping reagents or further reactants present, the aryl radicals generated by C(sp^2^)–X bond cleavage yield hydrodehalogenated products **2** via HAT. Under irradiation with blue light, **[Ir2]^0^** was found to reduce a variety of aryl halides to their hydrodehalogenated products **2** in excellent yields (93–99%) including the electron-rich 4-iodoanisole and 4-bromoanisole that were quantitatively reduced to anisole (**2e**) (Figure 18). Notably, the C(sp^2^)–I bond of 1-bromo-4-iodobenzene was chemoselectively defunctionalized to **2l** in 93% yield under the reaction conditions.

**Figure 19:**
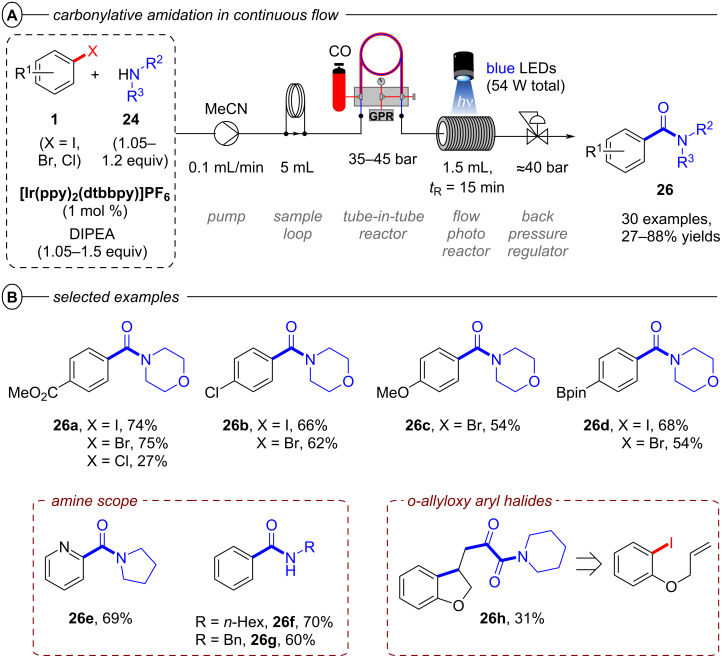
A) Carbonylative amidation of aryl halides in continuous flow. B) Selected examples from the substrate scope.

In 2020, the Polyzos group also demonstrated the carbonylative amidation of aryl halides in continuous flow with the in situ-generated **[Ir2]^0^** (Figure 19A) [79]. This multi-photon tandem photocatalysis protocol provides an elegant alternative to established classical procedures for condensing carboxylic acids with amines that typically generate stochiometric amounts of harmful byproducts released [80–81], while simultaneously operating under milder reaction conditions than those applied in transition metal-catalyzed carbonylative amidation protocols [82–83]. Following the same distinct, yet interconnected photocatalytic cycles as the hydrodehalogenation, an aryl radical **2^•^** is formed via successive PET and C(sp^2^)–X bond cleavage (Figure 17B). Carbon monoxide, introduced to the reaction mixture by a tube-in-tube reactor, traps the aryl radical to generate the acyl radical **23** (Figure 17B). Nucleophilic addition of the amine to the acyl radical and amine-assisted intermolecular proton transfer [84] generates the α-hydroxy radical **24** from which formation of the amide **25** proceeds either via i) oxidation by **[Ir2]^+^** and deprotonation or ii) radical chain propagation [85]. Electron-deficient, electron-neutral, and electron-rich aryl halides bearing different functional groups were all well-tolerated and their products obtained in poor to excellent yields (27–88%). 1-Bromo-4-chlorobenzene and 1-chloro-4-iodobenzene were chemoselectively transformed to **26b** without activation of the C(sp^2^)–Cl bond.

Notably, carbonylative amidation of a borylated aryl bromide to **26d** proceeded well, where a Pd-catalyzed carbonylative amidation reaction would be plagued by undesired Suzuki coupling. Several secondary cyclic and acyclic amines, as well as primary amines were successfully employed as amine coupling partners. The scope of the protocol was further expanded to a radical cyclization/aminocarbonylation cascade reaction yielding the bis-carbonylated α-keto amide **26h** in 31% yield.

**2.1.2 C(sp^3^)–X activation:** The generation of alkyl radicals using alkyl halides as precursors proves very challenging due their deep reduction potentials and bond dissociation energies comparable to aryl halides [86–87]. Classical activation modes for the homolytic C(sp^3^)–X bond cleavage consist of thermolytic or photolytic methods using high temperatures (>220 °C) or irradiation with UV light (λ < 300 nm). These harsh conditions were later replaced by the use of unstable or toxic radical initiators/chain carriers such as peroxides, azo-nitriles, or very prominently Bu_3_SnH [88–89]. Only recently have PRC methods emerged, that mostly relied on the use of metal-based photocatalysts and high energy UV/near-UV light [90–91].

In 2019, the Prato group demonstrated how the **PDI** catalyst first disclosed by the König group (vide supra) could be leveraged for conPET reductions of perfluoroalkyl iodides, providing a photocatalytic alternative for the generation of perfluoroalkyl radicals used in atom transfer radical addition (ATRA) reactions with alkenes [92]. Since the ATRA mechanism involves radical chain propagation, minimal loadings of the **PDI** (0.05 mol %) could be employed as an initiator together with a sub-stoichiometric amount of sodium ascorbate for reductive quenching of **PDI** to generate **PDI^•−^**. While terminal olefin partners were generally well-tolerated, the protocol was limited to perfluoroalkyl iodides. The reductive power of *PDI^•^^−^ (*E_1/2_ = −1.87 V vs SCE) was well-matched to the redox potentials of perfluoroalkyl iodides, but was insufficient for perfluorobromides (e.g., *E*^p^_red_ = −1.52 V vs SCE for CF_3_I and *E*^p^_red_ = −2.10 V vs SCE for CF_3_Br) [93].

**Figure 20:**
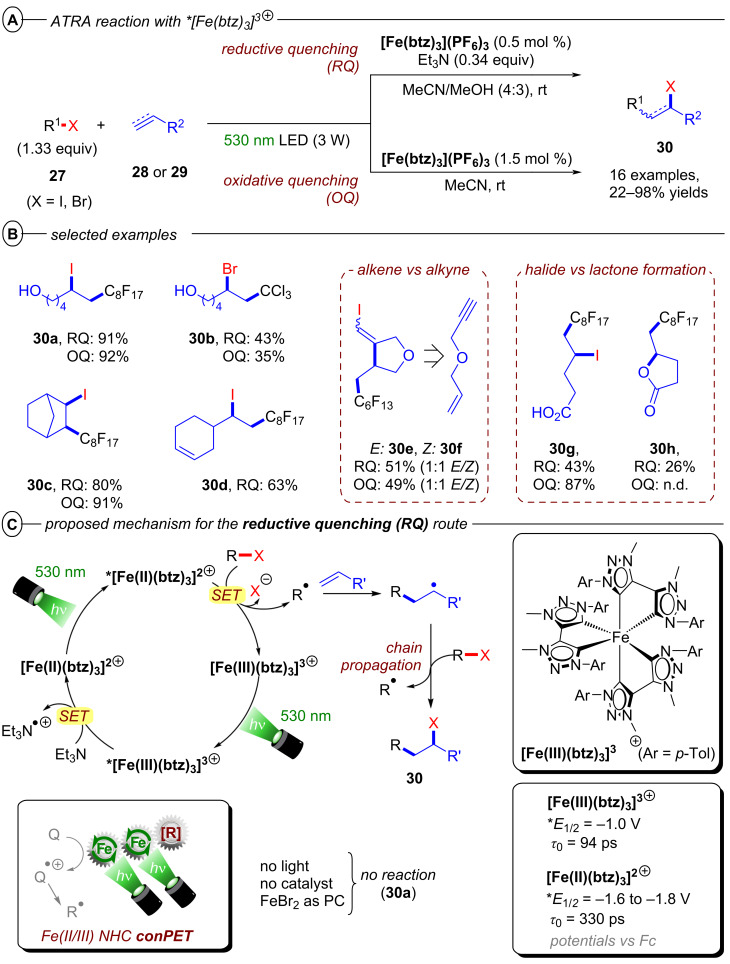
A) General scheme for reductive (RQ) and oxidative quenching (OQ) protocols using **[Fe^III^(btz)_3_](PF_6_)_3_** for the ATRA reaction of alkyl halides with alkenes and alkynes. B) Selected examples from the substrate scope. C) conPET mechanism of the RQ pathway.

As a further application of conPET to atom transfer processes, the Wärnmark group recently disclosed an alternative protocol for the ATRA reaction of perfluoroalkyl iodides using the iron-based NHC complex **[Fe^III^(btz)_3_](PF_6_)_3_** (btz = (3,3’-dimethyl-1,1’-bis(*p*-tolyl)-4,4’-bis(1,2,3-triazol-5-ylidene))) as the first example of an earth-abundant transition metal complex capable of accumulating two photon energies via consecutive ^2^LMCT and ^3^MLCT excitations in an overall conPET mechanism [94]. Since iron-based photocatalysts generally suffer notoriously short excited state lifetimes [95–96], most reactions employing such photocatalysts require special reaction design (e.g., coordination of substrates as ligands to enable intramolecular metal to ligand charge transfer (MLCT)). Only recently have a few examples been reported that observed bimolecular quenching of iron CT states (in the nanosecond domain) enabled by the relatively longer lifetimes of e.g. Fe–NHC complexes [97–100]. In particular, the Wärnmark group reported two sets of conditions with and without Et_3_N as a sacrificial electron donor, to achieve reductive and oxidative quenching pathways, respectively (Figure 20A). Both protocols were able to successfully engage perfluoroalkyl iodides and bromotrichloromethane in combination with a diverse scope of alkenes and alkynes (Figure 20B). Products of terminal alkenes and alkynes were generally obtained in good to excellent yields while also tolerating several functional groups. Substrates bearing internal double bonds were engaged with varying efficiencies (**30c**), but the reaction showed a clear preference for terminal alkenes (**30d**). The reaction of perfluorohexyl iodide with 3-(allyloxy)-1-propyne gave **30e** and **30f** as the only products, demonstrating a clear preference for addition to alkenes even in the presence of alkyne functionalities.

Due to basic conditions of the reductive quenching (RQ) route, the formation of lactone side product **30h** could be observed with a carboxylic acid functionality. In the absence of Et_3_N, the mechanism follows a ‘monophotonic’ oxidative quenching (OQ) route in which **[Fe^III^(btz)_3_]^3+^** is oxidatively quenched to **[Fe^IV^(btz)_3_]^4+^** by the alkyl halide substrate after excitation with green light. After addition of the alkyl radical to the alkene or alkyne substrate, the catalyst is regenerated by oxidizing this radical to the corresponding cation. In the presence of Et_3_N, **[Fe^III^(btz)_3_]^3+^** is reductively quenched after excitation to its ^2^LMCT excited state to generate **[Fe^II^(btz)_3_]^2+^** (equivalent to **PC^•−^** in the classical conPET mechanism) (Figure 20C). **[Fe^II^(btz)_3_]^2+^** is excited again to the more strongly reducing ^3^MLCT excited state (*E_1/2_ = −1.6 V to −1.8 V vs Fc), which then induces SET to the alkyl halide generating an alkyl radical via cleavage of the C(sp^3^)–X bond. The authors propose that this radical then engages in a radical chain propagation pathway leading to product **30** and a new alkyl radical. This is strongly supported by a single turnover experiment, where exclusive excitation of **[Fe^II^(btz)_3_]^2+^** with 700 nm LEDs after in situ generation and substrate addition in the dark generated 5% of product even with only 0.5 mol % of catalyst present. In its ground state, **[Fe^II^(btz)_3_]^2+^** is not reducing enough to engage the model substrate perfluorooctyl iodide and instead *[Fe^II^(btz)_3_]^2+^ was verified as the key catalytic species by transient absorption spectroscopy; even though the strongly reducing α-amino alkyl radical intermediate might also engage in the reduction of alkyl halides or act as a halogen atom transfer (XAT) agent [101].

While yields of the monophotonic oxidative quenching route and the (biphotonic) conPET reductive quenching route were generally well comparable, the RQ route clearly benefits from a longer lifetime of the key catalytic species, shorter reaction times and lower catalyst loading. The requirement for sub-stoichiometric amounts of the sacrificial electron donor can be justified. While the work of Wärnmark is remarkable on a conceptual level, the protocol is still limited by the reductive power of the excited state and thus restricted to rather activated alkyl halides like perfluoroalkyl iodides.

**Figure 21:**
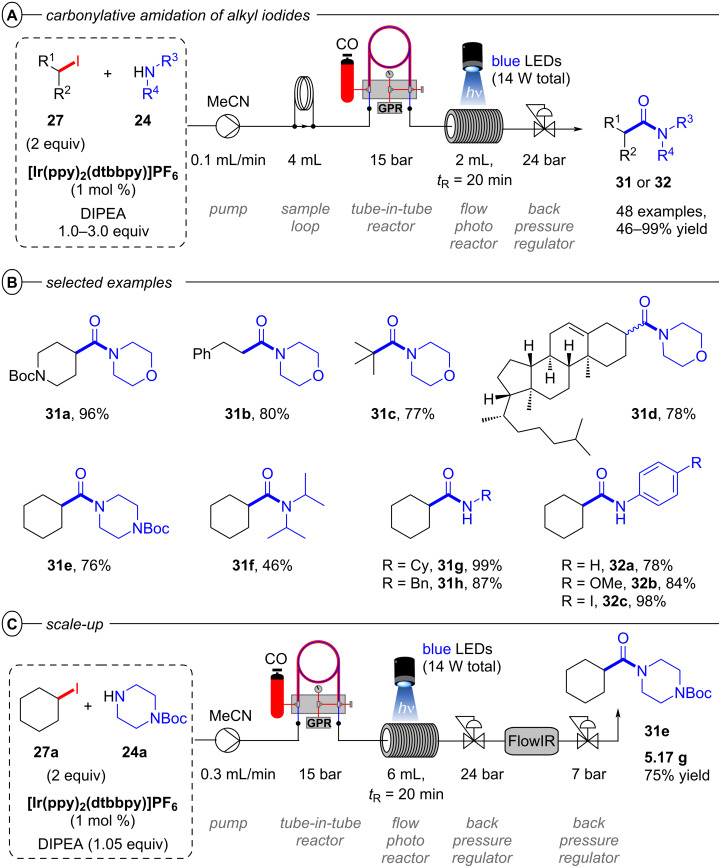
A) Carbonylative amidation of alkyl iodides with **[Ir^III^(ppy)_2_(dtbbpy)]PF_6_**. B) Selected examples from the substrate scope. C) Flow scale up of the **31e**‘s synthesis.

Following their work on carbonylative amidation with **[Ir^III^(ppy)_2_(dtbbpy)]PF_6_** (vide supra), the Polyzos group also disclosed a slightly modified procedure for the carbonylative amidation of alkyl iodides in continuous flow with an impressive substrate scope engaging substrates with notably deep reduction potentials (e.g., *E*^p^_red_ = −2.33 V vs SCE for *n*-butyl iodide) (Figure 21A) [79]. The reaction follows the same tandem photocatalysis mechanism as the carbonylative amidation of aryl halides (Figure 17B).

Tertiary, secondary, and primary alkyl iodides all readily underwent carbonylative amination with morpholine derivatives to afford morpholinoamides in acceptable to excellent yields (46–99%) (Figure 21B). The high yields obtained with primary alkyl iodides are particularly noteworthy due to the competing S_N_2 reaction with amines. Several cholesterol amides were efficiently prepared using this protocol in 1:1 dr (**31d**).

Several primary and secondary amines were successfully employed as coupling partners whereas diisopropylamine led to diminished yield (**31f**), likely due to steric hindrance in reacting with the carbonylated intermediate. Besides, primary anilines bearing a large variety of functional groups proved suitable coupling partners with no obvious influence of the aromatic ring’s electron density on the efficiency (**32a**–**c**). The amenability of *p*-haloanilines **32c** demonstrates orthogonality of this photocatalytic manifold to Pd-catalyzed carbonylative amidation protocols, while competing hydrodehalogenation was not observed as a notable advantage of the former. Scale-up of the reaction employing cyclohexyl iodide (**27a**) and 1-Boc-piperazine (**24a**) gave 5.17 g of **31e** after a collection time of ≈13 h (Figure 21C).

**Figure 22:**
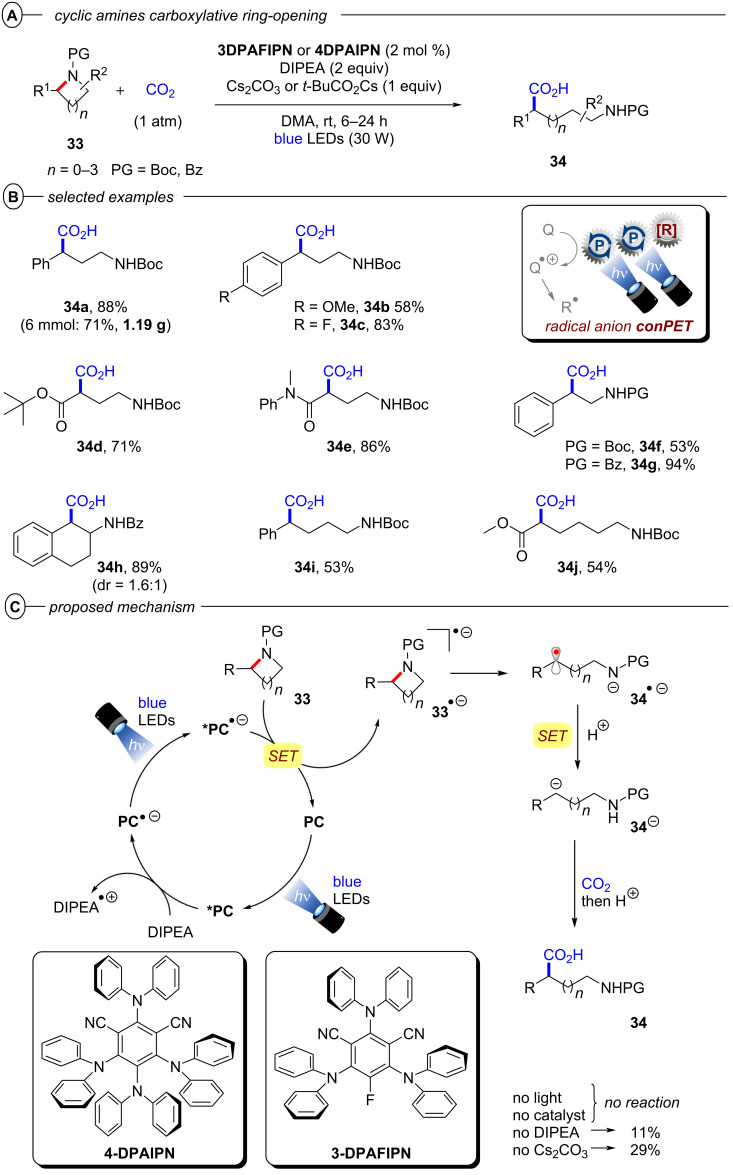
A) Carboxylative C–N bond cleavage in cyclic amines. B) Selected examples from the substrate scope. C) Proposed mechanism.

Apart from C(sp^3^)–halogen bond activations and cleavages, conPET also enables other C(sp^3^)–X bond cleavages. Ye, Yu and co-workers very recently disclosed a protocol for the reductive C–N bond cleavage and carboxylation of cyclic amines **33** for the generation of β-, γ-, δ- and ε-amino acids (generally referred to as **34**) using the isophthalonitrile-derived catalysts **4DPAIPN** and **3DPAFIPN** (Figure 22A) [102]. The authors demonstrated the applicability of the synthetic protocol with a broad substrate scope consisting of 2-arylazetidines and 2-carbonylazetidines for formation of γ-amino acids (42–95%), 2-arylaziridines for formation of β-amino acids (53–94%), pyrrolidines for formation of δ-amino acids (38–66%) and piperidines for formation of ε-amino acids (42–54%) (Figure 22B).

Notably, the reaction of model substrate *N*-Boc-2-phenylazetidine to **34a** could be performed efficiently on a gram scale in 71% yield. Considering that cyclic amines have highly negative reduction potentials (e.g., *E*^p^_red_ = −3.0 V vs SCE for *N*-Boc-2-phenylazetidine), the authors propose that **4-DPAIPN** and **3-DPAFIPN** undergo a conPET cycle for generation of their excited radical anions as the active catalyst (Figure 22C). These highly reducing compounds then engage the amine substrate **33** in a SET reduction. Upon generation of the amine radical anion **33^•−^**, the C–N bond is then cleaved which leads to ring-opening and formation of the benzylic radical **34^•−^**. This was observed in HRMS via TEMPO radical trapping experiments by the authors. The authors propose that protonation (X = H) or carboxylation (X = CO_2_) of this amide anion and a second SET happen either consecutively or in a concerted fashion, leading to formation of a benzylic anion (**34^−^**) which then undergoes carboxylation and protonation to generate the *N*-protected amino acid **34**. An alternative pathway via protonation of **34^−^** and subsequent photocatalytic carboxylation of the benzylic C–H bond with CO_2_ was ruled out.

Interestingly, while conPET technology forges ahead in *reductive* processes for ring opening and functionalization of cyclic amines that has just been summarized, PEC has recently found applications in *oxidative* ring-opening/functionalization of cyclic alcohols, again demonstrating a complementarity between the two methods (vide infra, Figure 64 and Figure 65).

**2.1.3 C(sp^2^)–C(sp^2^) Bond activation: *Alkene activation:*** Since olefins present a fundamental and ubiquitous group of commodity chemicals directly accessible from readily available petroleum feedstocks, their activation via hydrofunctionalization for the construction of saturated hydrocarbon scaffolds is an extensively studied chemical transformation. Classical approaches employ Brønsted acids or transition metal catalysts for electrophilic activation of the target π bond to a cationic or bridged intermediate for subsequent reaction with a nucleophile that generally results in formation of Markovnikov products [103]. Modern orthogonal approaches for the generation of nucleophilic radical anion intermediates relying on PRC, however, are strongly limited in their scope by the highly negative reduction potentials of unactivated olefins (*E*^p^_red_ < −2 V vs SCE) [104–105].

**Figure 23:**
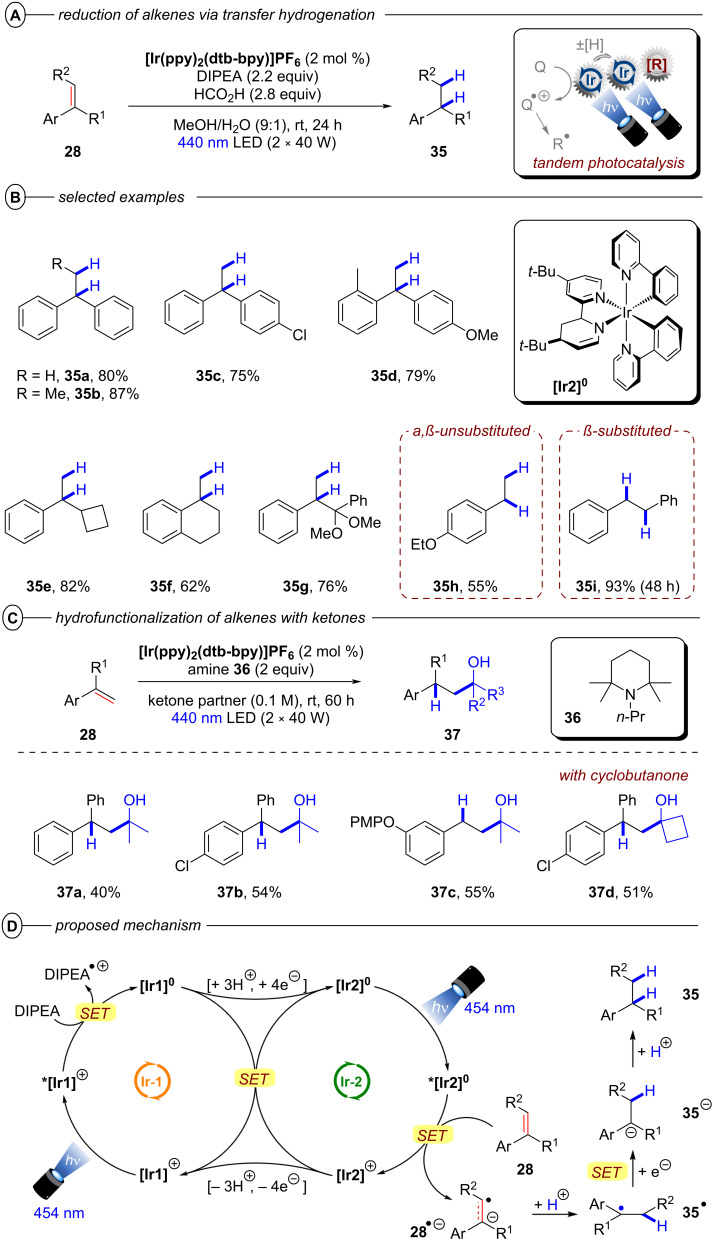
A) Formal reduction of alkenes to alkanes via transfer hydrogenation. B) Selected examples from the substrate scope. C) Hydrofunctionalization of alkenes with ketones and selected examples. D) Proposed mechanism for the reduction of alkenes via tandem Ir photocatalysis with **[Ir^III^(ppy)_2_(dtbbpy)]PF_6_**.

The Polyzos group was able to overcome this limitation by employing their tandem iridium photocatalytic system (vide supra, Figure 17A) for generation of a super-reductants that readily engaged in the reduction of various styrene derivatives via transfer hydrogenation (Figure 23A/B) or by nucleophilic addition to unactivated ketones (Figure 23C) [106]. After its generation via excitation and reductive quenching of **[Ir1]^+^** with DIPEA, **[Ir2]^0^** can be further excited effecting SET reduction of styrenes to their radical anions **28^•−^** (Figure 23D). In the case of transfer hydrogenation, **28^•−^** is then protonated to afford benzylic radical **35^•^**. The alkane product **35** is obtained in the major pathway via a second SET reduction and subsequent protonation, while a major contribution by a concerted HAT from DIPEA^•+^ could be ruled out. Addition of formic acid was required to transform DIPEA to its formate salt and thus suppress unproductive reaction of **28^•−^** and DIPEA^•+^ to form the α-amino adduct. The hydrofunctionalization of ketones to tertiary alcohols **37** likely also involves **28^•−^** as a key intermediate, but the mechanism has yet to be elucidated.

The transfer hydrogenation protocol tolerated both electron-poor and electron-rich 1,1-diarylethylenes. Competing reduction of aryl halide functionalities (**35c**) was not observed. Cyclic and acyclic α-alkyl styrenes, α,β-unsubstituted styrenes and β-substituted styrenes were all suitable substrates. For the hydrofunctionalization of olefins with ketones, the reduction was performed with tetramethylpiperidine derivative **36** as reductive quencher using the ketone coupling partner as solvent.

The reaction proceeded slowly with limited conversion even after 60 h, but the tertiary alcohol products could be obtained in 40–55% yield in a controlled *anti*-Markovnikov manner with both acetone (**37a**–**c**) and cyclobutanone (**37d**).

**2.1.4 Arene activation:** Similar to olefins, arenes and heteroarenes are important, readily available and versatile commodity chemicals produced in large quantities from petroleum feedstocks. While functionalization of arenes (e.g., by substitution reactions) is generally well-investigated, procedures for the dearomatization via reduction to semi-saturated cyclic products remain scarce. The most established and still widely used method for such transformations is the classical Birch reduction, that employs solvated electrons generated by dissolving alkali metals such as lithium and sodium in cryogenic liquid ammonia [107–108]. For the sake of safety and practicability, variations of the Birch reduction under ammonia-free [109–111], transition metal-catalyzed [112], electrochemical [113], or photochemical conditions [114–117] were developed, however, each of these methods require harsh conditions themselves or suffer other strict limitations. Reported photochemical approaches rely on UV irradiation and large stoichiometric loadings of strong reducing agents to overcome the deeply negative reduction potentials of arenes (e.g., *E*^p^_red_ = −3.42 V vs SCE for benzene) [118], rendering such methods unfavorable. While efforts to accumulate visible light photons via conPET provided super-reducing catalysts that should be capable of reducing arenes on thermodynamic grounds, no dearomatized products were observed in the studies on hydrodehalogenation of aryl halides. In 2019, the König group for the first time disclosed a protocol for a Birch-type reduction by visible light iridium photocatalysis employing **[Ir{dF(CF_3_)ppy}_2_(dtbbpy)]PF_6_** (**[Ir^III^]**) in a combined energy-transfer (EnT) and SET process (not shown) [119]. Key to success was leveraging a triplet-triplet EnT from the excited photocatalyst *[Ir^III^] (*E*_T_ = 61.8 kcal mol^−1^) to the substrate, successfully lowering its reduction potential (e.g., *E*_1/2_ = −1.98 V vs SCE vs *E_1/2_ = −0.13 V vs SCE for anthracene). Due to requirement of this EnT step, only substrates with sufficiently low triplet energies accessible to *[Ir^III^] (naphthalenes, etc) could successfully be reduced by this protocol, which excludes arenes with small conjugated system such as benzene (*E*_T_ = 84.4 kcal mol^−1^).

**Figure 24:**
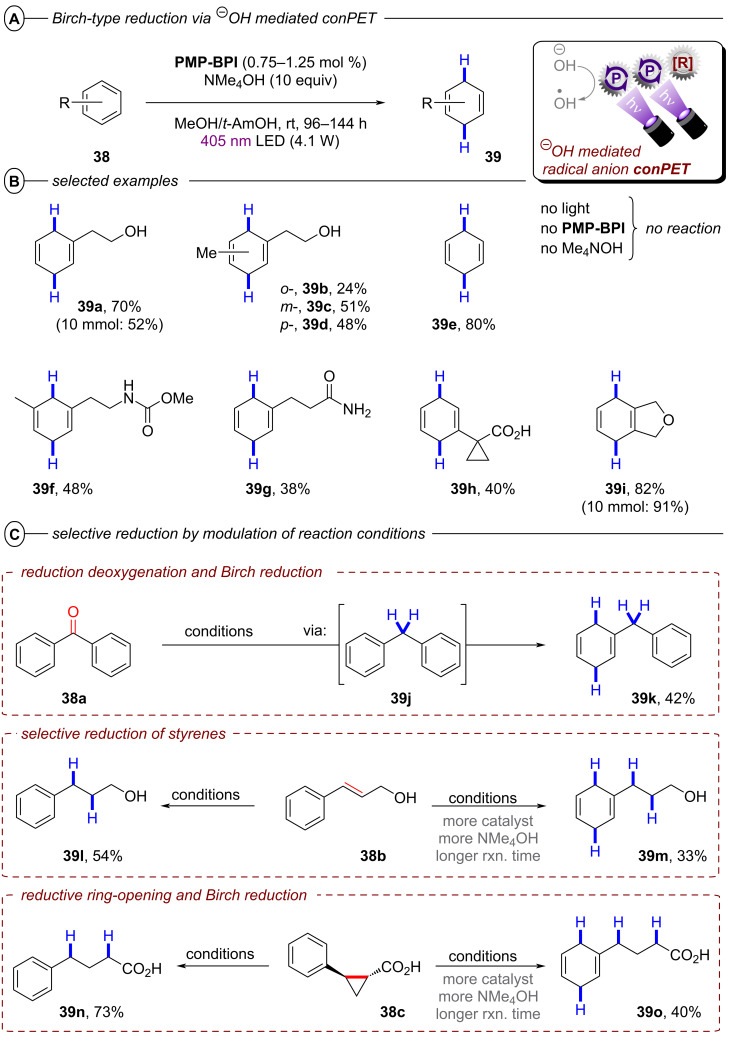
A) Birch-type reduction of benzenes with **PMP-BPI**. B) Selected examples from the substrate scope (scale up reactions under slightly modified conditions). C) Selective reduction by modulation of reaction conditions (reactions conducted under slightly modulated reaction conditions, see original publication for details).

**Figure 25:**
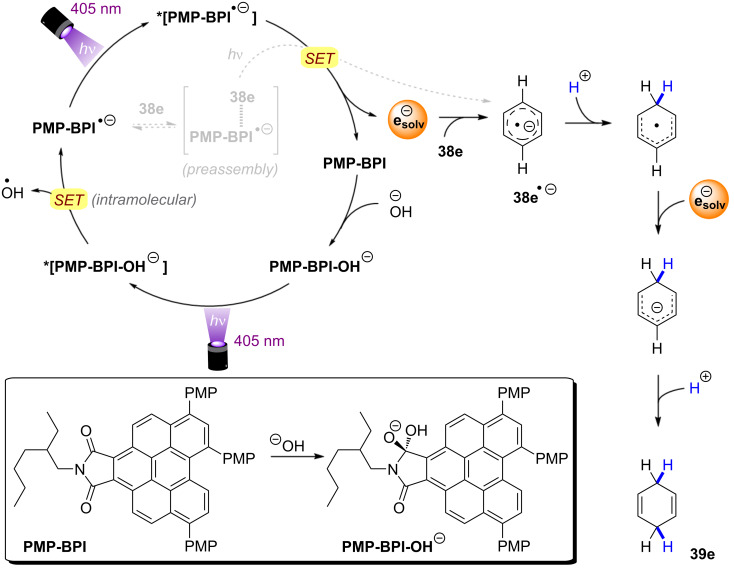
Proposed mechanism of the OH^−^ mediated conPET Birch-type reduction of benzene via generation of solvated electrons.

Miyake and co-workers overcame this issue by a modified conPET mechanism [120]. They demonstrated how benzo[*ghi*]perylene monoimides (**BPIs**) could be successfully reduced to their radical anions via an addition of OH^−^ to the imide, followed by homolysis, whereas commonly applied trialkylamines failed to achieve this. Optimal results were obtained with Me_4_NOH as the electron source for reductive quenching in a solvent mixture of MeOH and *t*-amyl alcohol (*t-*AmOH) under irradiation with blue LEDs (λ = 405 nm). Of the investigated photocatalysts, *p*-OMePh-substituted **BPI** (**PMP-BPI**, see Figure 25 for structure) performed best, even in very low catalyst loadings, but due to presumed catalyst degradation the catalyst had to be added sequentially in three portions (0.25 mol % each) over the long course of the reaction (96 h for most substrates) to achieve sufficient conversion. With optimized conditions, the authors were able to reduce various benzene derivatives (**38**) to the corresponding 1,4-dihydrobenzenes (**39**) in poor to high (24–91%) yields (Figure 24B).

Apart from the model substrate 2-phenylethanol (**39a**) and structurally related compounds, benzene (**39e**) and toluene (not shown) were also readily reduced under the reaction conditions. Several functionalities were well-tolerated, including carbamates, amides and acids, albeit with diminished product yields (e.g., **39f**, **39g** and **39h**). Scale-up to a 10 mmol scale was performed successfully for products **39a** and **39i** (albeit requiring even longer reaction times and 4 × LEDs). The reaction protocol was not applicable to electron-rich arenes, substrates bearing alkene, alkyne, alkyl halide or unprotected amine functionalities and N-containing heterocycles.

The successful reduction of benzene and toluene via this protocol is the first and so far only example of a visible-light photocatalytic Birch-type reduction that could engage these simple aromatic feedstock compounds. Modification of the reaction conditions, in particular amounts of catalyst and NMe_4_OH and reaction time allowed for selective reductions (Figure 24C). Benzophenone underwent tandem reductive deoxygenation and Birch-type reduction to **39k**. Cinnamyl alcohol could either be reduced on the alkene functionality to **39l** or further reduced to **39m** while reductive ring-opening of *trans*-2-phenylcyclopropane-1-carboxylic acid could be modified to yield either **39n** or the Birch-type reduction product **39o**.

Mechanistic investigations revealed that generation of the key catalytic species proceeds via addition of OH^−^ to **PMP-BPI**, generating **PMP-BPI-OH^−^** (Figure 25). After irradiation, a hydroxyl radical is eliminated and **PMP-BPI^•−^** can be excited again to *[PMP-BPI^•−^]. While the fate of the hydroxyl radical is unknown, DFT calculations found this PET occurring from the lowest singlet excited state of **PMP-BPI-OH^−^** to be exergonic by 11.5 kcal mol^−1^ (under irradiation with 405 nm LEDs). **PMP-BPI^•−^** that was generated via mediation with hydroxide or fluoride or even by bulk electrolysis (*E*_cell_ = −2.26 V vs SCE) all showed the same absorption. A second photoexcitation is required due to the insufficient reducing power of ground state **PMP-BPI^•−^** (*E*_1/2_ = −1.24 V vs SCE). Based on DFT calculations and nanosecond transient absorption spectroscopy, the authors favored a plausible mechanism via the generation of a solvated electron, rather than direct SET from the photocatalyst. The prolonged lifetime of *PMP-BPI^•−^ observed in nanosecond transient absorption spectroscopy with excitation at 532 nm is assumed to stem from an unreactive quartet excited state *^4^PMP-BPI^•−^, arising from ISC from the doublet state *^2^PMP-BPI^•^^−^. Owing to the absence of significant quenching of this long-lived quarted state by arene substrates, the authors proposed instead that photoexcitation to the first doublet state (or higher order doublet states) *^2^PMP-BPI^•^^−^ liberates a solvated electron. The solvated electron reduces the arene substrate to its radical anion **38^•−^** and the 1,4-dihydrobenzene product **39** is obtained by sequential protonation, reduction by another solvated electron and a second protonation.

Alternatively, the authors could not rule out reduction of arene substrates occurring via direct SET from a higher excited state of *PMP-BPI^•−^ (D_n_) in an anti-Kasha fashion, which would require a catalyst-substrate preassembly. Given the selectivity observed for arene over amide/carbamate reductions and the extended π-system of **PMP-BPI^•^**^−^, a π–π stacking interaction assembly seems plausible. However, unlike the conPET study of Lee, Chou, You and co-workers [72] and like the e-PRC study of Barham, König and co-workers (vide infra, Figure 72), the authors did not find any evidence for such an assembly of **PC^•^**^−^ by UV–vis spectroscopy. Barham, and co-workers as well as Hauer and co-workers (vide infra, Figure 37 and following discussion) showed in their studies of organic radical cations that a lack of change in steady-state UV–vis spectroscopy cannot speak against a preassembly – especially with non-polarized/electronically symmetrical substrates – and *only transient absorption spectroscopy kinetics is qualified to reveal the necessary operation of a preassembly for radical ion photocatalysis*.

**Figure 26:**
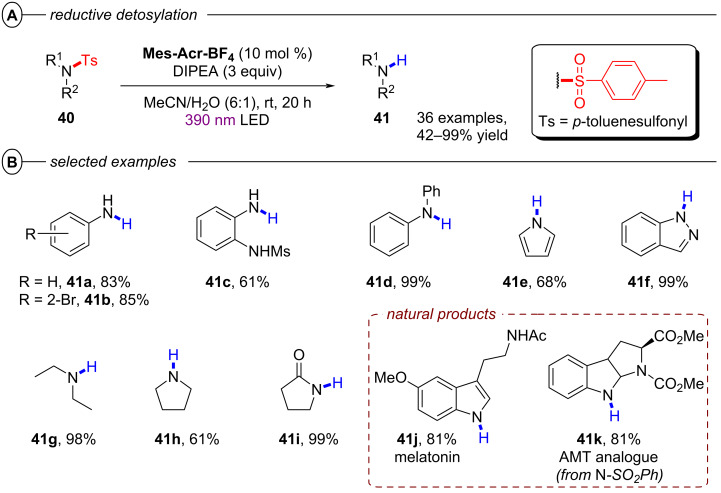
Reductive detosylation of *N*-tosylated amides with **Mes-Acr-BF_4_**. B) Selected examples from the substrate scope.

**2.1.5 Het–Het bond activation: *N–S and O–S bond activation:*** In their work on reductive hydrodehalogenation of aryl halides, the Nicewicz group also disclosed a protocol for reductive detosylation of *N*-tosyl amides with **Mes-Acr-BF_4_** via a conPET mechanism [54]. Single-electron reduction of a tosylated amine **40** by the twisted intramolecular charge transfer (TICT) state of the **Mes-Acr^•^** radical (Figure 10C) yielded its radical anion, which supposably eliminated a tosylate anion to generate a primary or secondary aromatic or aliphatic amines **41** (Figure 26A). Primary and secondary tosylated anilines were efficiently transformed to the corresponding anilines in moderate to excellent yields (42–99%). Detosylation occurred selectively in presence of a mesylated amine functionality (**41c**). Alkylamines were readily generated from their tosylated counterparts (Figure 26B). Pyrrolidine was obtained in 61% yield (**41h**) whereas the introduction of an adjacent carbonyl group increased the yield to 99% (**41i**). Several heterocyclic amines including pyrrole (**41e**) and 1*H*-indazole (**41f**) as well as natural products like melatonin (**41j**) or the AMT analogue **41k** were also successfully engaged in reductive detosylation.

The Wenger group recently disclosed a dual photoredox catalytic approach for the reductive detosylation of *N*-tosyl amides **40** to secondary amines **41** [121]. Herein, photoexcited **[Cu(dap)_2_]Cl** assists in the generation of **DCA^•−^** (from **DCA**) which can be photoexcited to act as a super-reductant. The generation of **DCA^•−^** by the Cu catalytic cycle without direct excitation of **DCA** allows for irradiation with red light whereas the classical conPET mechanism (vide supra, Figure 9) requires near-UV/blue light irradiation to excite **DCA** [52]. Red photons (for λ = 620 nm, *E* = 2.0 eV) intrinsically possess substantially lower energy than blue (for λ = 410 nm, *E* = 3.0 eV) or green photons, which gives the impression they may be unfavorable for photocatalytic reactions. To the contrary, however, the use of red light provides other advantages including less photodamage and greater penetration depth into colored reaction solutions [122]. Furthermore, a dual catalytic system with two fully independent photocatalysts provides increased possibilities for method optimization and development but at the same time requires more careful reaction design. The authors initially developed their dual PRC protocol for the benchmark hydrodehalogenation of various aryl halides and efficiently transformed several moderately difficult-to-reduce substrates to their dehalogenated products.

**Figure 27:**
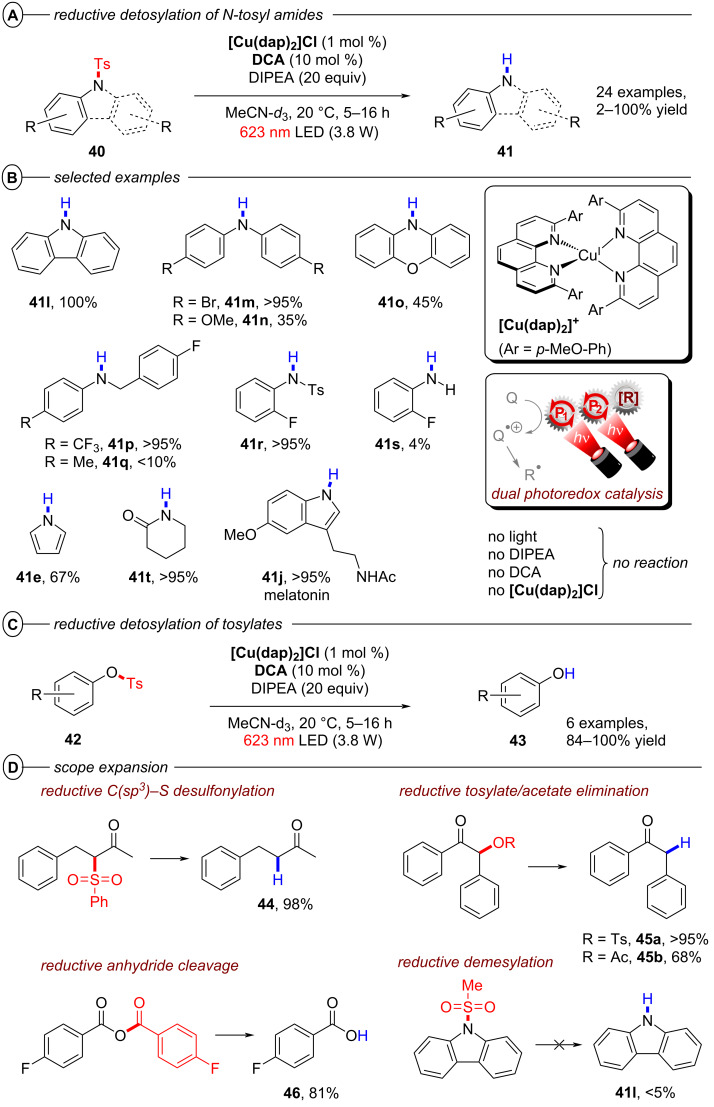
A) Reductive detosylation of *N*-tosyl amides by dual PRC. B) Selected examples from the substrate scope for the detosylation of *N*-tosyl amides. C) Reductive detosylation of tosylates. D) Scope expansion to other reductive transformations achieved by dual PRC.

*N*-Tosyl amides were also suitable substrates, affording detosylated products in mostly good to even quantitative yields with a few exceptions (Figure 27A, B). Several carbazoles (**41l**) and diarylamines (**41m**) were readily generated, however, aryl ethers such as di(*p*-anisyl)amine (**41n**) and phenoxazine (**41o**) were only obtained in modest yields (35–45%). The efficiency seems to be influenced by electronic effects; an electron-poor benzylic (trifluoromethyl)aniline (**41p**) resulted in >95% yield whereas a more electron-rich benzylic toluidine (**41q**) gave <10% yield. A ditosylated aniline was selectively detosylated once to afford **41r** in >95% yield which is in good accordance with the low conversion observed for the detosylation of **41r** to **41s**. Several heteroaromatic amines including pyrrole (**41e**) were also generated in good to excellent yields (67–98%). Electron-withdrawing carbonyl functionalities enabled the efficient detosylation of cyclic aliphatic amines (e.g., **41t**) whereas detosylation of unsubstituted pyrrolidine gave no product. Several tosylated phenols including a doubly tosylated substrate were efficiently detosylated under the reaction conditions (Figure 27C). Additionally, selected examples demonstrate the large scope of the dual catalytic system including reductive C(sp^2^)–S desulfonylations (**44**), eliminations of tosylates (**45a**) and acetates (**45b**) and reductive anhydride cleavages (**46**) (Figure 27D). Reduction of a mesylated carbazole was not viable and resulted in minimal formation of product **41l**.

**Figure 28:**
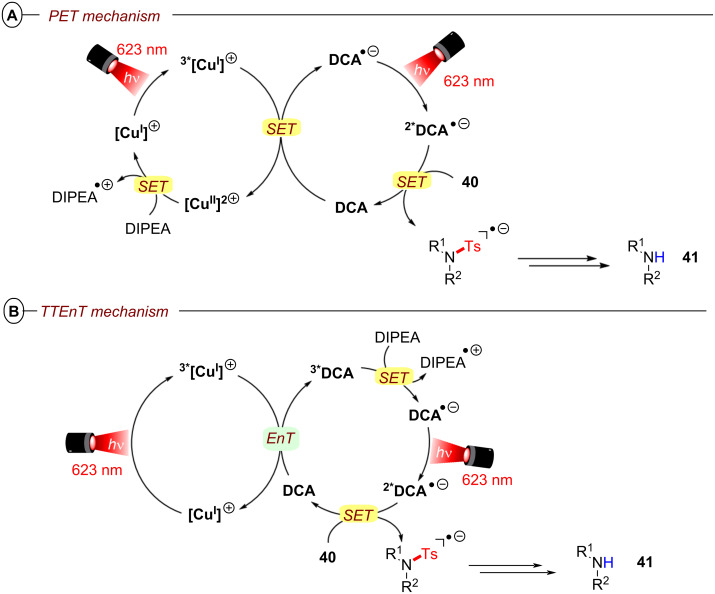
A) Mechanism of the dual PRC based on PET between **[Cu(dap)_2_]^+^** and **DCA**. B) Mechanism of the dual PRC based on TTEnT between **[Cu(dap)_2_]^+^** and **DCA**.

The authors proposed two possible mechanisms for the dual PRC with **[Cu(dap)_2_]Cl** and **DCA**; one relying on PET (Figure 28A) and another relying on triple-triplet energy transfer (TTEnT) (Figure 28B) as the interconnection between both catalytic cycles. For the PET mechanism (Figure 28A), **[Cu^I^(dap)_2_]^+^** (**[Cu^I^]^+^**) is initially photoexcited to ^3^*[Cu^I^]^+^ which then undergoes SET to **DCA** to directly access **DCA^•−^**. **[Cu^I^]^+^** is subsequently regenerated via reductive quenching of **[Cu^II^]^2+^** by DIPEA to close the Cu catalytic cycle whereas **DCA^•−^** is excited to ^2^*DCA^•−^ by the absorption of a red photon. A second SET between ^2^*DCA^•−^ and the substrate regenerates **DCA** and generates the substrate radical anion. In the TTEnT mechanism (Figure 28B), **DCA^•−^** is formed in a SenI-ET via the generation of ^3^*DCA through energy transfer of ^3^*[Cu^I^]^+^ to **DCA** and subsequent reductive quenching by DIPEA. As before, **DCA^•−^** is then excited to ^2^*DCA^•−^ to enable substrate reduction via SET.

Based on cyclic voltammetry, spectroscopic and spectroelectrochemical data, it was concluded that both pathways are thermodynamically feasible and could simultaneously operate in MeCN as the reaction solvent due to spectroscopic evidence for **[Cu^II^]^2+^** and ^3^*DCA. Based on relative intensity and molar extinction coefficients of the absorption by **DCA^•−^** and ^3^*DCA upon excitation of **[Cu^I^]^+^**, the authors provided estimations for the concentrations of both species and thus, estimated the relative prevalences of the PET and TTEnT mechanism in MeCN and acetone. Lower concentrations of ^3^*DCA than of **DCA^•−^** in MeCN suggest that the PET mechanism herein accounts for roughly 70% of product formation while higher relative concentrations of ^3^*DCA in acetone indicate that TTEnT dominates here. Although this proposal has to be judged with caution due to severe simplifications, the study nonetheless emphasizes the critical influence of solvation on photocatalytic mechanisms and their elucidation.

**Figure 29:**
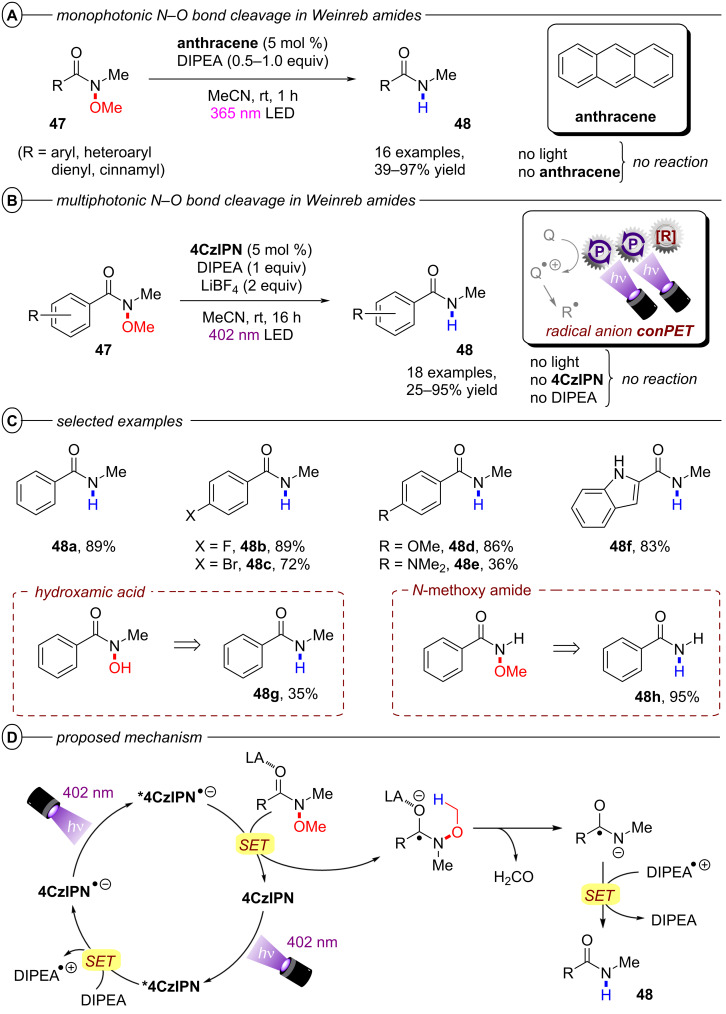
A) N–O bond cleavage in Weinreb amides with anthracene. B) N–O bond cleavage in Weinreb amides relying on conPET. C) Selected examples from the substrate scope. D) Proposed conPET mechanism of the N–O bond cleavage in Weinreb amides with **4CzIPN** and LiBF_4_ as a Lewis acid (LA).

***N–O Bond activation:*** The Gilmour group recently disclosed a synthetically simple photocatalytic protocol for the reductive cleavage of N–O bonds in Weinreb amides **47** for generation of *N*-methyl amides **48** [123]. Initially, the authors employed anthracene as the photocatalyst and DIPEA as its reductive quencher in a reductive quenching PET cycle, to afford the anthracene radical anion as a potent reductant (*E*_1/2_ = −1.95 V vs SCE) (Figure 29A). This monophotonic protocol – relying on the energy of a single UV light photon – was found to be sufficiently powerful to reductively cleave several cinnamyl and dienyl Weinreb amides, as well as electron-deficient aryl and heteroaryl Weinreb amides, in moderate to excellent (39–97%) yields. However, electron-neutral or electron-rich aryl Weinreb amides could not be engaged as a redox limitation. This issue was addressed from two distinct strategies to expand the scope of the transformation. On the one hand, Lewis acid activation of amides with LiBF_4_ significantly facilitated their SET reduction. On the other hand, the reductive power of the photocatalyst was increased by replacing anthracene with **4CzIPN** as a photocatalyst capable of accessing a highly potent reductant (proposed to be *4CzIPN^•−^) via a conPET mechanism.

Under the modified procedure, electron-rich aryl Weinreb amides **47** were efficiently transformed to the corresponding *N*-methyl aryl amides **48** in poor to excellent (25–95%) yields (Figure 29B/C). Unsubstituted, halogenated, methylated, and arylated *N*-methyl benzamides were generally readily engaged, however, substituents in *ortho-* and *meta-*position decreased the yield compared to *para*-substitution. The more electron-rich *p*-methoxy-*N*-methoxy-*N*-methylbenzamide (*E*^p^_red_ = −2.27 V vs SCE) readily engaged in the reaction and gave 86% yield (**48d**) while *p*-amino-*N*-methylbenzamide was only obtained in 36% yield (**48e**). Additionally, the hydroxamic acid *N*-hydroxy-*N*-methylbenzamide and *N*-methoxybenzamide were exposed to the reaction conditions and successfully transformed to products **48g** and **48h**. A mechanistic proposal for the Lewis acid-assisted reductive N–O bond cleavage following SET from *4CzIPN^•−^ via conPET is shown in Figure 29D, and was supported by a near quadratic relationship between light intensity and yield. The conPET strategy also allowed for longer wavelength light to be employed, however, the energy efficiency benefit of generating one single UV photon vs two near-UV/purple photons is debatable.

#### 2.2 Oxidative activation

While reductive bond activations via conPET – in particular the reduction of aryl (pseudo)halides – saw considerable development following König’s seminal report on **PDI** (vide supra) [15], oxidative activations of substrates via conPET have only recently been demonstrated and remain largely underexplored to date. Since the oxidation potentials of unactivated or deactivated olefins or arenes lie at (or beyond) the energy threshold of single-photon PRC, the development of highly oxidizing photocatalysts is highly desirable to provide access to facile functionalization of these important and abundant feedstock chemicals. While it has been demonstrated that classical chemical oxidant 2,3-dichloro-5,6-dicyano-1,4-benzoquinone (**DDQ**) can function as a closed-shell photocatalyst with an excited-state reduction potential ≈+3 V vs SCE and can oxidatively engage electron-deficient arenes [124–125], the application of **DDQ** is rather undesirable due to the requirement for larger catalyst loadings (10–20 mol %) [126], the use of *tert*-butyl nitrite co-oxidant that forms explosive mixtures in air, evolution of HCN gas upon contact with moisture [127] and catalyst degradation via side reactions with certain amines under the reaction conditions [124]. These limitations are easily overcome by i) oxidative conPET or ii) the merging of photo- and electrochemistry through the use of anodic oxidation (vide infra, especially for **DDQ**) and the former will now be presented.

**Figure 30:**
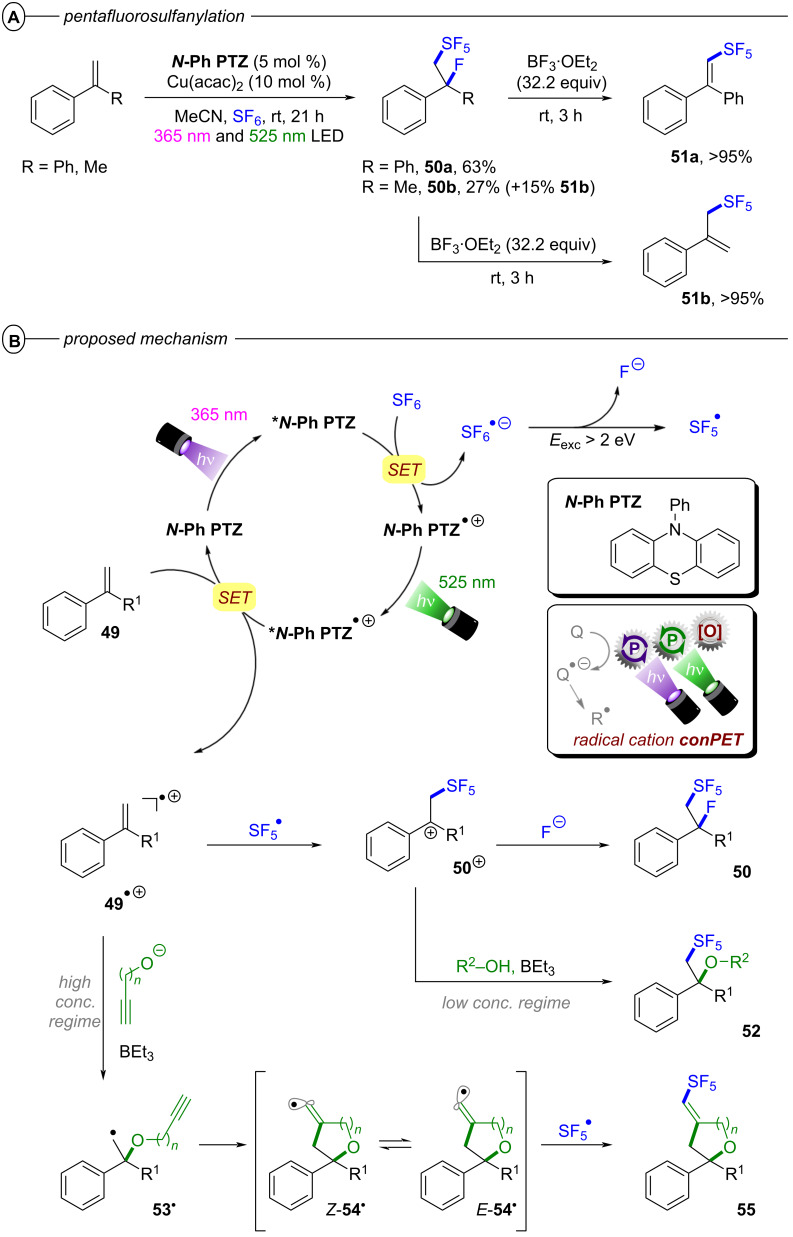
A) Pentafluorosulfanylation and fluoride elimination. B) Mechanism of the pentafluorosulfanylation and α-alkoxypentafluorosulfanylation of α-substituted styrenes with SF_6_.

**2.2.1 Alkene activation:** In 2018, the Wasielewski group demonstrated the super oxidative power of the excited doublet states of the radical cation of *N*-phenylphenothiazine (***N*-Ph PTZ^•+^**), which set the scene for ***N*-Ph PTZ** as a photocatalyst for oxidative conPET [128]. Simultaneously, Wagenknecht disclosed a protocol for ***N*-Ph PTZ** as a photocatalyst for the pentafluorosulfanylation of diphenylethylene (**49a**) and α-methylstyrene (**49b**) with SF_6_ as both terminal oxidant and coupling partner (Figure 30A) [16]. In the mechanism, the radical cation ***N*-Ph PTZ^•+^** is generated upon oxidative quenching of *N-Ph PTZ by SF_6_ (Figure 30B). Thereafter, SF_6_**^•^**^−^ breaks down into a fluoride ion and a pentafluorosulfanyl radical (SF_5_^•^) while ***N*-Ph PTZ^•+^** is photoexcited to *N-Ph PTZ^•+^ which then oxidizes the styrene substrate to its radical cation **49^•+^**. With no other suitable trapping agents present, **49^•+^** is trapped by SF_5_^•^ and reaction of the resulting cation **50^+^** with a fluoride ion then generates product **50** (Figure 30B). Subsequent elimination of a fluoride ion mediated by BF_3_·OEt_2_ forms pentafluorosulfanylated styrenes **51a** and **51b** (Figure 30A).

While photocatalytic SET reduction of SF_6_ had been achieved previously with **Ir(ppy)_2_(dtb-ppy)PF_6_** by Jamison’s group [129], the fragmentation pattern of SF_6_^•−^ was proposed to be highly dependent on the excess energy provided by SET. The less potent reducing iridium species in Jamison’s work favors the lower energy fragmentation pathway to F^•^ and SF_5_^−^ whereas highly potent reductant *N-Ph PTZ provides enough energy for access to SF_5_^•^ and thus, pentafluorosulfanylation [16,130]. Addition of catalytic amounts of Cu(acac)_2_ was found to favor product formation by suppressing undesired side reactions.

**Figure 31:**
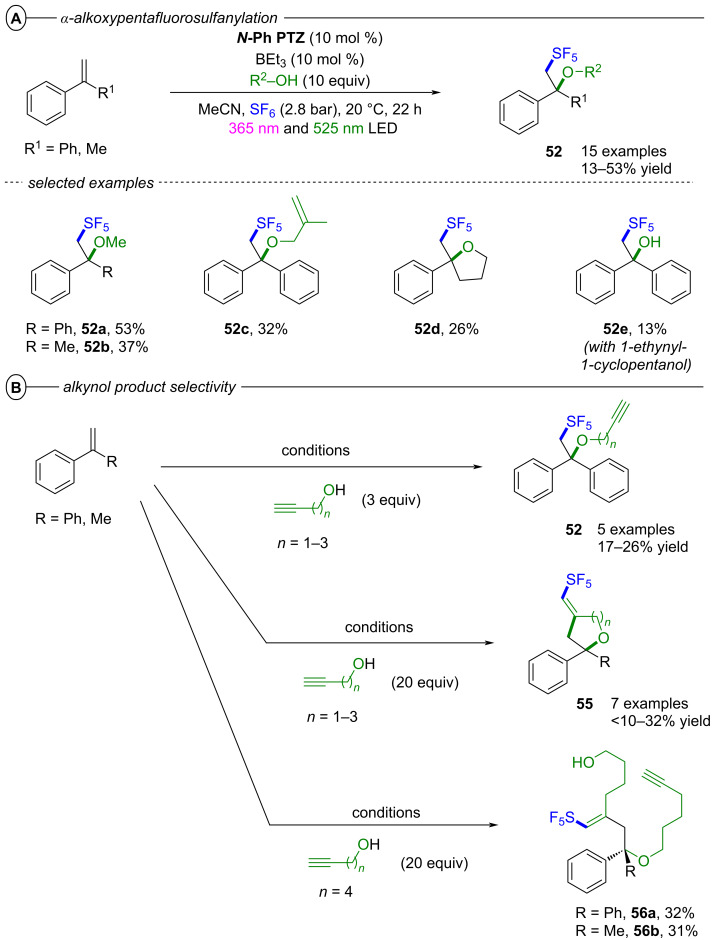
A) α-Alkoxypentafluorosulfanylation (top) and selected examples from the substrate scope (bottom). B) Effect of chain length on alkynol product selectivity.

Following their initial publication, the Wagenknecht group expanded their scope to the α-alkoxypentafluorosulfanylation of styrenes by addition of alcohols [131]. Herein, addition of the Lewis acid BEt_3_ successfully suppressed the competing fluoride addition by complexing fluoride ions generated by oxidative quenching of *N-Ph PTZ and enabled trapping of cation **50^+^** instead by the alcohol. Acyclic and cyclic aliphatic alcohols as well as alcohols bearing alkene, alkyne and nitrile functionalities were successfully applied and gave α-alkoxypentafluorosulfanylated products **52** in poor to moderate yields (13–53%) (Figure 31A). Intramolecular addition of an alcoholic chain allowed for the generation of spiroethers (**52d**). Phenols and water were not tolerated, however, reaction of tertiary alcohols (such as 1-ethynyl-1-cyclopentanol) led to the formation of free alcohols (e.g., **52e** in 13% yield). When investigating the effect of different concentrations of the alcohol nucleophiles, the authors observed that higher concentrations of terminal alkynols suppressed formation of the open-chain product **52** and instead promoted formation of oxaheterocyclic compounds **55** [133]. Depending on the chain length of the alkynol, oxepans, tetrahydropyrans and furans were obtained in <10–32% yields (Figure 31B).

Mechanistic investigations suggested that the alkynol is deprotonated by fluoride ions in the higher concentration regime and **49^•+^** is trapped by the alkoxide before it can react with ^•^SF_5_ (Figure 30B). The oxaheterocyclic compounds **55** were obtained exclusively in their *E*-configurations which the authors tentatively attributed to kinetic differences in product formation as DFT calculations show only a minimal free energy difference of 0.1 kcal mol^−1^ between ***Z*-54^•^** and ***E*-54^•^** (Figure 30B). Longer chain lengths of the alkynol, as in 5-hexyn-1-ol, did not afford oxaheterocyclic products but instead doubly substituted open-chain products **56a** and **56b** (Figure 31B).

Although overall the yields of fluoropentafluorosulfonylation and alkoxypentafluorosulfanylation reactions were lacking, these must be viewed in the context of the extraordinary inertness of SF_6_ towards chemical reactions [132] and thus represent a remarkable synthetic achievement.

**Figure 32:**
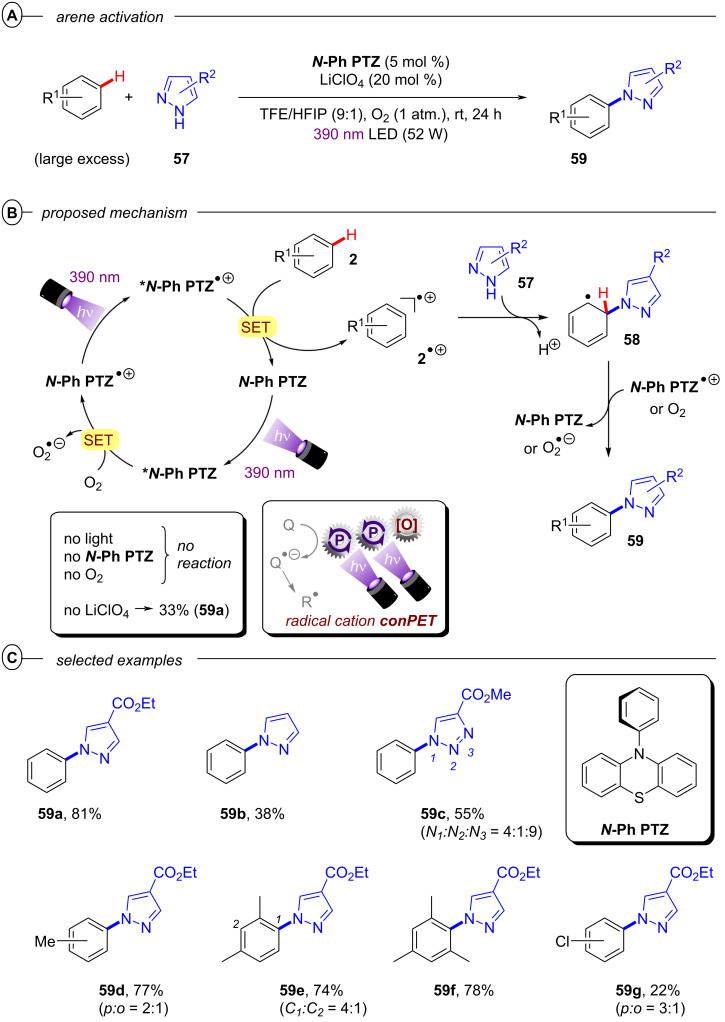
A) Oxidative amination of arenes with azoles catalyzed by ***N*-Ph PTZ**. B) Selected examples from the substrate scope. B) Proposed mechanism.

**2.2.2 Arenea activation:** In 2021, Wickens and co-workers disclosed a conPET protocol for the oxidative azolation of moderately electron-rich and electron-neutral arenes by N-heterocyclic nucleophiles [134], representing a synthetic advancement from the engagement of electron-rich arenes seminally achieved by Nicewicz and co-workers via single-photon PRC [135]. ***N*-Ph PTZ** was employed as a photocatalyst, O_2_ as the terminal oxidant and fluorinated alcohols as co-solvents due to their stabilizing effects on radical cations (Figure 32A) [136–137]. The authors postulated that photoexcitation and subsequent SET oxidation of *N-Ph PTZ by O_2_ generates ***N*-Ph PTZ^•+^**. A second excitation process furnishes *N-Ph PTZ^•+^ which oxidizes the arene to its radical cation via SET (Figure 32B). The azole **57** then nucleophilically adds to the aryl radical cation, yielding (with the loss of a proton) the aryl radical **58**. Though the authors do not propose a detailed mechanism for the subsequent reaction it can be postulated that deprotonation of the addition product followed by a subsequent oxidation and deprotonation generate product **59**. Oxygen uptake experiments revealed a consumption of roughly 2.3 equiv of O_2_ based on the amount of product which indicates that this second oxidation step is either performed by another molecule of ***N*-Ph PTZ^•+^** or directly by O_2_. Addition of substoichiometric amounts of LiClO_4_ (or other Lewis acids) promoted product formation which the authors attributed to a suppression of back electron transfer between superoxide (O_2_^•^**^−^**) and ***N*-Ph PTZ^•+^** by facilitating the disproportionation of superoxide to peroxide and O_2_. Under these optimized conditions, benzene was successfully coupled to several pyrazoles bearing electron-withdrawing and electron-donating functionalities as well as triazoles (**59c**) in poor to high (22–88%) yields (Figure 32C). Methyl-substituted arenes (toluene, *m*-xylene and mesitylene) proceeded efficiently under the reaction conditions (**59d**–**f**). Electron-poor chlorobenzene was oxidized in low (22%) yield (**59g**), representing an upper redox limitation of the system.

An aspect overlooked in all three aforementioned reports proposing *N-Ph PTZ^•+^ as a super photooxidant is its ultrashort excited state lifetime – ≥36 ps as reported by Wasielewski [128] – that renders diffusion-controlled photochemistry implausible. Wickens and co-workers did not detect indications of preassembly in the steady-state UV–vis [134], but it cannot be ruled out (discussion vide supra, section 2.1.4). Since Wickens and co-workers employed a vast excess of arenes in most reactions (up to ≈8 mL as solvent, i.e., 5.6 M PhH w.r.t. ≈ 1.3 mM of ***N*-Ph PTZ**), the statistical probability of an arene molecule in close proximity to the excited state upon its formation is high enough for static quenching [40,53] such that there may be no need to invoke an ‘organized’ [40,53]. However, lower excesses (5–10 equiv) of more electron-rich alkylarenes were successful, and in Wagenknecht and co-workers’ report styrenes were employed as the limiting agent (0.1 M styrene w.r.t. 5.0 mM ***N*-Ph PTZ**). Therefore, an organized preassembly is likely at these lower substrate concentration regimes, which we propose involved π–π stacking interactions based on Barham’s results and Hauer’s transient absorption spectroscopic investigations of analogous triarylamine radical cations (vide infra, Figure 37 and following discussion).

**2.2.3 C(sp^3^)–H activation:** Apart from previously mentioned applications of conPET for the generation of super-reductants or super-oxidants for SET reduction or oxidation of substrates, it can also provide access to other transformations. Recently, the generation of chlorine radicals as powerful HAT agents has been a topic of increased interest and the challenging generation of Cl^•^ has been achieved by monophotonic PRC oxidation of Cl^−^ to Cl^•^ by highly oxidizing noble metal photocatalysts or **Mes-Acr^+^** [138–140]. Another noteworthy method for accessing this highly reactive HAT agent is the photoelectrochemical generation of Cl^•^ reported by Xu and co-workers (vide infra, Figure 56 in section 3.1.2 focused on Minisci-type processes) [141].

**Figure 33:**
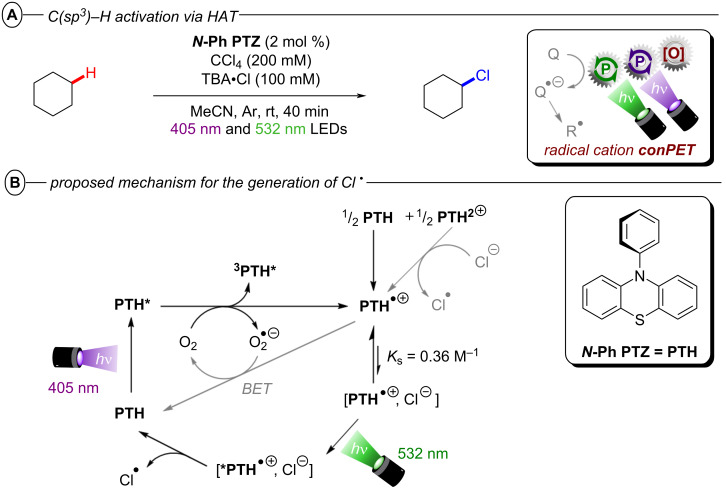
A) C(sp^3^)–H bond activation by HAT via chloride oxidation by *N-Ph PTZ^•+^. B) Proposed mechanism for the formation of Cl^•^ under single and multiple wavelength irradiation.

Meyer, Hu and co-workers recently reported how ***N*-Ph PTZ** could function as a conPET catalyst for the generation of Cl^•^, the latter serving as a HAT agent for the activation of unreactive C(sp^3^)–H bonds in hydrocarbons (Figure 33A) [142]. In their protocol, ***N*-Ph PTZ** is excited to *N-Ph PTZ by near-UV LED irradiation (390 nm or 405 nm) and this excited state undergoes oxidative quenching by O_2_ or CCl_4_ to afford ***N*-Ph PTZ^•+^** (Figure 33B). This radical cation can either disproportionate into ***N*-Ph PTZ** and ***N*-Ph PTZ^2+^** or be photoexcited to *N-Ph PTZ^•+^ by green LED irradiation (532 nm). While oxidation of Cl^−^ to Cl^•^ (*E*^p^_ox_ = +1.46 V vs NHE in MeCN) by *N-Ph PTZ or ***N*-Ph PTZ^•+^** (*E*_1/2_ = +0.92 V vs NHE) is thermodynamically out of reach, both ***N*-Ph PTZ^2+^** (*E*_1/2_ = +1.59 V vs NHE) and *N-Ph PTZ^•+^ (*E_1/2_ = +2.31 V vs NHE) possess sufficient oxidative redox power to generate Cl^•^. Experiments were conducted under single wavelength (405 nm) and dual wavelength (405 nm and 532 nm) irradiation with 1,1-diphenylethylene, cyclohexane, toluene, 1,4-dioxane and cycloheptane. Chlorinated products were generated under both conditions, however, yields were generally higher when dual wavelength irradiation was employed (increases of 85% for 1,1-diphenylethylene and 38% for cyclohexane). Since ***N*-Ph PTZ^•+^** barely absorbs near-UV light, the authors attributed the formation of chlorinated products under single wavelength irradiation to oxidation of Cl^−^ by the disproportionated species ***N*-Ph PTZ^2+^**. However, direct oxidation of Cl^−^ by *N-Ph PTZ^•+^ formed via less efficient excitation of ***N*-Ph PTZ^•+^** under exclusive irradiation at 405 nm cannot be ruled out. In this case, the increased yield under two wavelength irradiation could simply be attributed to a more efficient formation of highly oxidizing *N-Ph PTZ^•+^. Due to the aforementioned ultrashort excited state lifetime of *N-Ph PTZ^•+^, a preassembly of ***N*-Ph PTZ^•+^** with Cl^−^ is required for efficient oxidation and a Coulombic ion pair is the logical proposal for this.

While the work of Meyer and Hu elegantly demonstrates additional fields of application for highly oxidizing conPET catalysts, their work so far only outlines the conceptual idea. Xu and co-workers’ success with photoelectrochemical oxidation of HCl for activation of unreactive C(sp^3^)–H bonds demonstrates clear advantages including the absence of any photocatalyst and requirement of only one photon per product molecule (vide infra).

### 3 Photoelectrochemistry in organic synthesis

#### 3.1 Oxidative activation

**Figure 34:**
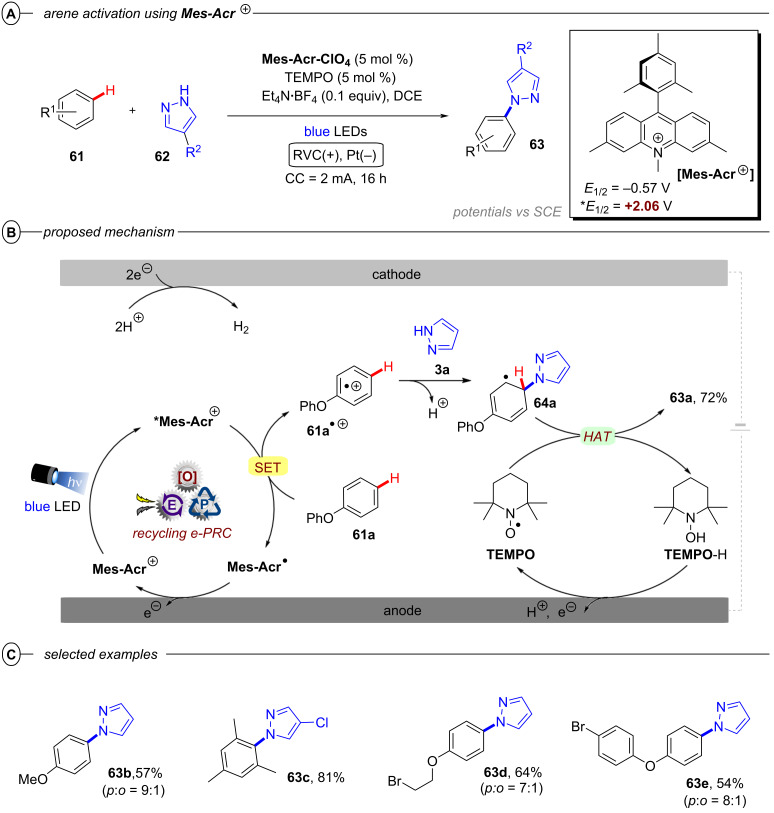
A) Recycling e-PRC C–H azolation of electron-rich arenes with pyrazoles using **Mes-Acr^+^** as a photocatalyst. B) Proposed mechanism. C) Selected examples from the substrate scope.

**3.1.1 Arene activation: *C(sp^2^)–H activation:*** In the oxidative direction, e-PRC has demonstrated impressive synthetic advancements in C(sp^2^)–N and C(sp^2^)–O bond formations from arene or alkene substrates. Since such photoredox reactions typically rely on SET oxidation of the unsaturated system, they tend to be carried out with relatively electron-rich substrates due to their lower oxidation potentials that are accessible under (unassisted) monophotonic PRC by closed-shell excited states. In this regard, Xu and Hou disclosed a ‘recycling e-PRC’ azolation of electron-rich arenes (generally referred to as **60** in this section) with pyrazoles **62** employing 9-mesityl-10-methylacridinium perchlorate **Mes-Acr-ClO_4_** as photocatalyst (Figure 34A) [143]. In the mechanism for the reaction of oxydibenzene **61a** and pyrazole **62a** (Figure 34B), **Mes-Acr^+^** (*E*_1/2_ = −0.57 V vs SCE) is photoexcited, generating the highly oxidizing state *Mes-Acr^+^ (*E_1/2_ = 2.06 V vs SCE). The latter oxidizes **61a** to arene radical cation **61a^•+^**, generating **Mes-Acr^•^** at the same time, which is then oxidized at the anode to regenerate the active catalyst. The radical cation intermediate is trapped with pyrazole to yield the radical **64a**, which reacts with 2,2,6,6-tetramethylpiperidinyloxy (**TEMPO**) in a HAT reaction to generate the target coupling product **63a** in 72% yield and **TEMPO**-H, a species that can also be oxidized at the anode.

With the optimized conditions in hand, the authors investigated a variety of arenes, obtaining products like **63b**–**e** in good to high yields (57–81%, Figure 34C). Interestingly, 1-bromo-4-phenoxybenzene reacted selectively only at the phenyl ring, affording **63e** with the *para*-product as the major regioisomer. Compared to the seminal work of Nicewicz [135] under monophotonic PRC that relies on molecular O_2_ for catalyst turnover, Xu and co-workers’ electrochemical catalyst turnover furnishes a PEC protocol with better prospects for scale up. Moreover, compared to the preceding ‘radical ion’ photocatalytic protocols of Lambert and co-workers (e-PRC, vide infra), Barham and co-workers (e-PRC, vide infra) and Wickens (conPET, vide supra), Xu and co-workers showed a key advantage of the recycling e-PRC approach that allows arene to be used as the limiting reactant rather than a (vast) stoichiometric excess.

**Figure 35:**
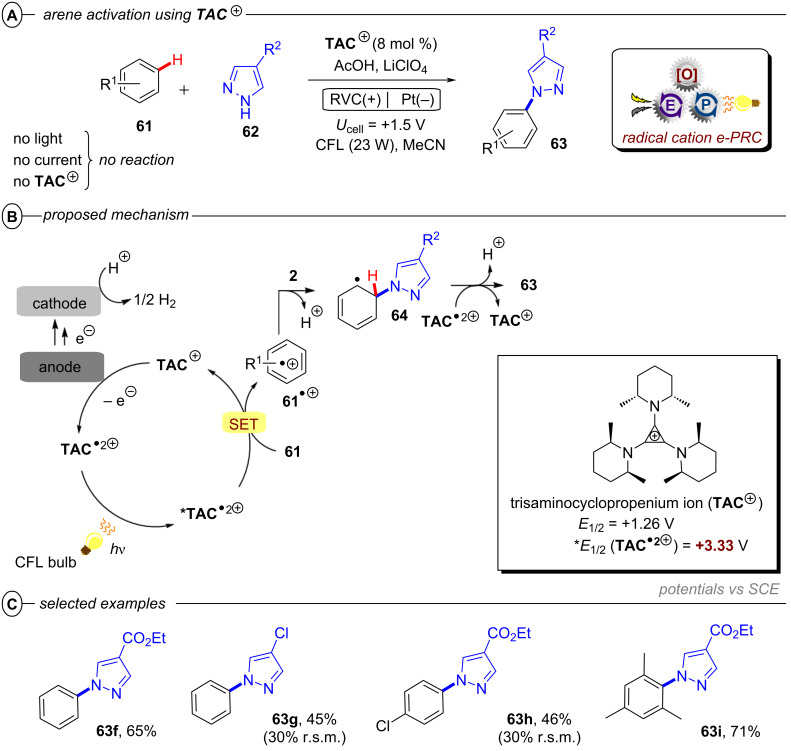
A) Radical ion e-PRC direct oxidation of unactivated arenes using **TAC^+^** as an electro-activated photocatalyst. B) Proposed mechanism. C) Selected examples from the substrate scope.

However, as per the groundbreaking report of Nicewicz [135], the limitation of oxidative power remains that allowed only electron-rich arenes to be engaged. In recent years it became desirable to target oxidation of electron-neutral and electron-deficient arenes (e.g., those containing halogen atoms or benzene itself, *E*^p^_ox_ = +2.48 → >+3.0 V vs SCE), since these are cheaper chemical feedstocks. In this respect, in 2019 the Lambert group reported a trisaminocyclopropenium cation **TAC^+^** [144–147] as an e-PRC catalyst for the oxidation of unactivated arenes and their coupling with nitrogen heteroaromatics (Figure 35A) [148]. Mechanistically, the electrochemical oxidation of the colorless **TAC^+^** cation (*E*_1/2_ = +1.26 V vs SCE) generates the colored dication radical **TAC^•2+^** (Figure 35B) [145–147,149]. Subsequent photoexcitation affords *TAC^•2+^ (*E_1/2_ = +3.33 V vs SCE) as a super oxidant [150], which can oxidize target arene **1** via SET to its aryl radical cation **61^•+^** with concomitant regeneration of **TAC^+^**. At this point, the mechanism for **61^•+^** follows a similar pathway described in the conPET oxidative arene activations (vide supra, section 2.2.2, Figure 32C). Proton reduction (HER) was proposed as the corresponding cathodic half-reaction, since gas bubbles were observed and since an excess of AcOH was necessary for reaction efficiency. The reaction tolerated benzene (**63f**) and even aryl chlorides to give products **63g** and **63h**, albeit in modest yields (Figure 35C). This method is also ‘backwards compatible’ with easily oxidized substrates such as mesitylene and other alkylated benzenes (e.g., forming **63i**).

**Figure 36:**
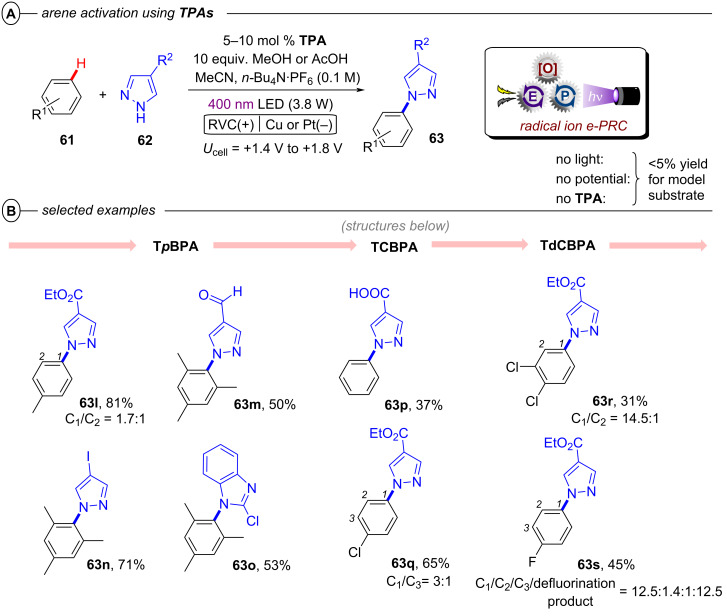
A) Radical ion e-PRC direct oxidation of unactivated arenes using **TPA** as an electro-activated photocatalyst. B) Selected examples from the substrate scope.

In the context of e-PRC C(sp^2^)–N bond formations, the Barham group unveiled tri(*p*-substituted)arylamine (**TPA**) radical cations [151–154] as a tunable class of radical ion e-PRCat (Figure 36A) [155]. The most interesting feature of **TPAs** is that modifying their substituents in *para*-position to the triphenylamine core allows facile tuning of the oxidative power of their radical cationic photocatalyst forms [54,156].

**Figure 37:**
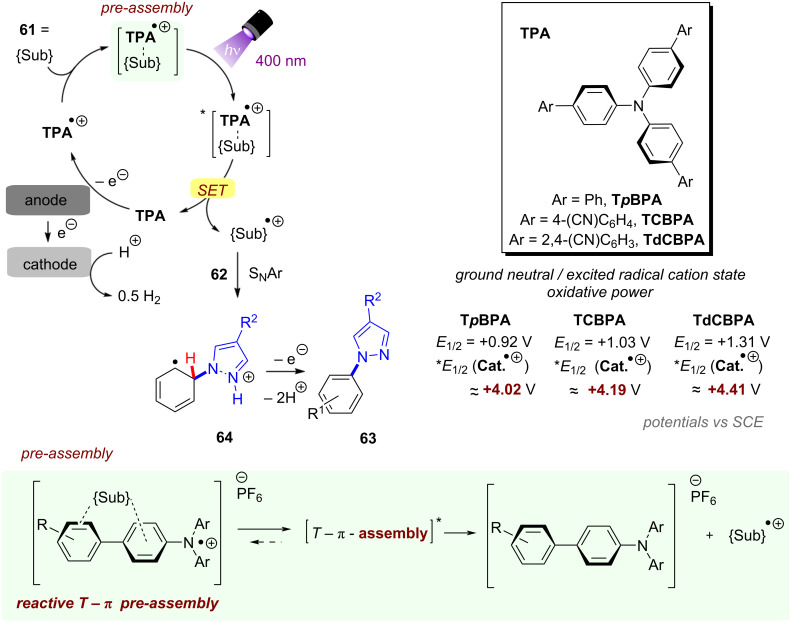
Proposed mechanism (top) and mode of preassembly (bottom).

In fact, while the use of a moderately-powerful **TPA** (**T*p*BPA**) enabled the C(sp^2^)–H azolations of alkylbenzenes in high yields and chemoselectivity (**63l**–**o** in Figure 36B), the substitution of its *para*-phenyl group with a *para*-(4-)benzonitrile group resulted in a much more powerful **TCPBA**, that allowed C–H amination of benzene and chlorobenzene in good yields (**63p**–**q**). Finally, the most potent **TPA** (**TdCBPA**, with a record breaking *E_1/2_ = +4.41 V vs SCE) allowed SET oxidations even of dichlorobenzenes and fluorobenzene (**63r**–**s**). In the mechanism, electrochemical oxidation of **TPA** generates the radical cation **TPA^•+^** as the active photocatalyst. The authors demonstrated for the first time the crucial importance of a radical ion photocatalyst-substrate preassembly, necessary to achieve the reactivity of the picosecond-lived photoexcited radical ions like *TPA^•+^ (4.6 ps for **T*p*BPA^•+^** and 8.6 ps for **TCBPA^•+^**, respectively) in competition with their photophysical deactivation (Figure 37). Subsequent photoexcitation of **[TPA^•+^--- Sub]** then leads to the reactive assembly *[TPA^•+^--- Sub], which, upon inner-sphere SET, generates the arene radical cation **Sub^•+^** and regenerates **TPA**. Finally, **Sub^•+^** is intercepted by the N-heterocyclic nucleophile **62** followed by loss of protons and further SET (to the anode or to **TPA^•+^**) to obtain the azolated arene product **63**.

The detailed mechanistic study on preassembly was easily accessible owing to the excellent stability of **TPA^•+^**s, which can be easily chemically generated, isolated and stored in their solid state without special precautions [153–154]. Assemblies **[TPA^•+^--- Sub]** were identified spectroscopically via changes in the UV–vis spectra and EPR spectra of isolated **TPA^•+^**s when mixed with arene substrates in excesses representative of the synthetic reaction.

**Figure 38:**
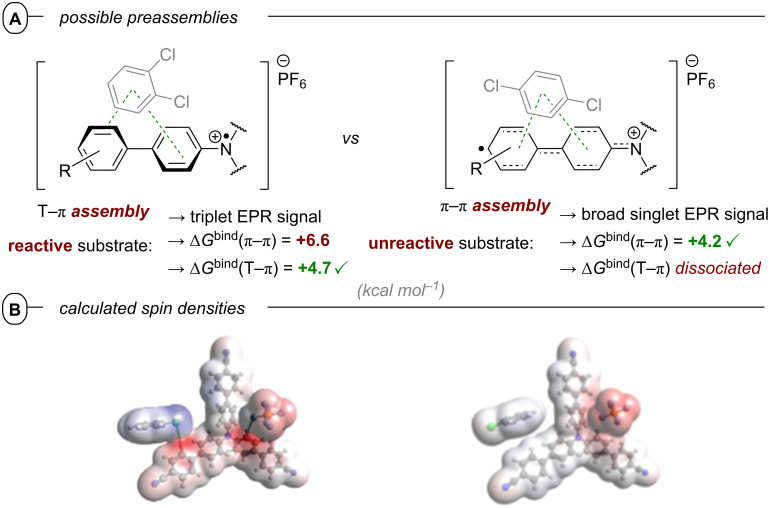
A) Possible preassemblies of reactive (left) vs unreactive (right) arenes. B) Calculated spin densities of T–π preassemblies of **TCBPA^•+^** with chlorobenzene where the halogen faces “in” (left) or “out” (right). Adapted from [29] (© 2021 S. Wu et al. and *Angewandte Chemie International Edition*, published by Wiley-VCH GmbH, distributed under the terms of the Creative Commons Attribution 4.0 International License, https://creativecommons.org/licenses/by/4.0).

The most noticeable comparison was observed when **TCBPA^•+^** was exposed to either 1,2- or 1,4 dichlorobenzene. The addition of the former “reactive” substrate caused a disruption in the electron paramagnetic resonance spectroscopy (EPR) signal, enhancing a distinctive triplet pattern. This evidence confirmed the need to localize spin density on the nitrogen atom within the initial preassembly in order to achieve reactivity. On the other hand, when the latter “unreactive” substrate was introduced, the signal was altered to a broad singlet, corroborating a different orientation of preassembly where the spin density was spread out from the N atom. The latter configuration stabilized the radical cation and decreased its oxidative power in the excited state for SET. DFT calculations (ωB97X-D or uB3LYP functionals) were also performed, and optimized structures were found involving T–π or π–π interactions (Figure 38A). It was also possible to evidence a preferred preassembly geometry for unsymmetrical compounds (PhX). In particular, for PhCl, the calculated spin density changed when the Cl atom faced "*in*" but did not change when facing "*out*" (Figure 38B), and thus the former orientation – accorded with spectral changes in the steady-state EPR and UV–vis – was proposed as favored.

The Barham group then concluded how, despite the strong absorption of **TPA^•+^**s in the near-IR visible region (approx. 600–900 nm, a D_0_ → D_1_ transition), only shorter wavelength excitations (400 nm) gave reactivity. This was later confirmed by the Hauer group using transient absorption experiments which revealed quenching only of the higher excited state (D_n_) of **T*p*BPA^•^**^+^ by mesitylene [157]. This is particularly important because for that catalyst/substrate combination no changes in the steady-state UV–vis spectrum were observed by Barham and co-workers [155]. These observations confirm how preassembly can serve as a general platform for anti-Kasha photochemistry, that temporally enables the participation of higher-order excited states participating in SET [69,120,157–160].

**Figure 39:**
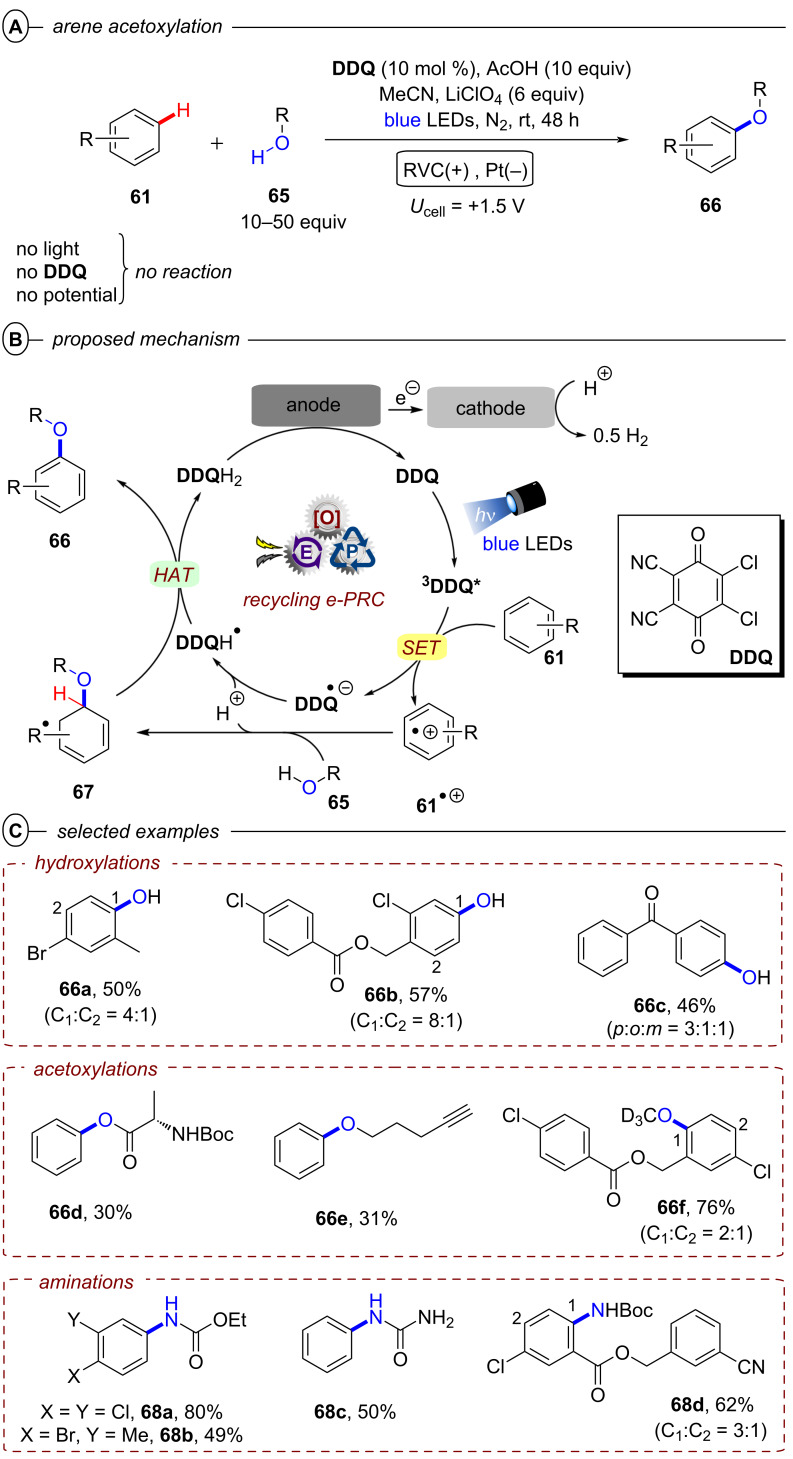
A) Recycling e-PRC C(sp^2^ )–H acetoxylation of arenes using **DDQ** as a photocatalyst. B) Proposed catalytic cycle. C) Selected examples from the scope.

Other oxidative coupling protocols involving C–H heterofunctionalization of arenes can be found in the literature that involve the electrochemical turnover of a photocatalyst [28–29], forging C(sp^2^)–O and C(sp^2^)–N bonds under recycling e-PRC conditions. In this regard, Lambert and co-workers disclosed arene hydroxylation or acetoxylation using **DDQ** as photocatalyst and water/alcohols (**65**) as reactants (Figure 39A) [161]. The main difference from previous works using this catalyst is that **DDQ** is regenerated by anodic oxidation of the reduced **DDQH_2_** – with concomitant cathodic reduction of protons to form hydrogen gas – completing the electrochemical reaction (Figure 39B). Thus, this e-PRC approach addresses and obviates one of the key aforementionated drawbacks of DDQ as used in photoredox-only transformations – the presence of a stoichiometric chemical oxidant such as *tert*-butyl nitrile as co-oxidant (vide supra, section 2.2). A plausible mechanism involves the photoexcitation of **DDQ**, which results in the generation of a highly oxidizing excited state *DDQ (*E_1/2_ = +3.18 V vs SCE). The excited state is subsequently engaged in a SET process with the arene species **61**, leading to the formation of two distinct species: the radical anion **DDQ^•−^** and the reactive radical cation **61^•+^**. At this stage, the alcohol molecule **65** acts as a trapping agent for the latter, leading to **67** after proton loss, that is, in turn, captured by **DDQ^•−^** to yield **DDQ**H**^•^**. The latter undergoes HAT from **67**, resulting in the formation of hydroquinone **DDQH_2_** and the desired functionalized product **66**.

**Figure 40:**
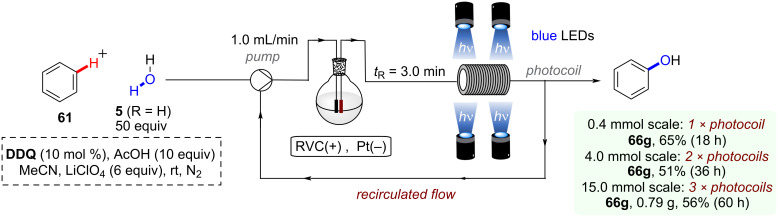
Gram scale hydroxylation of benzene in a recirculated flow setup.

The Lambert group also reported large-scale reactions. In particular, they successfully achieved the benzene-to-phenol (**66g**) hydroxylation reaction in a recirculated continuous flow setup, obtaining a 15 mmol scale reaction with 56% yield in 60 h with the use of three flow channels (Figure 40).

**Figure 41:**
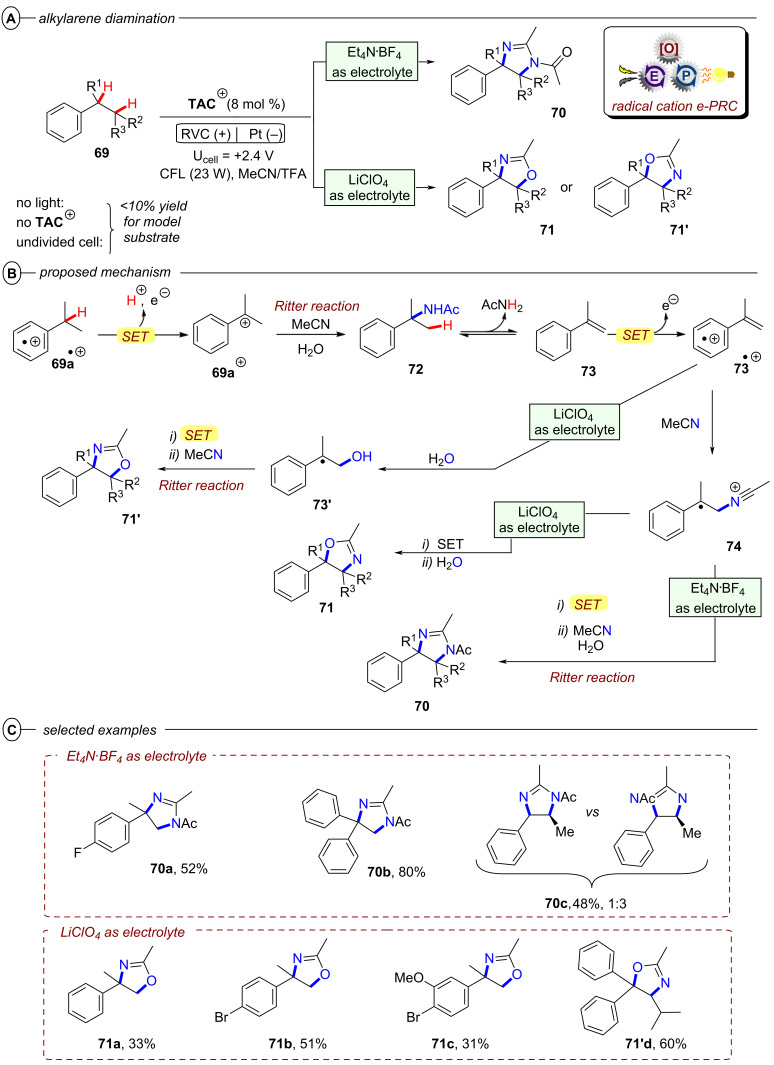
A) Radical ion e-PRC vicinal diamination of alkylarenes using **TAC^+^** as an electro-activated photocatalyst. B) Proposed catalytic cycle and electrolyte influence. C) Selected examples from the substrate scope.

***Benzylic C(sp^3^)–H activation*:** The Lambert group reported a C–H amination of alkylarenes **69** under radical ion e-PRC with **TAC^+^** to yield either dihydroimidazoles **70** or 2-oxazolines **71** and **71’**, depending on the electrolyte employed (Figure 41A) [162]. The authors proposed that the reaction starts with Ritter-type amination [163–164] of the substrate’s benzylic C–H bond. The photoexcited radical dication *TAC^•2+^ effects SET oxidation (vide supra, Figure 35B) of the substrate **69a** to its radical cation **69a^•+^** [165]. Deprotonation and subsequent oxidation of the latter leads to the cation **69^+^**, the solvolysis of which yields Ritter product **72**. At this point, according to the authors, acetamide **72** likely undergoes a reversible, acid-catalyzed elimination to yield α-methylstyrene **73** (Figure 41B) [166]. The subsequent solvent trapping and oxidation lead to the dihydroimidazole or oxazoline product. With these conditions in hand, dihydroimidazoles **70a**–**c** were obtained in useful yields using Et_4_N^.^PF_6_ as electrolyte. Interestingly, a simple change of electrolyte (Et_4_N∙BF_4_ → LiClO_4_) diverted reactivity toward oxazoline products **71a**–**c** and **71’d** was observed (Figure 41C). According to the authors, LiClO_4_ electrolyte modifies the stability of cationic intermediates and the addition of H_2_O to **73^•+^** or **74** affords **71’** and **71**, respectively (Figure 41B).

**Figure 42:**
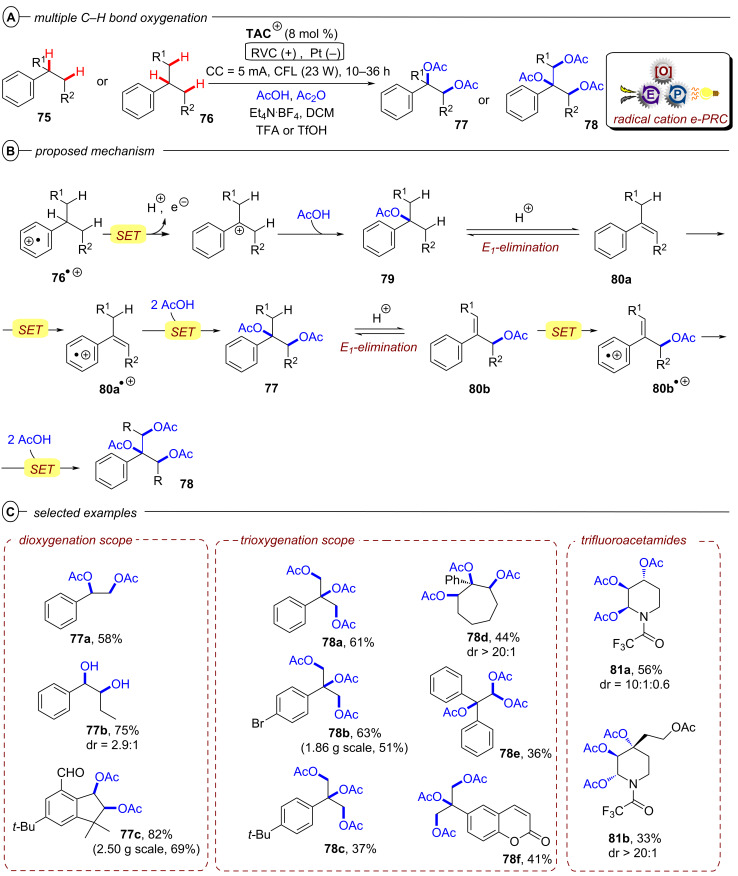
A) Sequential oxygenation of multiple adjacent C*–*H bonds under radical ion e-PRC using **TAC^+^** as an electro-activated photocatalyst. B) Proposed mechanism. C) Selected examples from the substrate scope.

Lambert and co-workers extended their radical ion e-PRC protocol toward oxygenation of multiple C–H bonds of alkylarenes simultaneously. This is an especially important target transformation, given the ubiquity of polyoxygenated molecules both in nature and in pharmaceutically active compounds [167–169]. At the same time, undesirable overoxidation reactions are highly likely. The control of oxidative chemoselectivity is also complex, although much progress has been made in the field of directed C–H oxidations [170–172]. The Lambert group found that treating alkylarenes **75** and **76** in the presence of **TAC^+^**, acetic acid, acetic anhydride and a strong acid like trifluoroacetic acid (TFA) or trifluoromethanesulfonic acid (TfOH) under PEC conditions led to dioxygenated or trioxygenated products **77** and **78** (Figure 42A) [173]. The reactions were carried out in an undivided cell under 5 mA constant current and irradiation by CFL light bulbs. In the mechanism, substrate **76** bearing an arene as a redox-active substituent undergoes SET oxidation by photoexcited radical dication *TAC^•2+^, leading to the radical cation **76^•+^**. Following deprotonation and further SET oxidation (by anode or by **TAC^•2+^**) monooxygenated intermediate **79** is generated. Under acidic conditions, **79** undergoes slow and reversible elimination (E_1_) to generate olefin **80a** (Figure 42B). Thanks to its conjugation with the arene moiety, **80a** can be further oxidized to form the dioxygenated product **77**. In support of this hypothesis, the reaction of **75** (R^1^ = OAc, R^2^ = Et) resulted in the formation of its corresponding dioxygenated product in 51% yield under the reaction conditions. Finally, if another C–H bond is present, another elimination/oxidation sequence occurs to yield the trioxygenated species **78** via **80b**. Regarding the reaction scope, the model reaction employed ethylbenzene and used TFA as a proton source, obtaining the product **77a** (Figure 42C). Then, a plethora of unbranched alkylarenes were examined, proving that in some cases a higher yield is obtained with a hydrolytic workup to generate a 1,2-diol like **77b** (the diastereomeric ratio favors the *anti-*isomer).

Once the efficiency of the dioxygenation protocol was established – even employing large scale reactions (2.5 g for **77c**) – the authors expanded the protocol to contiguous C–H trioxygenation within a single reaction flask, that was never reported before. Since E_1_-type elimination is the key step in the mechanism, branched substrates were generally employed because they are more prone to elimination processes than unbranched ones, and so more capable to be further oxidized after the initial dioxygenation. Products **78a**–**f** were obtained in modest to good yields (36–63%) using TfOH as the proton source and **78b** could be accessed on a gram scale (1.86 g) in a scale-up batch experiment. Finally, trifluoroacetamides were exploited as an alternative redox-active substituent. In particular, **81a** was obtained as a mixture of diasteroisomers among which single crystal X-ray analysis confirmed the *cis*-*trans* stereoisomer as the major one. Moreover, 4-alkylated piperidine derivatives were successfully employed to yield products like **81b**; when comparing to **81a** one can see how the diastereoselectivity was influenced by alkyl substitutents on the piperidine.

**Figure 43:**
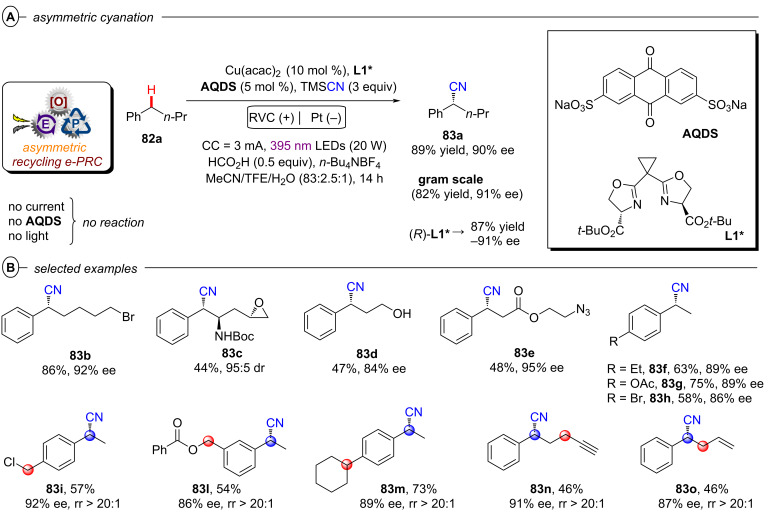
A) Enantioselective recycling e-PRC cyanation of benzylic C*–*H bonds using **ADQS** as photocatalyst. B) Selected examples from the substrate scope.

Recently, Xu and co-workers published the first example of an asymmetric synthetic PEC reaction, achieving the cyanation of benzylic C–H bonds by employing an additional chiral [Cu] catalyst, formed in situ from Cu(acac)_2_, TMSCN and a serine-derived bisoxazoline ligand L1* [174–175] (Figure 43A) [32]. Anthraquinone-2,6-disulfonate (**AQDS**) was used as a photocatalyst, where authors claimed the disulfonate groups promoted solubility, presumably by decreasing photocatalyst self-aggregation as found by the Barham group for a similar **DCA**-type system [176]. The PEC strategy described represents a key starting point for accessing chiral structures [174,177–180] via activation of benzylic C–H bonds in the absence of chemical oxidants or directing groups [181–183]. According to the authors, the high site selectivity derived from cleaving benzylic C–H bonds in a two-step sequential electron transfer/proton transfer mechanism instead of a single step HAT mechanism [184–187]. For the model substrate **82a**, the reaction with (R)-L1*, the enantiomer of L1*, was tested, resulting in a comparable result for the enantiomeric product. Also, a gram-scale process was evaluated, yielding **83a** in good yield and enantioselectivity (82% yield and 91% ee). With the optimized conditions in hand, a wide number of substrates were analyzed. Alkyl bromides (**83b**), epoxides (**83c**), alcohols (**83d**) and alkyl azides (**83e**) were all tolerated, resulting in the desired products in moderate to high yields (44–86% in Figure 43B). Once established that the method could be applied to substrates bearing different functional groups, Xu and co-workers shifted their focus to analyze arene electronic properties and substrates with multiple potential reactive C–H bonds. Regarding the first challenge, both electron-rich and electron-deficient alkylarenes were well-tolerated, as evidenced by the results presented in Figure 43B for **83f**–**h**. Turning to the site selectivity, a preference was found for cyanation of the most electron-rich benzylic position. This preference allowed the functionalization of stronger C(sp^3^)–H bonds, such as those in ethyl groups compared to the same secondary benzylic sites weakened with α-electron-withdrawing groups (Cl and acetyl groups in **83i**–**l**, respectively).

**Figure 44:**
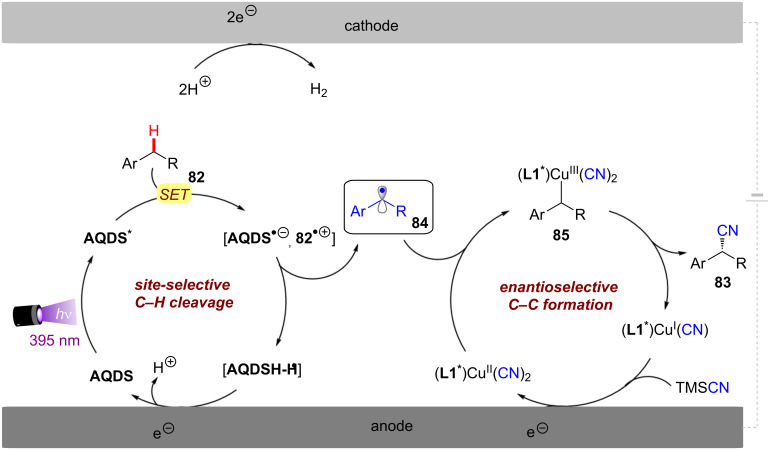
Proposed tandem mechanism by Xu and co-workers.

The reaction also displayed selectivity for the less hindered position, as exemplified by the absence of cyanation at the sterically more hindered tertiary benzylic carbon (**83m**) [188–191]. Furthermore, benzylic C(sp^3^)–H bonds were favored over their propargylic (**83n**) and allylic (**83o**) counterparts. The proposed mechanism for the enantioselective C(sp^3^)–H cyanation is composed of two relay catalytic cycles in tandem: a “site-selective C–H bond cleavage” cycle and an “enantioselective C–C bond formation” cycle (Figure 44). In the former cycle, photoexcitation of **AQDS** generates *ADQS as a potent photooxidant (*E_1/2_ = +2.00 V vs SCE) [192], which engages alkylarene **82** in SET to yield an radical ion pair [**AQDS^•^**^−^, **82^•^**^+^] [193–194]. Proton transfer between these species affords benzylic radical **84** and **AQDS**-**H**, where oxidation of the latter (by anode or by (L1*)CuI(CN)_2_) regenerates the catalyst and liberates a proton. The formation of the benzylic radical intermediate was confirmed by trapping experiments with an allyl sulfone, which gave a benzylic allylation product. At this point, the intermediate **84** is intercepted by the chiral copper complex (L1*)Cu^II^(CN)_2_ to generate a Cu^III^ species **85**, which undergoes enantioselective reductive elimination [185] to yield the benzylic nitrile **83** and the reduced catalyst (L1*)CuI(CN)_2_. The latter is promptly oxidized at the anode surface.

**Figure 45:**
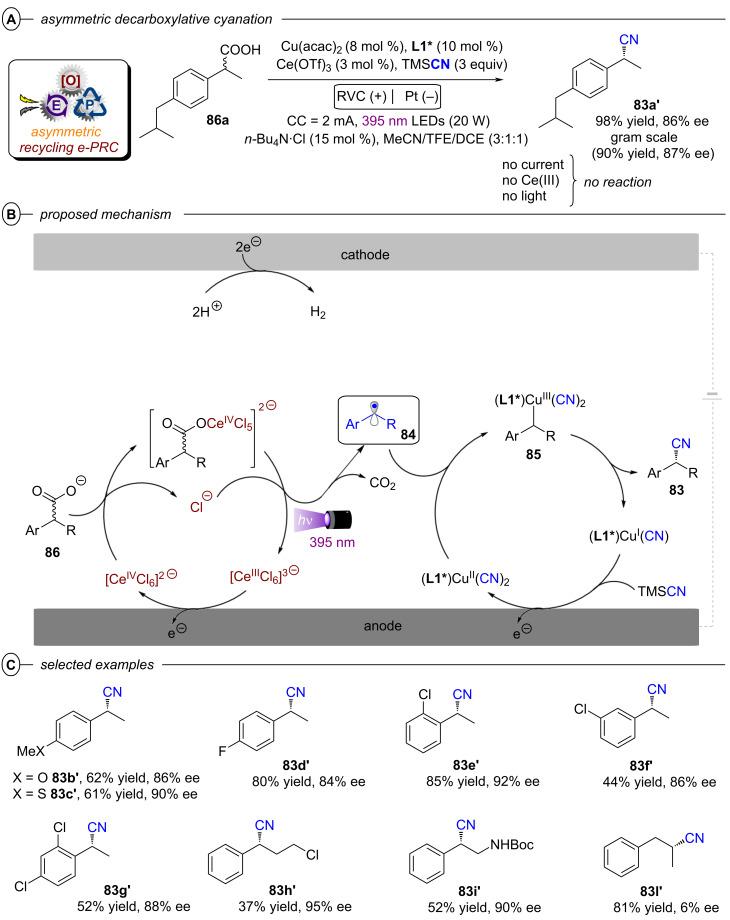
A) Enantioselective recycling e-PRC decarboxylative cyanation using Cu(acac)_2_, Ce(OTf)_3_ and a box ligand as catalysts. B) Proposed tandem mechanism. C) Selected examples from the substrate scope.

In an extension of this method, Xu and co-workers reported a PEC enantioselective decarboxylative cyanation using Ce(OTf) and Cu(acac)_2_ as catalysts and L1* as chiral ligand starting from benzylic carboxylic acid like **86a** (Figure 45A) [33]. In the mechanism, it is well known in the literature that cerium salts are converted to CeCl_6_^3^**^−^** in the presence of a chloride source (*n*-Bu_4_N∙Cl, in this case). The latter is oxidized at the anode to CeCl_6_^2^**^−^** (Figure 45B). Then, the coordination of the carboxylate **86** and the photoinduced LMCT process regenerates Ce(III) and leads to the benzylic radical **84**. Thereafter, the radical species then undergoes the same process for enantioselective cyanation proposed in Figure 44, to yield the reaction product **83**.

**Figure 46:**
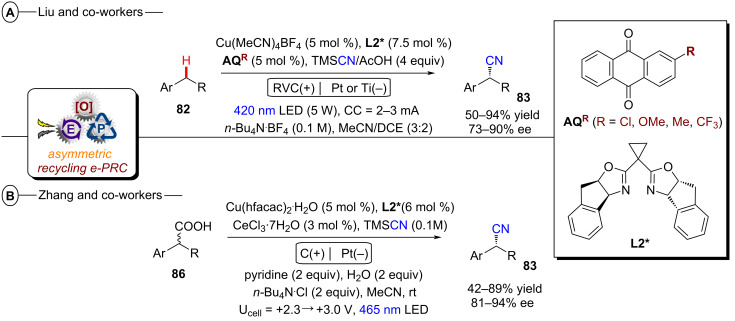
A) Enantioselective recycling e-PRC benzylic cyanation using Cu(MeCN)_4_BF_4_, box ligand and anthraquinone derivatives as catalysts. B) Enantioselective recycling e-PRC decarboxylative cyanation of benzylic carboxylic acids using Cu(hfacac)_2_, box ligand and CeCl_3_ as catalysts.

The scope of the PEC decarboxylation cyanation reaction was then explored with the optimized conditions. In particular, *para*-substituents of varying electronics at the aryl group such as OMe, SMe, and F were all tolerated (**83b’**–**d’**, Figure 45C). Good yields were obtained with *ortho*- and *meta*-substituted rings (**83e’**–**g’**) and also alkyl side chains with substituents (amide and chloride) were compatible (**83h’**,**i’**). A non-benzylic substrate was also tested, resulting in an efficient decarboxylative reaction but with no stereoselectivity (**83l’**). Finally, a gram scale reaction of the model substrate **86a** was conducted to obtain the product **83a’** in 90% yield and 87% ee*.* It is worth pointing out that an altogether highly similar PEC procedure for asymmetric cyanation has also been reported by Liu and co-workers (Figure 46A) [195]. The authors employed the bisoxazoline (Box) ligand L2* and Cu(CH_3_CN)_4_BF_4_ as the catalyst, under constant current conditions with reticulated vitreous carbon (RVC) as the anode and Pt/Ti as the cathode in an undivided cell. As photocatalyst, an array of anthraquinone-type photosensitizers **AQ^R^** were tested (R = Cl, OMe, Me and CF_3_), affording enantioselective cyanation products in good to excellent yields (50–94%) and ee (73–90%) values starting from both electron-rich and electron-poor (hetero)arenes.

Similarly, after the pioneering work of Xu, a closely-related decarboxylative cyanation protocol has been reported by Zhang and co-workers, who employed copper(II) hexafluoroacetylacetonate (Cu(hfacac)_2_), commercially inexpensive and earth-abundant CeCl_3_ and the box ligand L2* as catalysts (Figure 46B) [196].

***Aryl olefins activation*:** Among the many classical methods used to achieve olefin dioxygenations [197–199], those using transition metals, especially osmium, are undoubtedly the most widely used in organic synthesis [200–205]. However, the problems associated with the use of such transition metals, including issues of toxicity and expense, are widely known. For this reason, there has been a large interest to develop transition metal-free alternative methods that overcome the expense and toxicity of certain metal reagents or catalysts [206–211]. In this regard, electrochemistry has been illustrated as a sustainable synthetic alternative that does not require stoichiometric oxidizing agents [212–215]. Although oftentimes electrochemistry is technically not ‘transition metal-free’, the metal electrode is a heterogeneous surface that is easily separated post-reaction and reused.

**Figure 47:**
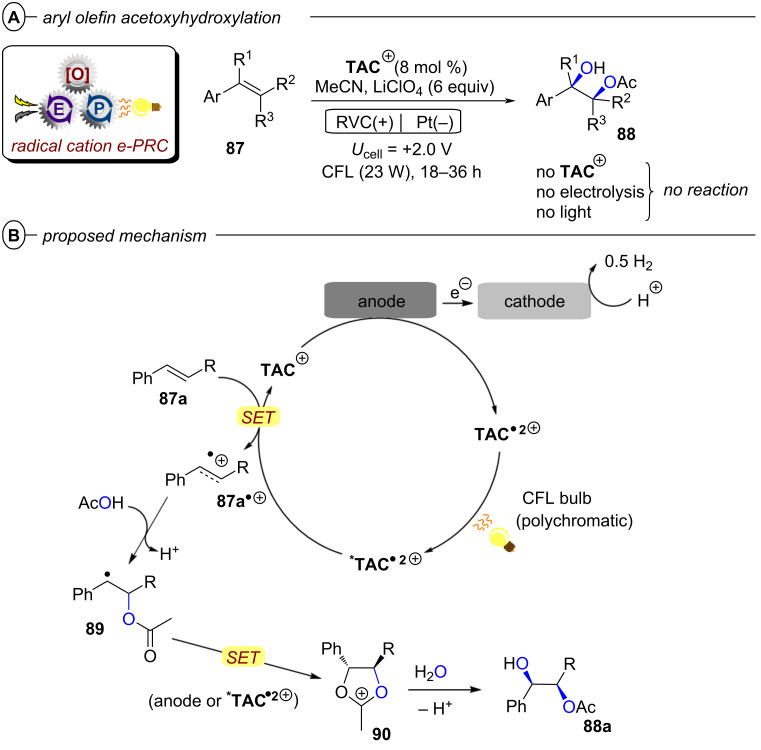
A) Radical ion e-PRC acetoxyhydroxylation of aryl olefins using **TAC^+^** as an electro-activated photocatalyst. B) Proposed mechanism.

**Figure 48:**
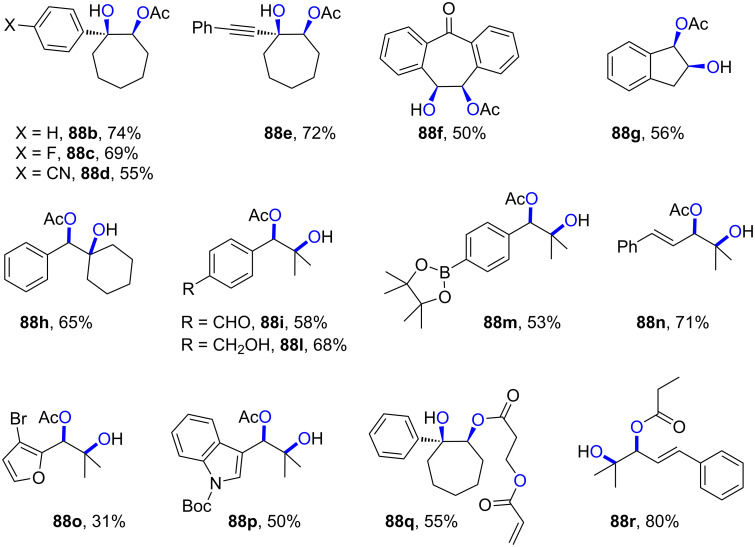
Selected examples from the substrate scope.

**Figure 49:**
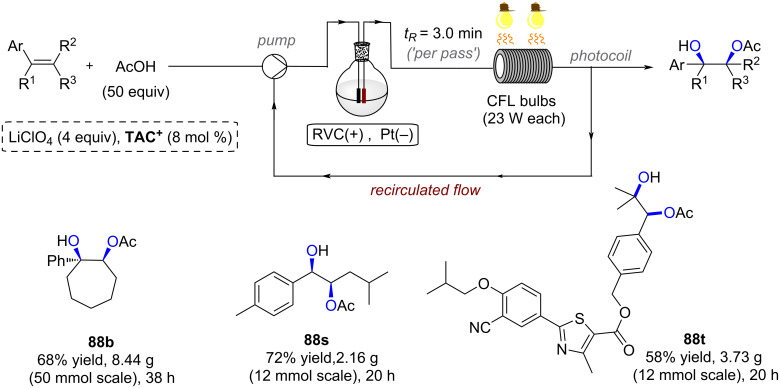
Photoelectrochemical acetoxyhydroxylation in a recirculated flow setup.

One of the main problems with electrolysis is that applied anodic potential can encourage overoxidation of olefins, leading even to undesirable oligomerization processes. This issue can be easily overcome with e-PRC, where the high requisite potential is generated transiently in the form of a photoexcited state in bulk solution. An initial demonstration of the potential of merging electrochemistry and photochemistry in this field is the acetoxyhydroxylation protocol for aromatic olefins reported by Lambert and Huang (Figure 47A) [216]. They used radical ion e-PRC employing **TAC^+^** as a catalyst to engage a number of activated olefins (benzylic or vinylic substituents), forming acetoxyhydroxylated products **88**. In the mechanism, olefin **87a** is oxidized by *TAC^•2+^ to its radical cation **87a^•+^**. Nucleophilic addition of AcOH to the latter yields benzylic radical **89** (Figure 47B), whose SET oxidation – either by anodic potential or by *TAC^•2+^ – induces an intramolecular cyclization to **90**. Hydrolysis of the cyclic intermediate releases the reaction product **88a**. The optimized conditions allowed hydroxyacetoxylation of different kind of cyclic alkenes with high levels of *syn-*diastereoselectivity (**88b**–**g**, Figure 48), demonstrating the method’s formidable tolerance to a range of electronically different substituents and even for conjugated systems such as **88e**. Benzylic groups, alcohols and aldehydes were all tolerated (**88h**–**l**). For open-chain substrates a boronic ester, a product-bearing styrene and different heterocycles including furan and indole were all well-tolerated in the syntheses of **88m**–**p**. In addition to the use of acetic acid to generate hydroxyacetate products, the authors tested other acids, obtaining the products **88q**,**r** in good to high yields (55–80%). Finally, scalability of the method was demonstrated up to multigram scales (up to 50 mmol) via a continuous flow approach. Multigram quantities of products **88b** (8.44 g), **88s** (2.16 g) and **88t** (3.73 g) were accessed using a recirculated flow setup depicted in Figure 49, in which the solution was circulated through Teflon tubes exposed to three CFL bulbs for 20–38 h with a residence time of 3 minutes ‘per pass’.

**Figure 50:**
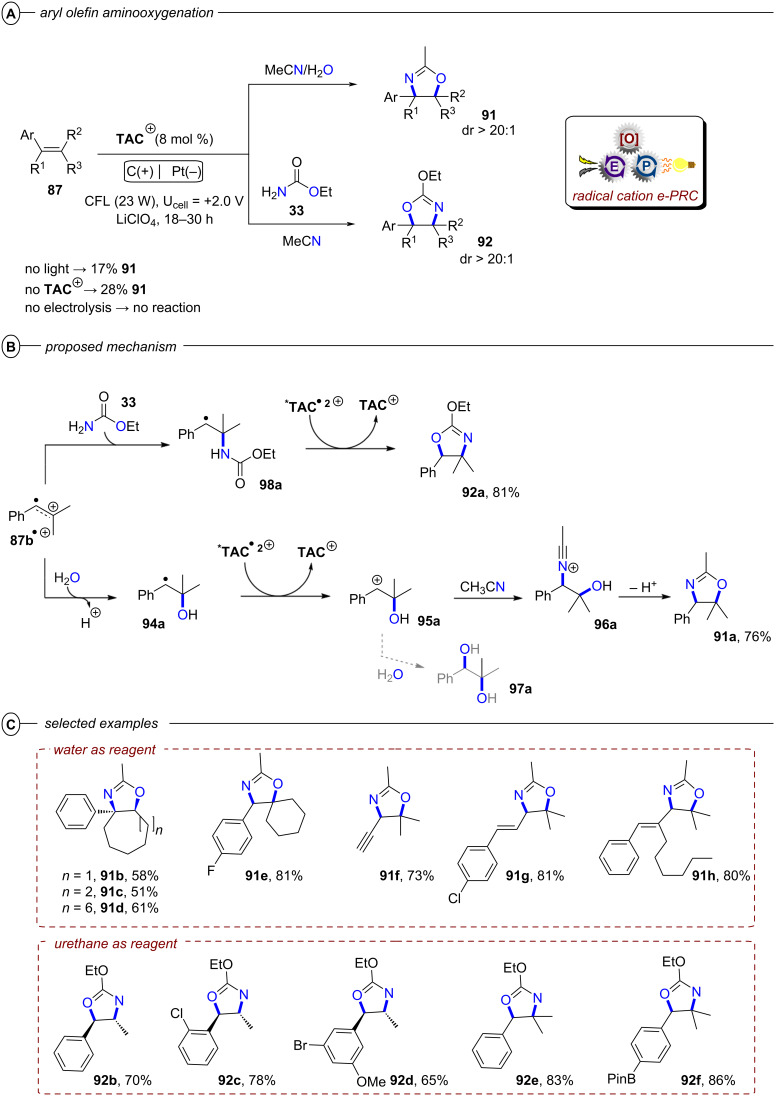
A) Radical ion e-PRC aminooxygenation of aryl olefins using **TAC^+^** as an electro-activated photocatalyst. B) Proposed mechanism for the synthesis of oxazolines and the influence of nucleophile. C) Selected examples from the scope.

Lambert’s group also described a method for the regiodivergent aminooxygenation of aryl olefins under similar PEC conditions with **TAC^+^** as catalyst (Figure 50A) [217]. This protocol forms aryl-substituted 1,2-aminoalcohols of both regioisomeric configurations **91** and **92** that are important architectural motifs in many complex molecules [218], including natural products [219–220], chiral auxiliaries and ligands [221]. In the proposed mechanism (Figure 50B), oxidative SET activation of alkene **87b** occurs by the electrochemically activated *TAC^•2+^. Radical cation **87b^•+^** can be trapped by water leading to the radical **94a**, whose SET oxidation (either by *TAC^•2+^ or the anode) and nucleophilic trapping by MeCN yields the intermediate **96a** through the intermediate **95a** (Figure 50C). Intramolecular addition of the hydroxyl group to the nitrilium ion leads to the oxazoline product **91a**. According to the authors, water is competitive as the second nucleophile, leading to formation of diol **97a**, particularly when present in large excess (dashed grey path in Figure 50B). On the other hand, the use of urethane **93** as nucleophile instead of water leads to the formation of radical **98a** via trapping of radical cation **87b^•+^**. Oxidation of **98a** leads to cyclization at the carbamate carbonyl oxygen, furnishing product **92a**. For the reaction with water as nucleophile, various cyclic and acyclic alkenes were found to undergo efficient aminooxygenations with high levels of *syn* diastereoselectivity (**91b**–**d,** Figure 50C) and in good yields (58–61%). Furthermore, reaction conditions were well-tolerated by a wide range of aryl olefins bearing different functional groups, such as fluorine and alkyne (**91e**,**f**). An intriguing example from the scope involves the utilization of 1,3-dienes as the starting material, which exclusively undergoes functionalization at the olefin position distant from the aromatic ring (**91g**,**h**). The authors then switched the focus to the regioisomeric products **92** using **93** as reactant. Again, a variety of 1,2-disubstituted and trisubstituted were tested (**92b**–**f**, Figure 50C), leading to the products in good to high yields (65–86%).

**Figure 51:**
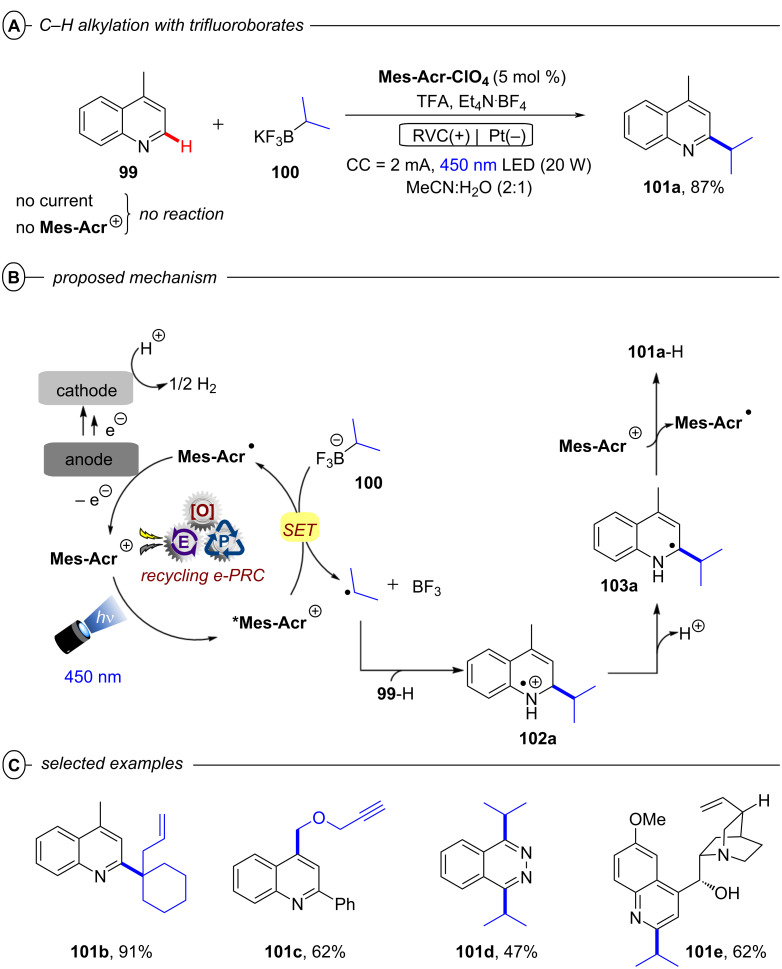
A) Recycling e-PRC C–H alkylation of heteroarenes with organic trifluoroborates using **Mes-Acr^+^** as photocatalyst. B) Proposed catalytic cycle. C) Selected examples from the substrate scope.

**3.1.2 Heteroarene activation:** The radical-mediated C–H Minisci-type [222] functionalization of heteroarenes like **99** enables rapid construction of 2-alkyl-substituted heteroaromatic building blocks [223], which are found in a variety of natural products, organic materials, small molecule drugs, and ligands [224]. Carbon-centered radicals can be generated under photoelectrochemical conditions from various precursors. In one of the seminal examples of contemporary synthetic PEC, Xu and co-workers used trifluoroborates under recycling PRC conditions with Fukuzumi’s catalyst [225] *Mes-Acr^+^ (Figure 51A) [226]. As aforementioned (vide supra, Figure 34B), irradiation of **Mes-Acr^+^** produces its highly oxidizing photoexcited state, *Mes-Acr^+^ (*E_1/2_ = +2.06 V vs SCE) [227–229]. SET between the latter and organotrifluoroborate **100** generates the acridinyl radical **Mes-Acr^•^** and an alkyl radical (Figure 51B) [230]. **Mes-Acr^•^** is then oxidized on the anode surface to regenerate **Mes-Acr^+^** in the typical recycling e-PRC manifold. The resulting alkyl radical adds to the protonated heteroarene **99**-H at the 2-position to give the radical cation **102a**, which is then deprotonated affording a C-radical intermediate **103a** [231]. A subsequent deprotonation affords the desired protonated product **101a**-H. A large range of heteroarenes were used as radical acceptors, including isoquinolines, phenanthridines, phthalazines, benzothiazoles acridines and purines (**41a**–**e**, Figure 51C). The conditions employed tolerated amine, alcohol, olefin and alkyne functional groups, in both the heterocyclic partner and radical precursor.

**Figure 52:**
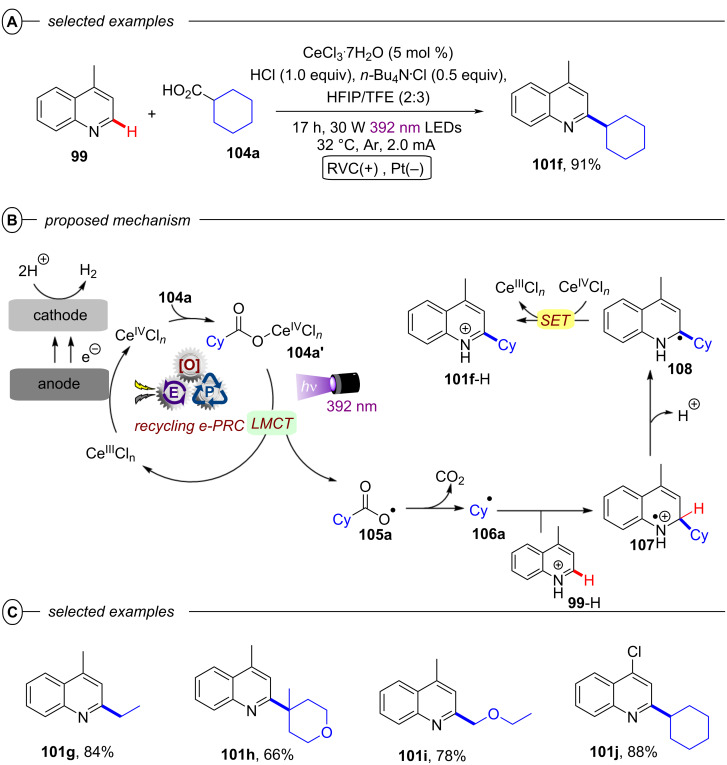
A) Recycling e-PRC decarboxylative C*–*H alkylation of heteroarenes using CeCl_3_·7H_2_O as catalyst. B) Proposed mechanism. C) Selected examples from the substrate scope.

Carbon-centered radicals can be also directly generated from carboxylic acids, which are inexpensive, stable and nontoxic feedstocks [89,232–236]. The carboxylate group upon oxidative SET evolves in the form of CO_2_, thus acting as a traceless leaving group. Xu and co-workers leveraged this by reporting a decarboxylative C–H alkylation of heteroarenes **99** under PEC conditions with CeCl_3_∙7H_2_O as catalyst (Figure 52A) [237]. The reaction begins with the anodic oxidation of the catalytic precursor Ce^III^ to Ce^IV^, followed by the coordination of the latter by the carboxylic acid **104a** to form complex **104a’** (Figure 52B).

Upon photoexcitation, **104a'** undergoes a ligand–metal charge transfer (LMCT) and cleavage to regenerate Ce^III^ and yield carboxyl radical **105a**, which decarboxylates leading to alkyl radical **106a** [238–239]. Addition of the resulting alkyl radical to protonated **99**-H at the 2- position affords radical cation **107**. This transient species loses a proton to give radical **108**, whose SET oxidation furnishes the protonated product **101f**-H. Several different examples of carboxylic acids were tolerated by the reaction conditions (primary, secondary, tertiary, α-alkoxy and aliphatic, **101f**–**j** in Figure 52C). A limitation is that the 4-position of the heteroarene must be blocked to prevent competing radical addition.

**Figure 53:**
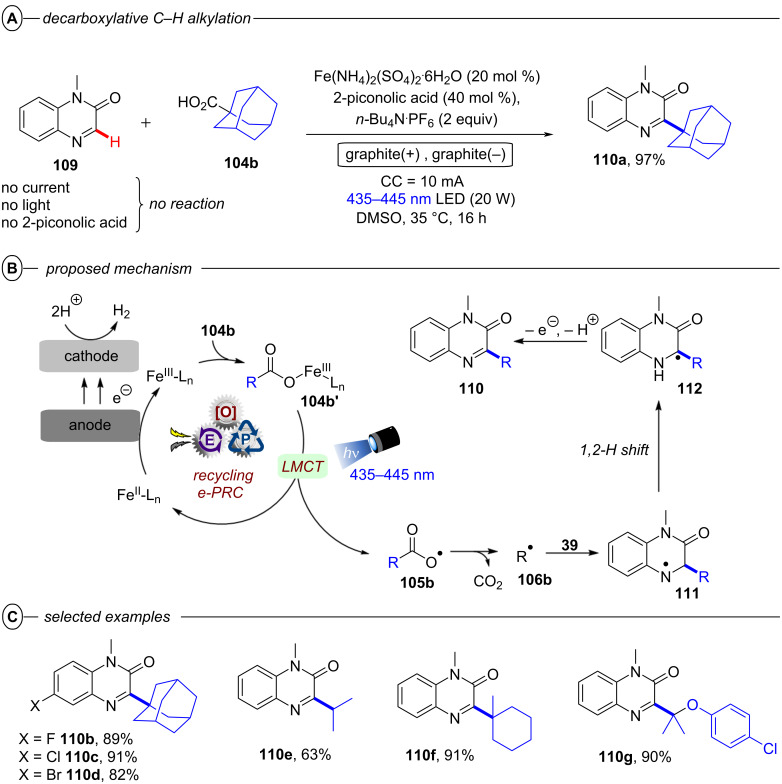
A) Recycling e-PRC decarboxylative C–H alkylation of heteroarenes using Fe(NH_4_)_2_(SO_4_)_2_·6H_2_O as catalyst. B) Proposed mechanism. C) Selected examples from the substrate scope.

A very similar protocol was reported by the Wang’s group [240], which employs a Fe^II^-based photoredox catalyst for decarboxylative C–H alkylation of quinoxalin-2(1*H*)-ones **109** (Figure 53A). The initial stages of the mechanism involve, as observed for cerium (vide supra), the generation of the photoactive Fe^III^−carboxylate complex **104b’** (Figure 53B) [99]. The photoinduced (435–445 nm) LMCT process leads to **105b**, the precursor of the reactive alkyl radical **106b** via decarboxylation. The C-centered radical was observed by HRMS via TEMPO or BHT radical trapping experiments when R = adamantyl. Coupling of this radical with **109** generates intermediate **111**, which via a 1,2-*H* shift yields **112**. Finally, anodic oxidation and deprotonation affords the reaction product **110**. Different radical acceptors with halogenated (F, Cl and Br) aromatic rings were well-tolerated under the reaction conditions, resulting in products **110b**–**d** in high yields (82–91%, Figure 53C). Both secondary and tertiary alkyl carboxylic acids led to desired products **110e**–**g** in good to excellent yields (63–91%).

The reports of Xu and co-workers as well as Wang and co-workers prove that LMCT is an effective, economical and eco-friendly strategy for homolytic cleavage of complexes of earth-abundant first-row and lanthanide metals such as Ni, Cu, Fe and Ce, to generate reactive radicals for couplings under PEC conditions [241], as well as under conPET conditions (vide supra, Figure 20, Fe-based catalyst).

**Figure 54:**
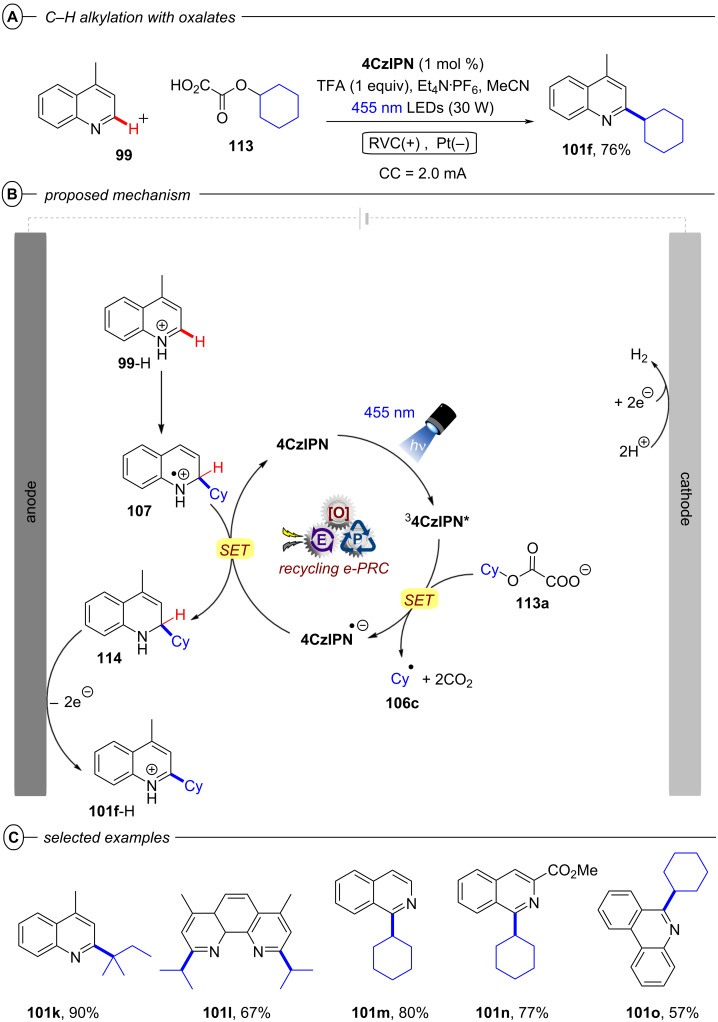
A) Recycling e-PRC C–H alkylation of heteroarenes with alkyl oxalates and **4CzIPN** as photocatalyst. B) Proposed mechanism. C) Selected examples from the substrate scope.

Xu and co-workers extended their previous alkylation method (vide supra, Figure 52) to alkyl oxalates **113** as precursors to alkyl radicals in the absence of any transition metal catalyst (Figure 54A) [242]. The alkyl oxalate **113a** reductively quenches the photoexcited catalyst *4CzlPN (*E_1/2_ = +1.35 V vs SCE) to afford **4CzIPN^•−^** and the alkyl radical **106c** after double decarboxylation (Figure 54B) [243]. Addition of the latter to the protonated heterocycle **99**-H produces radical cation **107**, which undergoes an SET reduction by **4CzIPN^•−^** to obtain 1,2-dihydroquinoline **114** with concomitant regeneration of catalyst. Lastly, **114** is SET oxidated at the anode to furnish product **101f**-H. While the reaction was suitable for various examples of secondary and tertiary oxalates (**101k**–**o**, Figure 54C), primary oxalates were ineffective alkyl radical precursors.

**Figure 55:**
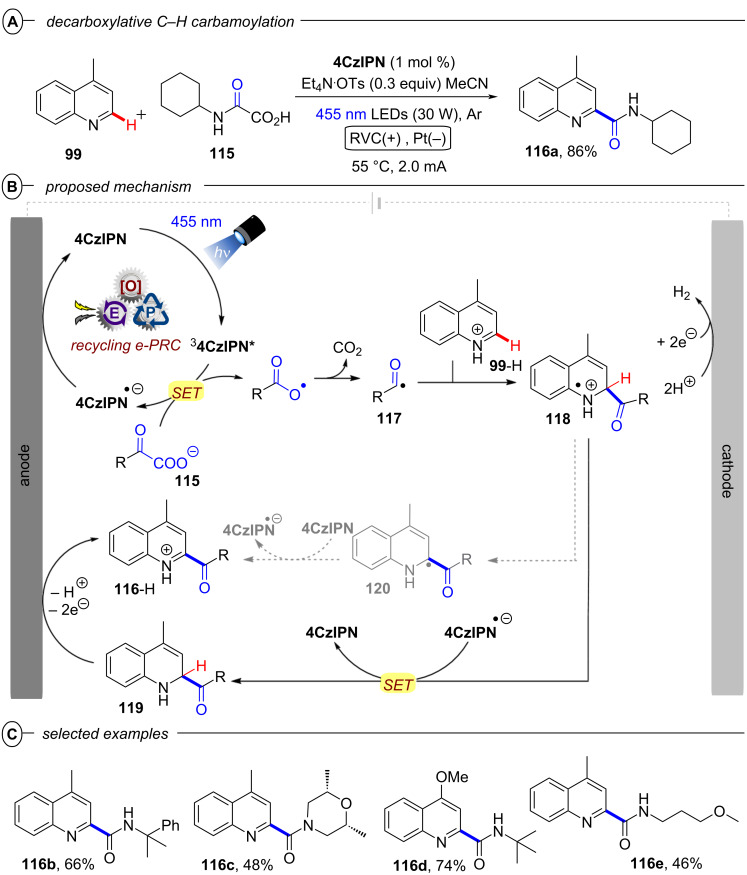
A) Recycling e-PRC decarboxylative C*–*H carbamoylation of heteroarenes using **4CzIPN** as photocatalyst. B) Proposed mechanism. C) Selected examples from the substrate scope.

Xu's group also deepened the application of decarboxylative radical formation in a PEC carbamoylation of heteroarenes using **4CzIPN** as photocatalyst (Figure 55A) [237]. The latter, upon photoexcitation (455 nm), oxidates the oxamate **115** in a SET process leading to the carbamoyl radical **117** through decarboxylation (Figure 55B). The reactive radical adds to protonated heteroarene **99**-H resulting in radical cation **118**, SET reduction of which (by **4CzIPN^•−^**, or by the cathode) affords **119**. The latter intermediate was detected in a HRMS experiment when R = NHCy. Finally, oxidation of dihydroquinoline **119** yielded the protonated product **56**-H. Alternatively, the authors proposed deprotonation of **118** affords radical **120** which could be oxidized by ground-state **4CzIPN** or by the anode (grey dashed mechanism). The substrate scope featured various examples of oxamic acids (bearing primary, secondary and tertiary *N*-substituents) and various electron-deficient N-heteroarenes (affording compounds such as **116b**–**e**) (Figure 55C).

**Figure 56:**
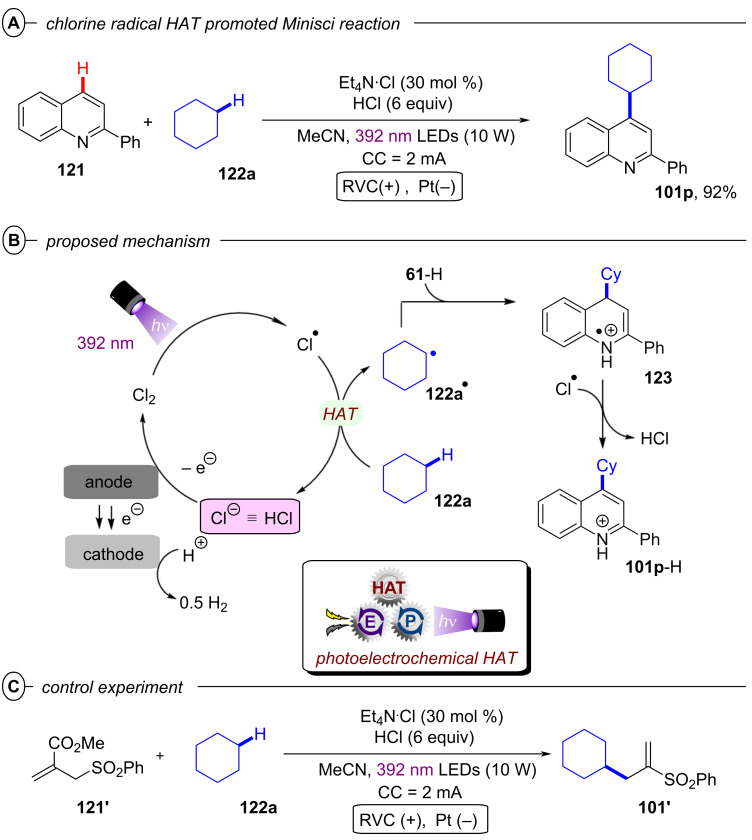
A) Photoelectrochemical HAT-mediated hydrocarbon activation via the chlorine radical. B) Proposed mechanism. C) Trapping experiment of the proposed radical with an allylic sulfone.

Another important vehicle to access Minisci reactive pathways is a direct C(sp^3^)–H bond activation via HAT, a method capable to generate radicals that are hard to obtain from photocatalytic SET or electrochemical transformations [244–246]. Conceptually relating to the conPET report of Meyer, Hu and co-workers (vide supra, Figure 33), the Xu group utilized PEC to access chlorine radicals (Cl^•^) under remarkably accessible reaction conditions, furnishing HAT agents to afford C(sp^2^)–H alkylations of heterocycles like 2-phenylquinoline **121** (Figure 56A) [141]. Anodic oxidation of chloride generates chlorine (Cl_2_). Subsequential light irradiation homolytically cleaves Cl_2_ (Figure 56B) and regulates a steady-state concentration of Cl^•^ [247]. Then, Cl^•^ engages unactivated C(sp^3^)–H bonds such as those of cyclohexane (**122a**), affording C-radicals (such as **122a^•^**).

The high bond dissociation enthalpy (BDE) of HCl (102 kcal mol^−1^) ensures that Cl^•^ can react with a plethora of activated and unactivated aliphatic C–H bonds [139–140,248–250]. The C-radical reacts with the heteroaromatic compound **121** to yield a radical cation intermediate **123**, which can then undergo rearomatization to furnish the protonated product **101p**-H. Corroborating the intermediacy of a cyclohexanyl radical, the authors conducted a control experiment using an allylic phenyl sulfone **121’** as reactant and detected the allylic radical substitution product **101’** (Figure 56C). Continuous generation of Cl_2_ by anodic oxidation and its photolysis avoids the direct use of toxic Cl_2_ gas and regulates a manageable low concentration at any given time [251].

**Figure 57:**
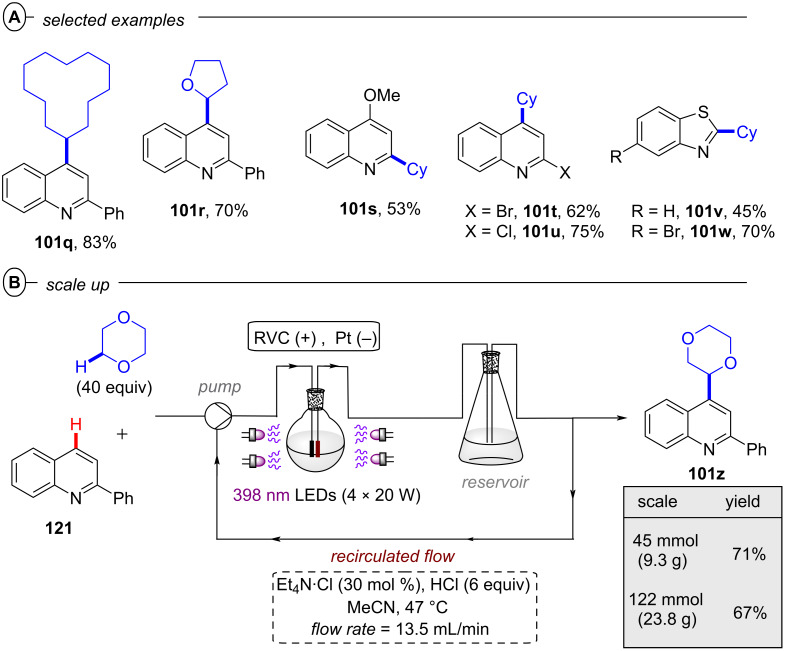
A) Selected examples from the substrate scope. B) Gram and decagram scale semi-continuous flow PEC HAT activation of 1,4-dioxane and its Minisci-type reaction.

The substrate scope was relatively broad with regard to both radical precursors and heteroarenes, tolerating many sensitive functional groups and affording products such as **101q**–**w** generally in moderate to high yields (45–83%) (Figure 57A). The authors achieved synthesis of **101z** on a gram and even decagram scale by employing a recirculated semi-continuous flow setup (Figure 57B).

**Figure 58:**
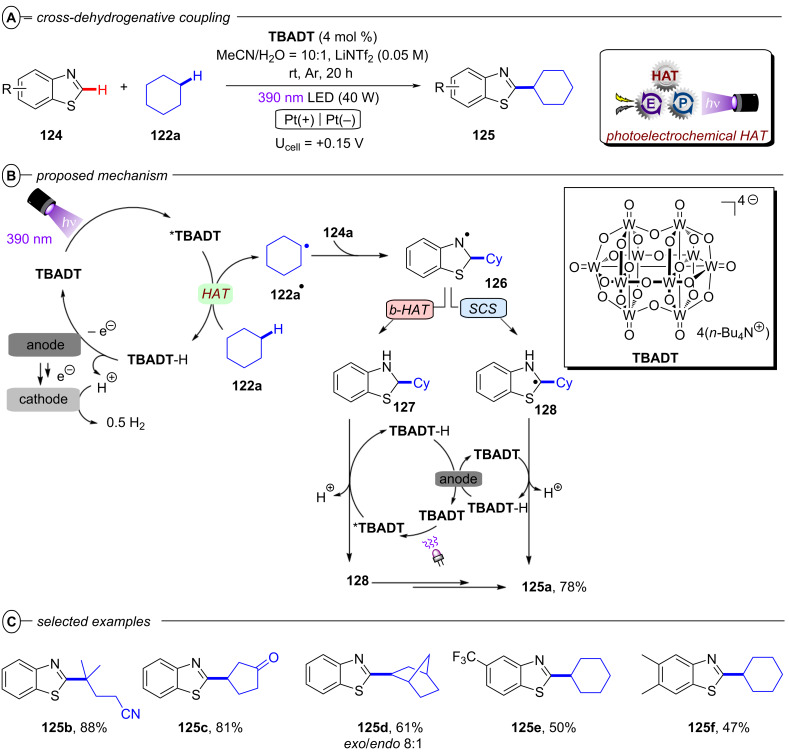
A) Photoelectrochemical HAT-mediated dehydrogenative coupling of benzothiazoles with aliphatic C–H bonds using **TBADT** as catalyst. B) Proposed mechanism. C) Selected examples from the substrate scope.

A seminal report in the photoelectrochemical HAT field was made by Ravelli and co-workers in a cross-dehydrogenative coupling (CDC) of benziothiazoles **124** and aliphatic C–H bonds (e.g., cyclohexane **122a** in Figure 58A) [252] using tetrabutylammonium decatungstate (**TBADT**, (*n-*Bu_4_N)_4_[W_10_O_32_]) [253]. The authors studied a plausible mechanism through kinetic analysis and laser flash photolysis (LFP). In particular, the excited state of **TBADT** is generated upon light irradiation (Figure 58B). *TBADT can activate unactivated C(sp^3^)–H bonds (such as those of cyclohexane **122**) via HAT to yield the carbon-centered radical **122a^•^**, which adds to the 2-position of benzothiazole **124** to generate the radical intermediate **126**. The authors then proposed two different pathways to access the target coupling product **125a**. Firstly, **126** undergoes to a back-HAT (*b*-HAT) from the reduced form of the catalyst, **TBADT**-H to generate the compound **127**, that affords the product through a photochemical oxidative cascade process where *TBADT acts as the oxidant. In the second possibility, **126** can undergo a proton-mediated spin center shift (SCS) process to give **128** and then **TBADT** serves as reductant to regenerate aromacity. Regarding the substrate scope, different benzothiazoles and C(sp^3^) radical precursors were envisioned, affording coupling products such as **125b**–**f** in moderate to high yields (47–88%, Figure 58C).

**Figure 59:**
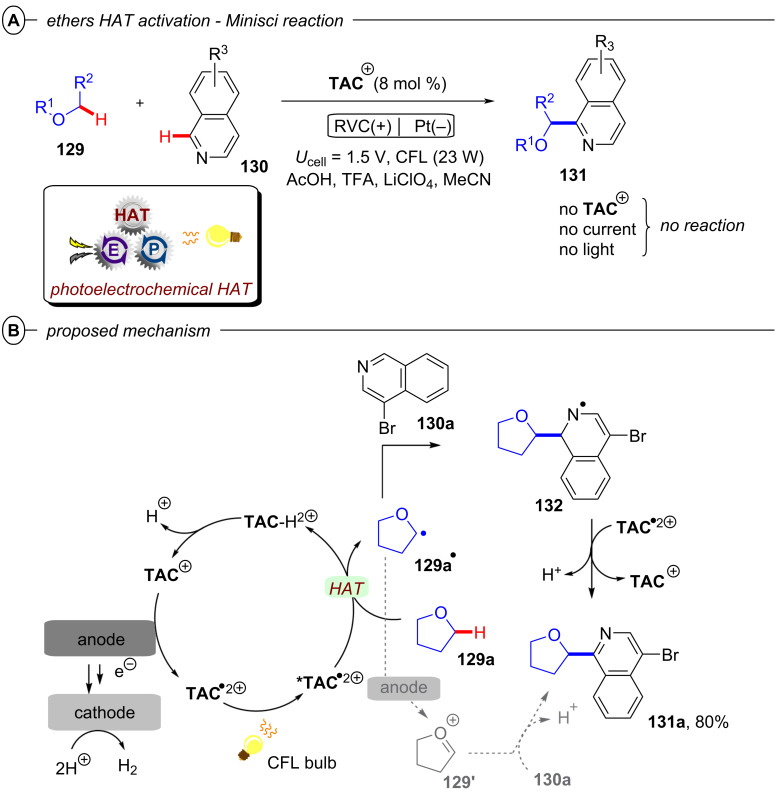
A) Photoelectrochemical HAT activation of ethers using electro-activated **TAC^+^** as photocatalyst. B) Proposed mechanism. C) Selected examples from the substrate scope.

**Figure 60:**
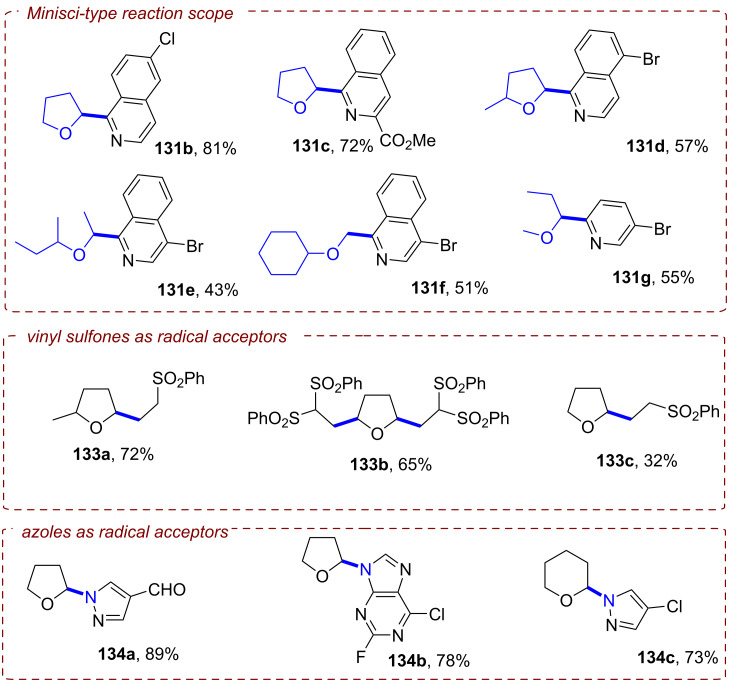
Selected examples from the substrate scope.

Lastly, Lambert and co-workers reported a PEC protocol for coupling alkyl ethers **129** with isoquinoline derivatives **130** involving **TAC^+^** as catalyst (Figure 59A) [254]. As usual, **TAC^+^** initially undergoes anodic oxidation and then photoexcitation to afford *TAC^•2+^ (Figure 59B). Rather than SET oxidation of the substrate, authors proposed HAT (supported by kinetic isotopic effect of *k*_H/D_ = 3.0) directly from the tetrahydrofuran **129a** to *TAC^•2+^ to generate the corresponding radical **129a^•^** and the protonated form of the photocatalyst **TAC-H^2+^**. At this stage, carbon-centered radical **129a^•^** can undergo coupling reaction with **130a** to yield the intermediate **132**. Subsequently, a second oxidation occurs via *TAC^•2+^ along with the loss of a proton, resulting in the formation of the target product **131a**. According to the authors, it is not possible to completely exclude SET activation of the ether partner (the KIE could also be attributed to deprotonation of the ether radical cation), nor possible to rule out the alternative pathway from C-centered radical **129a^•^** by SET oxidation to the oxocarbenium **129’** followed by nucleophilic addition of **130a** (grey dashed pathway in Figure 59B). With the optimized conditions in hand, a variety of isoquinoline partners tested with **129a**, giving rise to products like **131a**–**c** in very good to high yields (72–81%) (Figure 60).

Regarding the regioselectivity using different ethers, 2-methyltetrahydrofuran generated single regioisomers such as **131d**. Interestingly, while a notable regioselectivity for primary over tertiary C−H ethers bonds was observed (**131e** and **131f**), the competition between primary and secondary C−H bonds resulted in substitution at the secondary position (**131g**). Thus, in the latter case, the greater stability of the intermediate radical outweighed the steric difference. The authors extended the method to vinyl sulfones (**133a**–**c**) – due to ease of removing the phenylsulfonyl group – and to azoles (**134a**–**c**).

**Figure 61:**
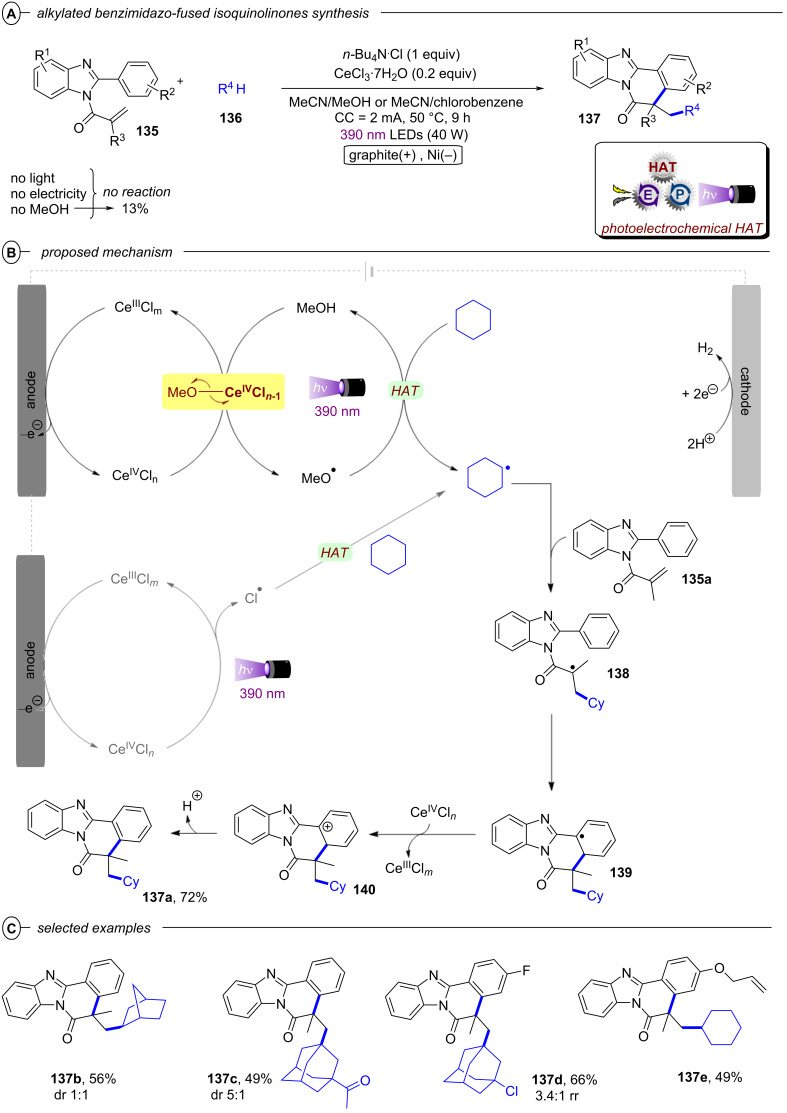
A) Photoelectrochemical HAT-mediated synthesis of alkylated benzimidazo-fused isoquinolinones using CeCl_3_ as catalyst. B) Proposed mechanism. C) Selected examples from the substrate scope.

**3.1.3 Alkene and alkyne activation: *Alkene activation*:** Benzimidazo-fused isoquinolines are recurrent motifs in many pharmaceutical products (e.g., antidiabetic and antitumor agents) and advanced organic materials (e.g., organic electronics and organic colorant, the so-called ‘carbonyl-dyes’) [255–256]. Classical methods for obtaining these motifs consist of high-temperature (130–150 °C) condensation reactions [257], or milder yet more expensive rhodium-based catalyzed [4 + 2] annulation conditions [258]. Owing to these drawbacks, the development of new cutting-edge strategies for their synthesis has therefore attracted interest. Several protocols have been reported in recent years, showing how these motifs can be obtained from radical addition/cyclization cascade processes [259–260]. Aliphatic carboxylic acids [261], alkyl boronic acids [262], *N*-hydroxyphthalimide esters (NHPI esters) [263–264] or Katrizky salts [265] can all be used as radical precursors under photoredox or electrochemical conditions. Naturally, these are not so atom economical and the use of non-prefunctionalized alkyl radical precursors would increase atom economy. Although a step in this direction has been taken by the group of Wei and co-workers [266], a potentially explosive peroxide radical was necessary. However, recently, Xu, Zeng and co-workers reported an interesting PEC cerium-catalyzed radical addition/cyclization process for the incorporation of unactivated alkanes **136** as radical precursors [267], by exploiting a PEC HAT to generate an alkyl radical directly from the unactivated alkane (as described previously by Xu, Ravelli and Lambert; Figures 56–59). This generated alkylated benzimidazo-fused isoquinolinones and other correlated N-bearing cycles **77** starting from compounds like **135** (Figure 61A) [267].

The proposed mechanism was supported by kinetic isotope effect (KIE) experiments, cyclic voltammograms (CV), UV–vis spectroscopy and comparisons with previous reports [238,268–269]. Anodic oxidation of Ce^III^ in the presence of *n*-Bu_4_N∙Cl and MeOH was proposed to afford the complex MeO−Ce^IV^Cl*_n_*_–1_, which undergoes homolytic cleavage through a ligand to metal charge transfer (LMCT) under 405 nm LED irradiation. The authors claimed formation of MeO^•^ (Figure 61B), rather than Cl^•^, on the basis that other alcohols such as hexafluoroisopropanol (HFIP), CF_3_CH_2_OH and CCl_3_CH_2_OH led to lower product yields. At this point, the HAT process from cyclohexane to electrophilic MeO^•^ affords the cyclohexyl radical, which undergoes radical addition to the double bond of **135a** to yield intermediate **138**, which can then cyclize to **139**. Finally, SET oxidation of the latter by Ce(IV) and subsequential deprotonation of **140** generates the benzimidazo-fused isoquinolinone **137a**. The alternative possibility of LMCT to afford Cl^•^ cannot be excluded (grey pathway in Figure 61B), especially since i) a 13% product yield was observed for the model reaction without MeOH and ii) different electrolytes like *n*-Bu_4_N∙Br or *n*-Bu_4_N∙I led to the model reaction product in only low or trace yield.

Regarding the substrate scope, the authors demonstrated that both secondary and tertiary C(sp^3^)−H-bearing compounds were successfully incorporated to afford the products in moderate to good yields (49–56% for **137b** and **137c** in Figure 61C). Then, the scope of *N*-methacryloyl-2-phenylbenzoimidazoles was explored, e.g., with 1-chloroadamantane or cyclohexane, resulting in products such as **137d** and **137e** in moderate to good yields (49–66%).

***Alkyne activation*:** α,α-Dihaloalkyl derivatives play a fundamental role in pharmaceuticals and natural products like AML inhibitors and antiviral drugs [270–272]. They constitute important building blocks in the synthesis of various chemical intermediates like cyclopropanes and 2-keto(hetero)aryl benzox(thio)azoles [273–275]. Methods for their synthetic access have garnered attention, the most straightforward of which is the direct oxydichlorination of alkynes. There are excellent examples of this process in the literature [276–280], but all suffer from several limitations. Among them are the use of excess strong chemical oxidants, divided cell direct electrolysis (high cell resistance), strongly acidic conditions or atom uneconomical chlorinating agents like *N*-chlorosuccinimide (NCS).

**Figure 62:**
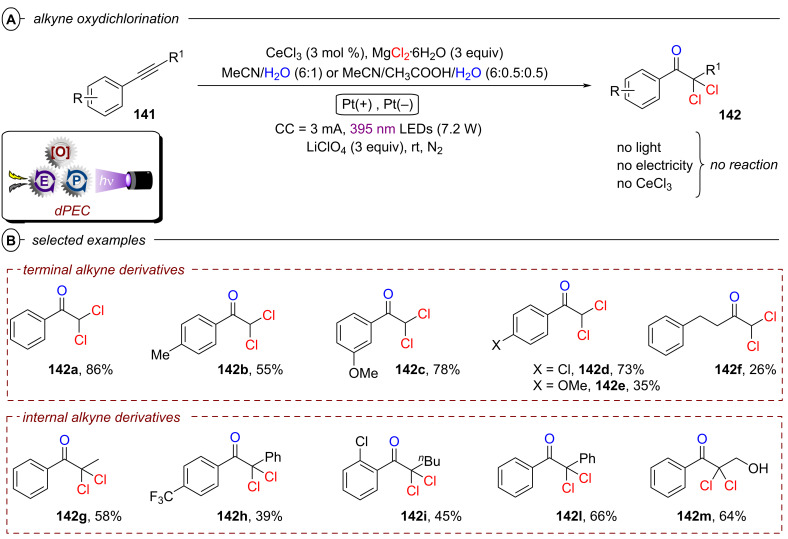
A) Decoupled photoelectrochemical cerium-catalyzed oxydichlorination of alkynes using CeCl_3_ as catalyst. B) Selected examples from the substrate scope.

**Figure 63:**
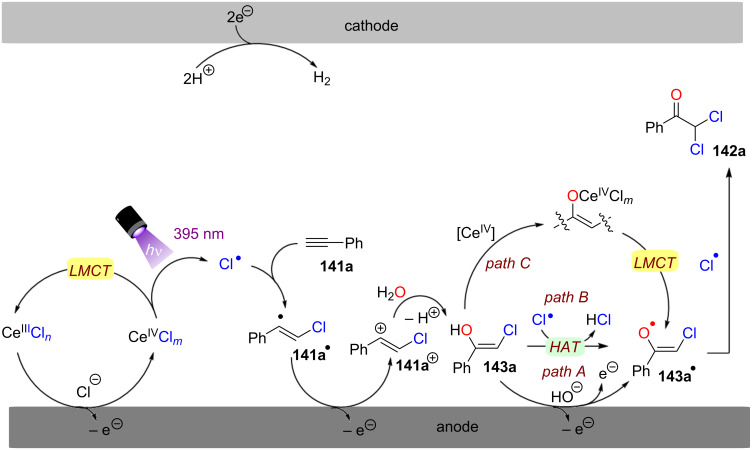
Proposed decoupled photoelectrochemical mechanism.

Hence, Chen and co-workers reported a *d*PEC oxydichlorination starting from alkynes such as **141**, catalyzed by CeCl_3_ under PEC conditions (Figure 62A) [281]. The optimized conditions employed LiClO_4_ as electrolyte and MgCl_2_∙6H_2_O as a source of Cl^•^ (a much cheaper and more atom economical source in comparison to NCS) in a mixed MeCN/water solvent. The authors examined numerous arylalkynes and observed that ethynylbenzene and substrates bearing benzylic alkyl substituents were successful (**142a**,**b**, Figure 62B). Both electron-rich and -poor substituents were tolerated affording products in very good yields (73–78% for **142c**,**d**), while 4-MeO-substituted phenylacetylene mostly polymerized and only gave a modest product yield (**142e**). The method was also tested on an aliphatic substrate (4-phenyl-1-butyne), resulting in the product **142f** in 26% yield. Although the authors did not investigate further the reactivity between aromatic and aliphatic alkynes, it is proposed that the low efficiency of the latter is due to the absence of the aromatic ring to stabilize the reactive intermediate **143a** or **141a^•^** (Figure 63). Internal acetylenes were also successful, affording alkyl-substituted dichloroketones such as **142g**. A series of electron-poor functional groups (halogen, trifluoromethyl) were compatible (**142h**,**i**), a diarylalkyne and propargylic alcohol were tolerated (**142l**,**m**).

Concerning the mechanism, the authors proposed anodic oxidation of CeCl_3_ to a [Ce^IV^Cl_m_] species, followed by release of Cl^•^ via photoinduced LMCT (Figure 63). Trapping of Cl^•^ by the alkyne **141a** affords alkene radical intermediate **141a^•^**, whose oxidation leads to carbocation **141a^+^**. Subsequential nucleophilic addition of water and deprotonation was invoked to access enol **143a**. The latter is transformed to enol radical **143a^•^** via a tandem process of deprotonation and anodic oxidation (*path A*). The final product **142a** is formed via chlorine radical termination with **143a^•^**. Alternatively, the process **143a** → **143a^•^** could be achieved either by the HAT reactivity of Cl^•^ (*path B*) or the LMCT activity of a Ce^IV^ enolate (*path C*).

**3.1.4 (Hetero)cycles activation:** The selective cleavage and functionalization of C−C bonds is a strategy of growing importance in organic synthesis [282–284]. In particular, the β-scission of C–C bonds in alcohols has proven a key target owing to the privileged abundance of these moeities in biomass chemical feedstocks [285–286]. The key driving force for β-scission is the formation of a strong C=O bond from an alkoxy radical, which is challenging to generate due to its high O–H bond dissociation energy (≈105 kcal mol^−1^). Strong, stoichiometric chemical oxidants such as bromine, hypervalent iodine agents or SelectFluor^®^ have been previously used to achieve this [287–291]. Thus, the development of milder and greener protocols without stoichiometric oxidants is desirable and one such reported approach leverages PEC.

**Figure 64:**
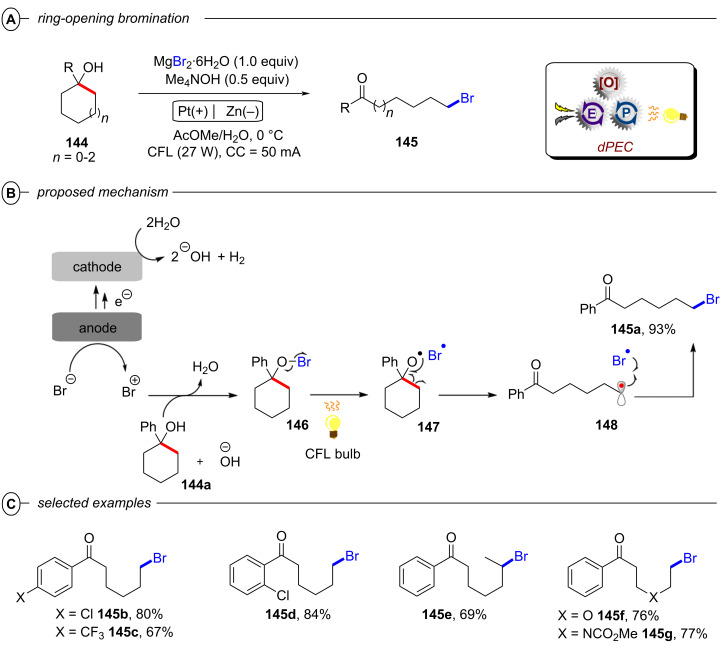
A) Decoupled photoelectrochemical ring-opening bromination of tertiary cycloalkanols using MgBr_2_ as Br^+^ source. B) Proposed mechanism. C) Selected examples from the substrate scope.

Onomura and co-workers first realized a PEC ring-opening bromination of unstrained *tert*-cycloalkanols **144** to afford ω-bromo-substituted ketones **145** by using MgBr_2_·6H_2_O as a source of Br*^+^* (Figure 64A) [292]. This paper contributes to the vast field of halogenated ketone synthesis, which is important since these compounds constitute versatile building blocks in the formation of various heterocyclic compounds (e.g., cyclic imines) [293–295], functional materials (e.g., conjugated polymers and liquid crystal dimers and trimers) [296–298] and biologically-active molecules (e.g., 5-HT4 receptor agonists and 5-HT7 receptor ligands) [299–301]. The optimized conditions reported by the authors consist of Me_4_NOH as both a base and phase transfer catalyst (PTC) in a mixture of AcOMe/H_2_O at 0 °C using an undivided cell and a CFL bulb (27 W).

In the mechanism, the bromide ion is SET oxidized (twice) at the anode to generate the bromine cation species (Figure 64B). The reaction between the latter and the starting material **144a** yields a hypobromite intermediate **146**, which can undergo light-promoted homolytic cleavage of the O−Br bond to generate the alkoxy radical **147**. Then, the latter is trapped by a bromo radical (or other hypobromite species) to afford the radical carbon intermediate **148**, which reacts with Br^•^ to yield the ω-bromoketone **145a**. Regarding the substrate scope, cyclohexanols bearing an electron-withdrawing group such as chloro (**145b**) or trifluoromethyl (**145c**) afford the target product in good to high yields (67–80% in Figure 64C). The steric hindrance of an *ortho-*Cl atom did not compromise the yield of **145d**. Unsymmetrical substrates led selectively to the products like **145e** via regioselective β-scission to generate the more stable (secondary) alkyl radical. Finally, tetrahydropyranol and piperidinol derivatives were successfully transformed into the desired products in very good yields (76–77% for **145f**,**g**).

**Figure 65:**
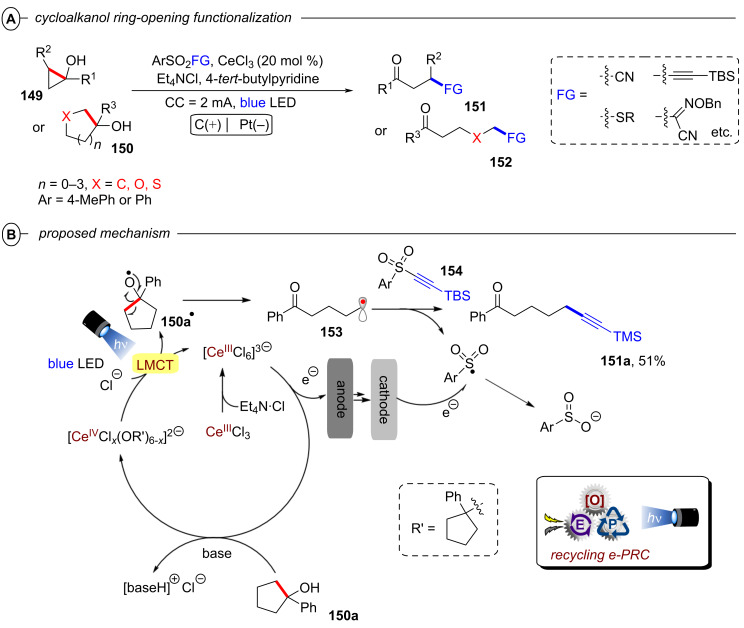
A) Recycling e-PRC ring-opening functionalization of cycloalkanols using CeCl_3_ as catalyst. B) Proposed mechanism.

Subsequently, Lei and co-workers reported a redox-neutral PEC method for ring opening functionalization of cycloalkanols **149** or **150** using CeCl_3_ as catalyst (Figure 65A) [268]. Their protocol allowed cleavage of cycloalkanols with different sizes and tolerated a vast range of functional groups, allowing the scope of downstream functionalization to be notably broadened beyond bromination. Mechanistically, CeCl_3_ is solubilized in MeCN with the help of Et_4_N∙Cl, leading to a [Ce^III^Cl_6_]^3^**^−^** complex which was identified as the active catalyst state (Figure 65B). The latter is oxidized at the anode to Ce(IV), which immediately reacts with the cyclic alcohol **150a** to yield a Ce(IV)-alkoxide species via ligand exchange in the presence of base. Thereafter, the Ce(IV)-alkoxide undergoes a photoinduced LMCT homolysis process to form radical **150a^•^** and regenerate the [Ce^III^Cl_6_]^3^**^−^** complex. The former undergoes β-scission to break the C–C bond, resulting in radical **153**. Radical addition–elimination of **153** to the arylsulfonyl compound (such as *tert*-butyldimethyl(tosylethynyl)silane **154** in Figure 65B) generates product **151a** and liberates the sulfonyl radical, which is then reduced at the cathode to form a nontoxic benzenesulfonate. Therefore, and notably, this reaction occurs in an overall redox neutral fashion.

**Figure 66:**
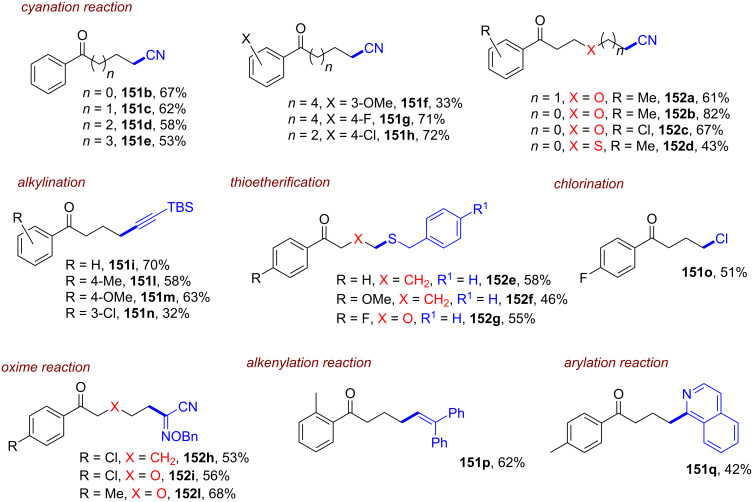
Selected examples from the substrate scope of the PEC ring-opening functionalization.

The optimized conditions were applied to cycloalkanols of different ring sizes – from a three-membered ring to a seven-membered ring – in an overall fragmentative cyanation reaction affording the desired products (**151b**–**e**) in moderate to good yields (53–67% in Figure 66). Electron-donating groups and electron withdrawing groups (**151f**–**h**) were all tolerated. Interestingly, heterocyclic alcohols were tolerated, giving the desired products **152a**,**d** in moderate to high yields (43–82%). Together with the protocol of Onomura and co-workers (Figure 64), this shows how PEC provides an oxidative platform for engaging cyclic alcohols and amines that is complementary to the reductive conPET strategy for the carboxylative ring-opening of cyclic amines (vide supra, Figure 22). Finally, the protocol could be extended to engage intermediate **153** in downstream alkynylations (**151i**–**n**), thioetherifications (**152e**–**g**), chlorinations (**151o**), alkenylations (**151p**), arylations (**151q**) and reactions with oximes (**152h**–**l**), demonstrating a notably broad scope of applications.

#### 3.2 Reductive activation

**3.2.1 Arene C(sp^2^)−X activation:** As mentioned in the introduction (vide supra), a key focus of PRC in recent years and justification in the efforts to merge photo- and electrochemistry has been on the development of novel approaches for achieving extreme reduction potentials (*E*^p^_red_ < −2 V vs SCE) without using dissolving alkali metals. While the concomitant conPET approach represented an important initial step towards a safe and chemoselective protocol, it has certain limitations that need to be addressed. These limitations include i) the need for multiple photons which are expensive to generate, ii) the requirement for a carefully balanced system in which both catalyst oxidation states absorb light and undergo PET processes under the same reaction conditions, iii) the interference of byproducts from sacrificial electron donors in the reaction pathway (and their separation from the desired products). A typical example of the latter is a trialkylamine such as Et_3_N, which can i) limit the steady-state concentration of (radical anion) reductive photocatalyst/hinder downstream reactivity by back electron transfer to Et_3_N^•+^, ii) promote intermediate radical quenching via HAT (either involving Et_3_N^•+^ or the derived α-amino radical), iii) effect other pathways like XAT (from the derived α-amino radical).

Therefore, researchers identified electrochemistry as a direct and more flexible alternative for generating a high steady-state concentration of (radical anion) reductive photocatalyst from its neutral precursor via cathode reduction. By using a divided cell configuration, the risk of interference from oxidized byproducts can be suppressed or entirely eliminated.

**Figure 67:**
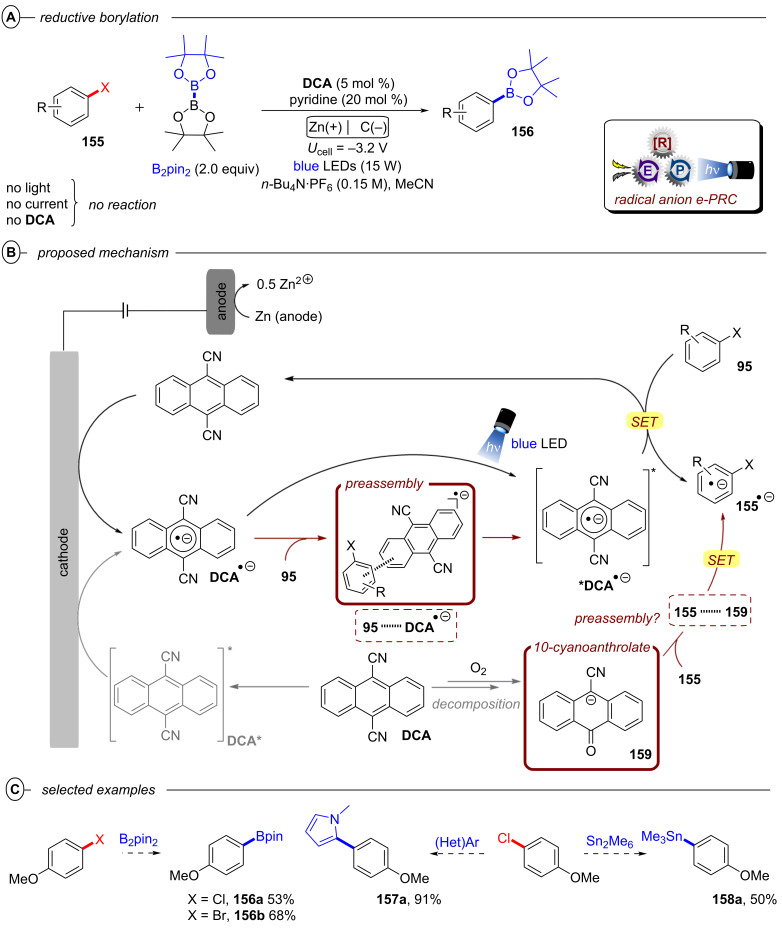
A) Radical ion e-PRC reduction of chloro- and bromoarenes using **DCA** as catalyst and various acceptors as trapping agents. B) Proposed mechanistic pathways (by authors, black/grey arrows; by us, burgandy arrows). C) Selected examples from the substrate scope.

With this idea in mind, Lambert, Lin and co-workers reported a radical ion e-PRC reduction of chloro- and bromoarenes **155** using 9,10-dicyanoanthracene (**DCA**) as catalyst (Figure 67A) [302]. In their mechanistic proposal, the **DCA** catalyst undergoes cathodic reduction to generate **DCA^•−^**, which is then photoexcited to afford a powerful reductant [52]. The authors proposed that *DCA^•−^ can donate an electron to the aryl halide **155** to furnish intermediate **155^•−^** and to regenerate the **DCA** catalyst (Figure 67B). Aryl halide radical anion **155^•−^** then undergoes mesolytic C(sp^2^)–X cleavage to form an aryl radical that is trapped by an electrophile (B_2_Pin_2_, (Het)Ar or Sn_2_Me_6_ respectively) to furnish the target products. The authors could not rule out the possibility of a photoinduced reductive quenching of **DCA** at the cathode to form **DCA^•^**^−^ (grey pathway in Figure 67B). However, this seems implausible because i) **DCA** is not efficiently excited by the blue LEDs and ii) the Beer–Lambert dependence would dictate light penetration only in the bulk solution within a thin film at the reactor walls, shielding transmission of light to the electrode. Remarkably, electron-rich aryl halides like 4-bromo- and 4-choroanisole could be reductively engaged in SET, leading to their corresponding borylated (**156a**,**b**), (hetero)coupled (**157a**) and stannylated products (**158a**) (Figure 67C). Without delving into the details of substrate scope that was concisely described in a previous review [28], it is interesting to further elaborate on the mechanism and to compare with the conPET report (vide supra, Figure 9).

According to the estimation of the authors made by the Rehm–Weller equation [303], *DCA^•−^ displays an exceptionally high reducing potential of *E_1/2_ = −3.2 V vs SCE. However, this value is ≈0.6 V more negative than the value calculated by Jacobi von Wangelin, Pérez-Ruiz and co-workers [52] and recently by Vauthey and co-workers [53] (section 2.1.1, vide supra). The difference lies in the fact that the −3.2 V value was calculated on the basis of an excited-state energy of 2.38 eV (and not the D_1_ state energy of 1.75 eV) extracted from the absorption and emission spectra presented in the work of Eriksen [304], which was later found to be associated with a follow-up reaction product of **DCA^•−^** (discussed below). This follow-up reaction product was also responsible for the nanosecond lifetime (13.5 ns) that was incorrectly ascribed to *DCA^•−^ (τ ≠ 13.5 ns), with the correct picosecond lifetime being later reported by Vauthey (τ ≈ 3 ps).

Photophysical properties aside, a very interesting aspect is that the conceptually analogous conPET protocal using **DCA** (vide supra, Figure 9) [52] *did not achieve reductive activation of electron-rich aryl halides like haloanisoles* (affording – at most – 6% yield). Since this discrepancy between the reports has not been previously addressed, we propose two different explanations:

i) the steady-state concentration of **DCA^•−^** generated under e-PRC favors a preassembly with the substrate, that may enable access to higher order excited states (D_n>1_) of *DCA^•−^ and/or facilitate cleavage of [Ar−X]^•−^ that is likely rate-determining;

ii) the active species involved in the process was not the radical anion species *DCA^•−^, but rather a 10-cyanoanthrolate species (**159** in Figure 67B). The 10-cyanoanthrolate is known to form in quantitative yield during cathodic reduction of **DCA** without rigorous exclusion of oxygen [305–306], and gradually further transforms to anthraquinone (which Lambert, Lin and co-workers reported was also a successful catalyst precursor, albeit affording a lower yield of product compared to the **DCA** precursor). The brown color and UV–vis bands of the reaction mixture reported by Lambert, Lin and co-workers [302] corresponds better with the 10-cyanoanthrolate spectra of Breslin and Fox [305] than with the purple color and UV–vis bands of **DCA^•−^**.

However, although the formation of 10-cyanoanthrolate in the reaction of Lambert, Lin and co-workers is obvious, there is no evidence to suggest its excited state is reductive enough to engage the electron-rich aryl halides. König and co-workers reported a 9-anthrolate photocatalyst without substituents at the 10-position that could only engage electron-poor aryl chlorides [307]. Having a 10-cyano substituent would stabilize the anthrolate further and thus render its excited state even less reducing.

Overall, we conclude based on the evidence available so far that either *DCA^•−^ (D_n>1_) is the active catalyst via a preassembly with the aryl halide, or 10-cyanoanthrolate preassembles with the aryl halide in a manner that accelerates cleavage of [Ar−X]^•−^.

**Figure 68:**
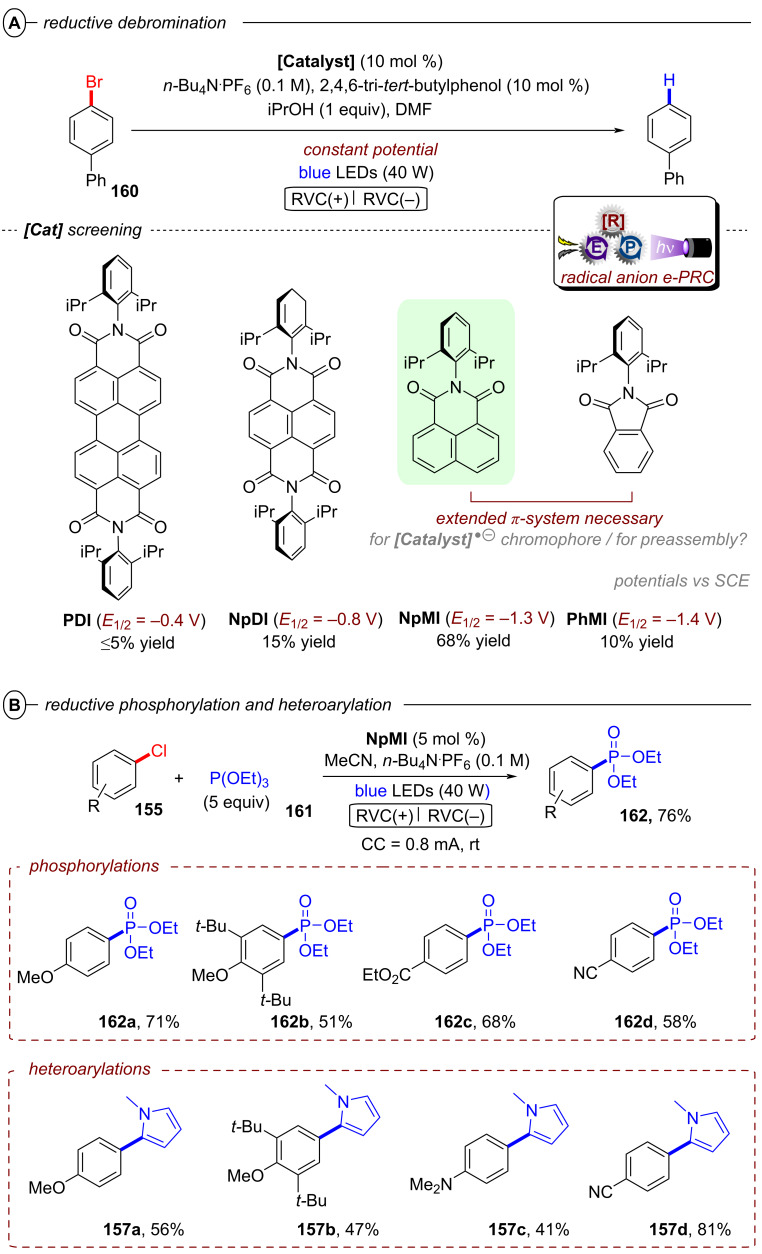
A) Screening of different phthalimide derivatives as catalyst for the e-PRC reduction of aryl halides. B) e-PRC Arbuzov reaction of aryl chlorides using **NpMI** as catalyst (top) and selected examples (bottom).

Simultaneously to the report of Lambert, Lin and co-workers, Wickens and co-workers reported a noteworthy investigation on the reductive dehalogenation and activation of aryl halides [308]. Initially, the authors investigated the dehalogenation reaction of 4-bromobiphenyl **160** (*E*^p^_red_ = −2.40 V vs SCE), as depicted in Figure 68A [308]. Four arylimide catalysts were tested under appropriate conditions of constant potential and visible light (Figure 68A). Perylene diimide (**PDI**, *E*_1/2_ = −0.43 V vs SCE) was found to be poorly effective (<5%) in the reaction, whereas the naphthalene-based analogues, **NpDI** (*E*_1/2_ = −0.8 V vs SCE) and **NpMI** (*E*_1/2_ = −1.32 V vs SCE), exhibited much improved yields (15% and 68%, respectively). This finding is intriguing since the reactivity of **PDI** was known both under electrochemical and photochemical conditions (after excitation with two photons, vide supra) [15,309], whereas the reactivities of **NpDI** and **NpMI** derivatives were previously little explored, despite photophysical experiments indicating they would furnish more potent reductants by electron-priming [310]. The screening of catalysts concluded with the phthalimide derivative **PhMI** (*E*_1/2_ = −1.4 V vs SCE), which was far less effective than **NpMI** despite a comparable redox potential. The extended naphthalene π-system is clearly necessary (either for photoexcitation or to promote a preassembly with the target substrate).

Thus, the Wickens group employed **NpMI** as catalyst for an e-PRC Arbuzov reaction [311], beginning with aryl chlorides (**155** in Figure 68B) [308]. Generally, the photo-Arbuzov reaction refers to a carbon–phosphorus bond-forming reaction that proceeds through an aryl radical intermediate to generate a pentavalent phosphorus species (vide supra, section 2.1.1) [312]. Wickens’ group utilized triethylphosphite **161** to trap the aryl radical generated via SET to the chloride from the radical anion *NpMI^•−^ (*E_1/2_ = −2.81 V vs SCE), the latter of which was generated by cathodic reduction and photoexcitation (Figure 68B).

Aryl chloride substrates bearing potentially sensitive functional groups such as esters (**162c**) and nitriles (**162d**) were suitable for SET phosphorylation. Overall, good yields of products were observed, providing a PEC strategy that is complementary to the subsequently reported conPET method (discussed vide supra, Figure 11). The method was expanded to heteroarylations using *N*-methylpyrrole as the trapping reagent, affording products **157a**–**d** in moderate to high yields (41–81%).

In particular, the reaction successfully engaged 4-chloroanisole (**162a**) and an even electron-richer aryl chloride (*E^p^*_red_ = −3.4 V vs SCE) was converted (**162b**). This result indicates that *NpMI^•−^ possesses a reductive redox power that is i) comparable to the photoactive species in the study of Lambert, Lin and co-workers [302] and ii) comparable to that of Li^0^ (−3.3 V vs SCE). Finally, both reports from the groups of Lambert/Lin and Wickens found that e-PRC gave higher preparative yields compared to direct electrolysis conditions [313], and dehalogenation was not observed, demonstrating the key selectivity benefit of e-PRC compared to electrolysis alone.

**Figure 69:**
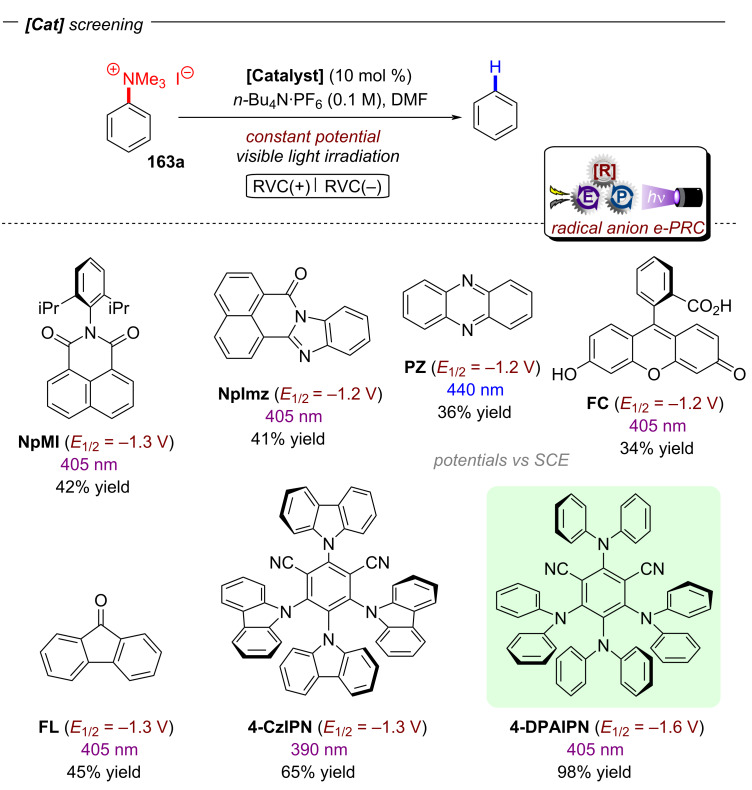
Screening of different organic catalysts for the e-PRC reduction of trialkylanilium salts.

**Figure 70:**
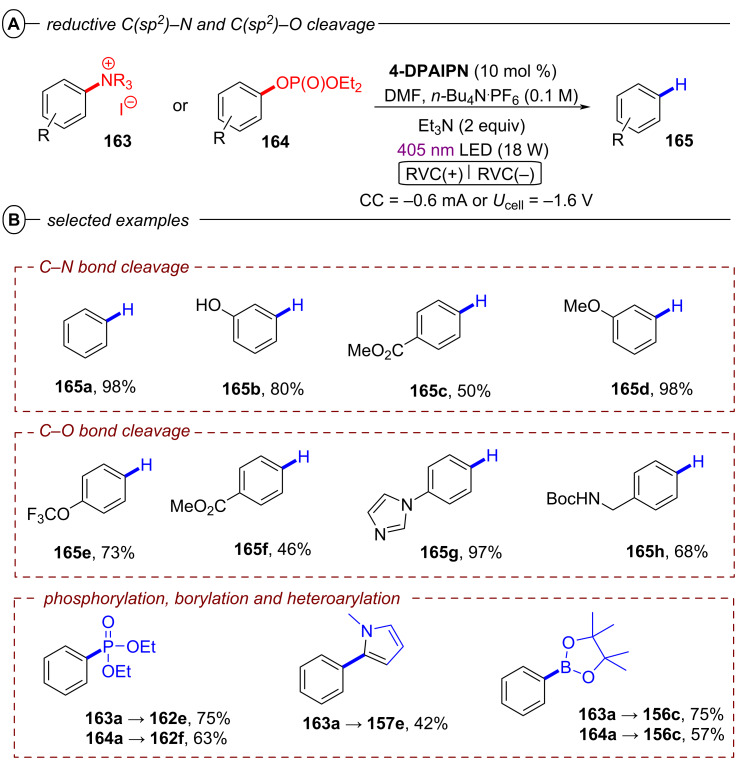
A) e-PRC reduction of phosphonated phenols and anilinium salts. B) Selected examples from the substrate scope.

Following their investigation of the e-PRC reductive activation of arenes, Wickens and co-workers extended their methodology for reducing aryl halides to the reduction of aryl pseudohalides such as trialkylanilinium salts **163** and phosphonated phenols **164** (Figure 69) [314]. Initially, they screened various precatalysts that might, upon electro-activation and photoexcitation, serve as potent reductants to convert *N*,*N*,*N*-trimethylanilinium salt **163a** to benzene. Among the tested species, **NpMI** and its derivatives, such as **NpImz**, led to acceptable yields of benzene (41–42%). Precursors to persistent organic radical anions, including phenazine (**PZ**), fluorescein (**FC**), and 9*H*-fluoren-9-one (**FL**), generated similar yields of the product (34−45%). By contrast, employing isophtaionitrile scaffolds led to a substantial improvement in yield, with 1,2,3,5-tetrakis(carbazol-9-yl)-4,6-dicyanobenzene (**4-CzIPN**) [315] resulting in a 65% yield, while **4-DPAIPN** [316] gave the highest (98%) yield of benzene. Consequently, the latter compound was selected for the subsequent experiments, wherein C(sp^2^)–N and C(sp^2^)–O cleavage processes were performed using a constant potential of −1.6 V and 405 nm (Figure 70A).

The reaction conditions were well-tolerated by free alcohols (**165b**), esters (**165c**), and ethers, enabling C(sp^2^)–N reduction in moderate to excellent yields (50–98%). In addition, despite the deep reduction potentials of phenol derivatives (*E*^p^_red_ < −2.7 V vs SCE), a wide range of phosphate esters with base-sensitive functional groups yielded the products **165e**–**h** in moderate to excellent yields (46–97%) (Figure 70B). Aryl radical intermediates derived from anilinium salts and phosphonate esters could also be intercepted by trapping agents. The C(sp^2^)–N and C(sp^2^)–O precursors were found to undergo borylations, phosphonylations, and heteroarylations, affording products **162e,f**, **157e**, and **156c**.

**Figure 71:**
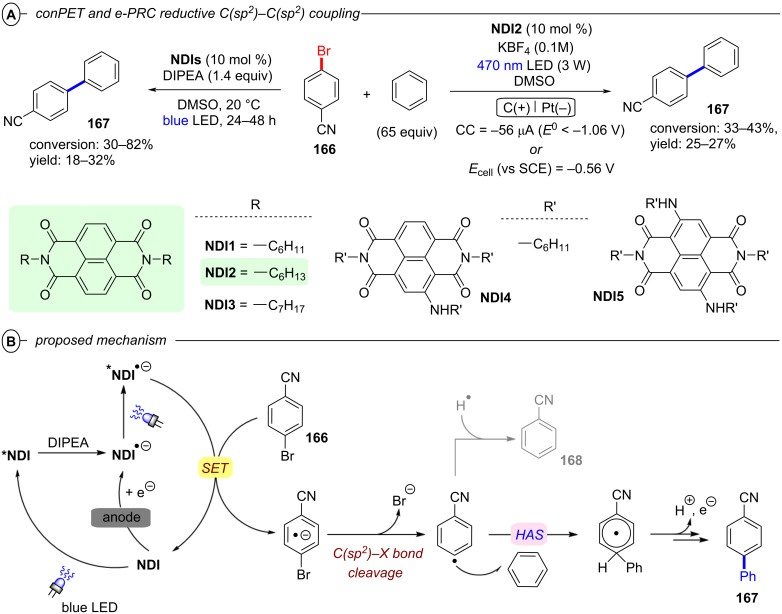
A) ConPET and e-PRC reduction of 4-bromobenzonitrile using a naphthalene diimide (**NDI**) precatalyst and aryl radical coupling with benzene. B) Proposed mechanism for conPET and e-PRC protocols.

Lastly, Bardagi and co-workers conducted a direct comparison of reductive aryl halide coupling reactions mediated by conPET and radical ion e-PRC [317]. Specifically, they investigated a family of naphthalene diimides (**NDI**) as (electro-activated) photocatalysts for the reductive coupling of 4-bromobenzontrile (**166**) with benzene. The authors synthesized a range of diimides from commercially available naphthalene-1,4,5,8-tetracarboxylic acid dianhydride (**NDA**) and a variety of amines, leading to **NDI1**–**5** shown in Figure 71A [318]. Under conPET conditions, DIPEA served as a sacrificial electron donor and DMSO as a solvent. Intriguingly, all catalysts promoted the cross-coupling, with **NDI2** delivering the highest yield of **167** (32%) after 48 h, and benzonitrile (**168**, 23% yield) as a byproduct. Encouraged by these results, the authors tested the same catalyst under e-PRC conditions, evaluating both constant current and potential conditions in a divided cell setup with graphite and platinum electrodes. The optimal conditions involved a constant current of −56 µA (employing a potential cutoff *E*^0^ < −1.06 V) or a constant potential of −0.56 V. After 48 h, conditions afforded **167** with a yield of 25–27% and only 6% of benzonitrile. These findings led the authors to conclude that both conPET and e-PRC approaches are comparably effective for such reductive couplings of aryl halides in terms of yields. While a conPET process requires a simpler setup, the e-PRC method improves the selectivity with respect to hydrodehalogenation (presumably due to the absence of DIPEA^•+^ as a H atom donor).

Regarding the mechanistic comparison (Figure 71B), blue LED irradiation of **NDI** in the presence of DIPEA generated **NDI^•−^** via the conPET pathway, while cathodic reduction was employed in the e-PRC approach. The authors reported that the concentration of **NDI1^•−^** was ≈5 × 10^−5^ M, indicating that PET from DIPEA to NDI1* was efficient and BET were sufficiently slow to ensure build up of a stable concentration of the radical anion in the solution. Interestingly, electrochemical generation of **NDI1^•−^** led to even higher concentrations (≈0.7–2.0 × 10^−4^ M), possibly due to the absence of sacrificial electron donors in the cathodic chamber which prevented BET. Following photoexcitation, the authors proposed SET from *NDI^•−^ to **166** (*E*^p^_red_ = −1.94 V vs SCE). This step was feasible, considering the estimated reduction potential of *NDI3^•−^ (*E_1/2_ = −2.14 V vs SCE) based on *E*_0-0_ = −1.64 V [310]. After this crucial step, rapid mesolytic cleavage of [**166**]**^•−^** occurred (10^9–11^ s^−1^) [76] to yield the aryl radical, which could either i) abstract a H atom from the solvent (or DIPEA^•+^) to generate byproduct benzonitrile (*grey path*) or ii) undergo homolytic aromatic substitution (HAS) with benzene to ultimately furnish coupling product **167**.

**Figure 72:**
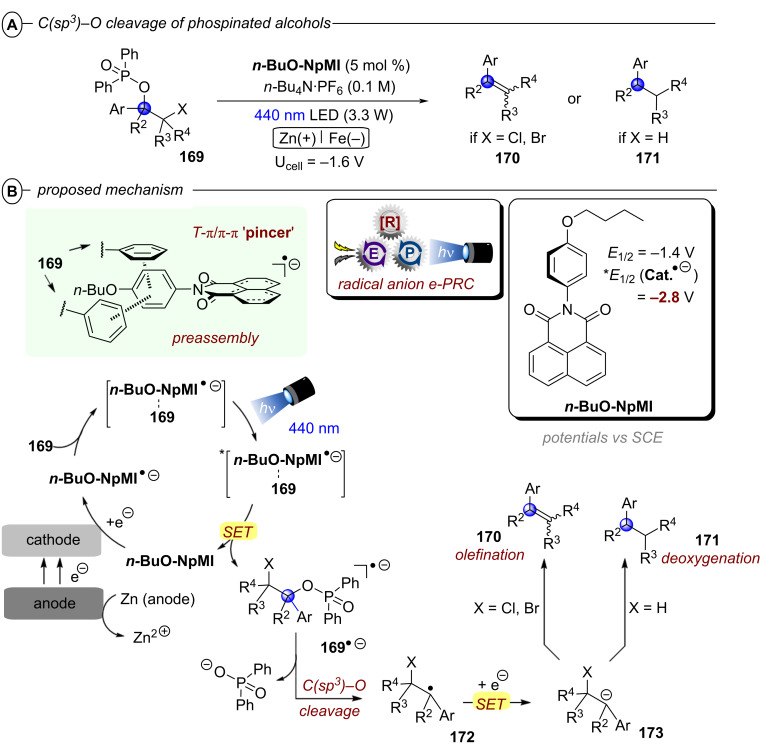
A) Radical ion e-PRC reduction of phosphinated aliphatic alcohols with ***n*-BuO-NpMI** as catalyst. B) Catalytic cycle including proposed preassembly.

**3.2.2 C(sp^3^)−X activation:** C(sp^3^)–O bond cleavages are high priority targets, since alcohols are widely abundant feedstocks deriving from nature and industrial hydroformylation processes. Classically, this is achieved by elimination (acid- or base-catalyzed) that can require high temperatures and do not tolerate sensitive functional groups, requiring additional protection steps elsewhere in the molecule. Within this context, Barham, König and co-workers reported the first e-PRC reductive cleavage of an aliphatic C(sp^3^)–X bond – namely C(sp^3^)–O bonds of phosphinates of alkyl alcohols **169** – giving rise to olefins or deoxygenated products (Figure 72A) [319]. Here, *N*-(*para*-butoxyphenyl)naphthalene monoimide (***n*-BuO-NpMI**) was employed as an electro-activated photocatalyst, affording either i) olefination products **170** in a Corey–Winter-type process if there is a leaving group ‘X’ α- to the alcohol (X = Cl, Br), or ii) deoxygenation products **171** when X = H as a mild and tin-free alternative to the Barton–McCombie process [320–321].

**Figure 73:**
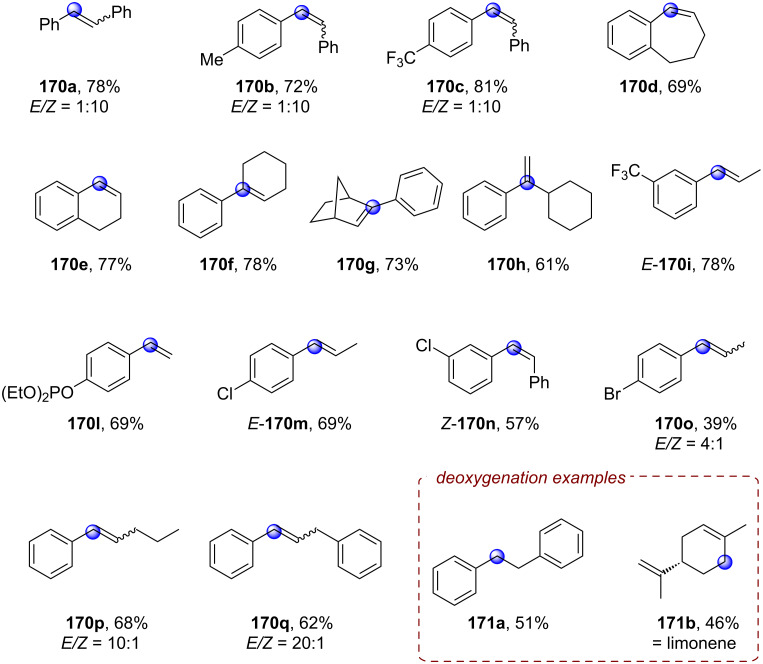
Selected examples from the substrate scope.

In the mechanism, ***n-*BuO-NpMI** is reduced at the cathode and its radical anion ***n*-BuO-NpMI]^•−^** engages phosphinate **169** in a preassembly. Photoexcitation affords a potent reductant, *[*n-*BuO-NpMI]^•−^ (*E_1/2_ = −2.8 V vs SCE) (Figure 72B). SET reduction of phospinate **169** (*E*^p^_red_ = −2.4 to −2.6 V vs SCE) affords radical anion **171^•−^**, effecting C(sp^3^)–O bond cleavage (which is likely facilitated by the assembly) to liberate the phosphinate and generate C(sp^3^) radical **172**. A further SET reduction (either by the cathode or by ***n-*BuO-NpMI^•−^**) provides carbanion **173** in a radical polar crossover [322–323]. With the optimized conditions in hand, the authors explored a wide range of olefination reactions (Figure 73).

Symmetrical and unsymmetrical *Z*-stilbenes **170a**–**c** were obtained in high yields with a tandem reduction/isomerization process under the PEC conditions. Interestingly, this represents the first example of a radical ion catalytic system that functions both as a potent SET reductant and in a subsequent *E*/*Z* isomerization. Cyclic olefins could be readily accessed, giving products **170d**–**g** in good to high yields (69–73%). This is an important achievement since such types of internal olefins are difficult to access with classical olefination reactions and typically require acid-/base-catalyzed eliminations whose site-selectivity is hard to control. For example, while terminal olefin **170h** was obtained by the e-PRC method, conventional dehydration of the corresponding tertiary alcohol would lead to the most substituted double C–C bond.

Interestingly, despite the deeply reducing electro-activated photocatalyst, the reaction tolerated reductively labile substituents such as Ar−CF_3_ (**170i**) and Ar−OP(OR)_2_ (**170l**, reductive cleavage of which was later reported by Wickens and co-workers using *[4-DPAIPN]^•−^, vide supra, Figure 70). *The authors thus questioned whether even halogens could be tolerated, which would go against all expectations from all the aforementioned conPET and e-PRC reports*. For this purpose, phosphinates with either Ar−Cl or Ar−Br substituents were tested and products **170m**–**o** were generated in modest to good yields (39–69%). Only traces of dehalogenated styrene were observed, which was highly surprising i) in contrast to the reports of Wickens and Lin/Lambert (vide supra), ii) given the potent reductive power of *[*n*-BuO-NpMI]^•−^ and iii) despite the similar redox potentials of phosphinates and aryl halides (*E*^p^_red_ = −2.4 V for 4-bromobiphenyl [308]). The authors’ computational, spectroscopic and catalyst structural variation experiments (i.e., **NpMI** did not work) revealed that the *N*-aniline moiety of the radical anion catalyst engages in an intimate and selective preassembly with target substrates, facilitating a rate determining C(sp^3^)–O cleavage step and ensuring redox chemoselectivity over dehalogenations/other reductive cleavages. The preference for a π-stacking preassembly of the *N*-aniline moiety of ***n*-BuO-NpMI^•−^** at the diarylphosphinate group, rather than the aryl halide, could rationalize the tolerance of aryl halides. Finally, styrene-forming substrates containing a longer-chain aliphatic or a benzyl group retained high *E*-isomer selectivity (**170p**,**q**). e-PRC deoxygenations were also achieved, affording 1,2-diphenylethane **171a** and limonene **171b**.

Analogously to Mikaye’s conPET study (vide supra, section 2.1.4), the authors observed a nanosecond-lived emitting state with an *E*_0-0_ value (56.6 kcal mol^−1^) similar to the triplet energy of the *E*-stilbene (51 kcal mol^−1^), which rationalized the Z-stilbene formation by an energy transfer *E/Z*-photoisomerization pathway. This was ascribed to a quartet state *^4^[***n*-BuO-NpMI^•−^**], arising from ISC from the doublet state *^2^[***n*-BuO-NpMI^•−^**]. The possibility of a closed-shell decomposition product of the catalyst with a similar singlet excited state lifetime (as reported by Nocera and co-workers) functioning in the *E*/*Z*-stilbene photoisomerism cannot be fully ruled out [324]. However, the emitting species/a closed-shell decomposition species can be excluded as the SET reductant since i) its TCSPC lifetime was not quenched by phosphinate esters, ii) the clear influence of the *N*-aniline substituent, electronically disconnected from the naphthalene moiety which is where the transformation to the closed-shell decomposed catalyst occurs.

**Figure 74:**
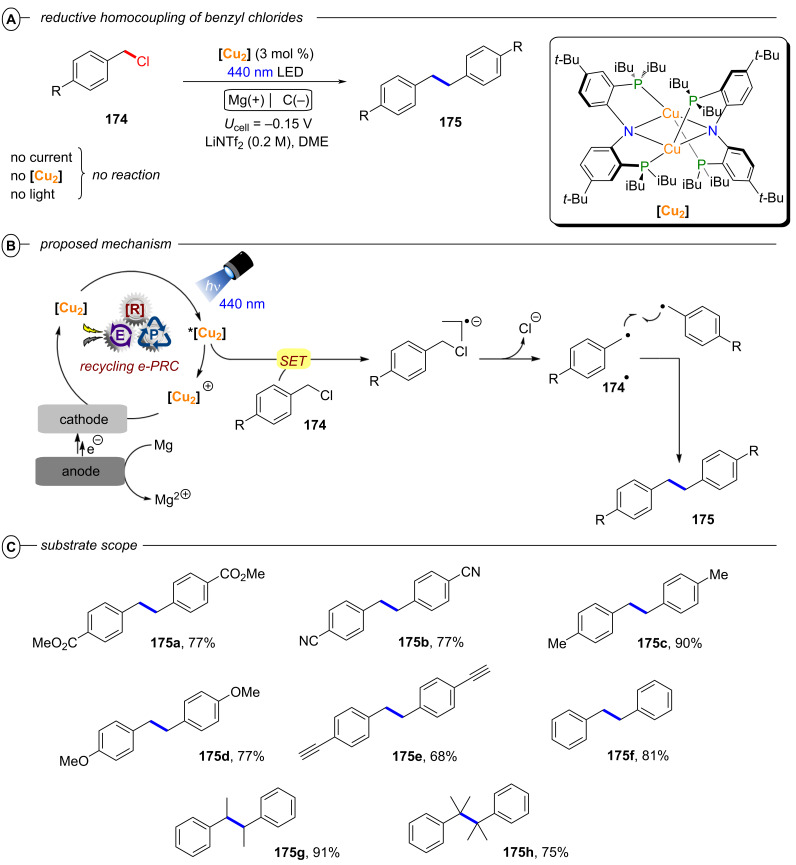
A) Recycling e-PRC reductive dimerization of benzylic chlorides using a [Cu_2_] catalyst. B) Proposed mechanism. C) Substrate scope.

Shifting focus away from C(sp^3^)–O cleavages to C(sp^3^)–Cl cleavages, the direct generation of R^•^ from alkyl halides – that are challenging to reduce [325–326] – is a crucial aspect in modern chemical transformations [327–332] that allows i) to avoid issues with traditional alkylation and ii) to open a complementary radical reactive mode. Hence, there has been a considerable interest in expanding the pool of alkyl halides compatible with this type of transformation [325,333–334]. Peters and co-worker reported how a dicopper diamond-type complex (referred to as **[Cu_2_]** in Figure 74A), previously developed in their laboratory [335], served as an interesting electro-recycled photoredox catalyst for the reductive dimerization of benzylic chlorides **174** (Figure 74A) [336]. According to the authors, this specific catalyst was ideal for reductive processes owing to i) its charge delocalization over the Cu_2_(*μ*-N)_2_ core, ii) the steric shieldng of the isobutyl and *tert*-butyl groups which renders the one-electron oxidized state **[Cu_2_]^+^** non-nucleophilic, avoiding undesirable reactions of the benzylic radical and the ligands.

The authors proposed a mechanism based on Stern–Volmer (SV) and time-resolved luminescence spectroscopy experiments. Firstly, **[Cu_2_]** is photoactivated to generate a potent excited-state reductant, *[Cu_2_] (*E_1/2_ = −2.7 V vs SCE). SET reduction of benzyl chloride **174** (R = Me, *E*^p^_red_ = −2.5 V vs SCE) yields benzylic radical **174^•^** and the ground state oxidized catalyst **[Cu_2_]^+^** (Figure 74B). Dimerization of **174^•^** generates coupling product **170**, while **[Cu_2_]^+^** is reduced at the cathode to complete a recycling e-PRC process. Under the optimized reaction conditions, LiNTf_2_ serves two roles: i) a supporting electrolyte, and ii) a chloride scavenger; since the chloride byproduct from reductive C–Cl bond cleavage can interfere with **[Cu_2_]^+^**. This undesirable reaction between catalyst and chloride was confirmed by observing the color change of a solution containing **[Cu]^+^** with or without LiCl. Without the addition of the chloride source, the solution appeared brown in DME, a color attributed to the active form of the catalyst. In the presence of LiCl, a loss of red-brown color occurred over several hours, resulting in a yellow solution and inactive catalyst. The optimal reaction conditions tolerated electron-withdrawing groups *para*- to the benzylic chloride such as esters and nitriles (**170a**,**b**, Figure 74C) as well as electron-donating groups like methyl, methoxy (**170c**,**d**) and alkynyl (**170e**) groups. Secondary and tertiary benzylic chlorides were successfully reductively dimerized, affording the products **170f**–**h** in good yields. However, the transformation was limited to homodimerizations and no crossed dimerizations were achieved.

**Figure 75:**
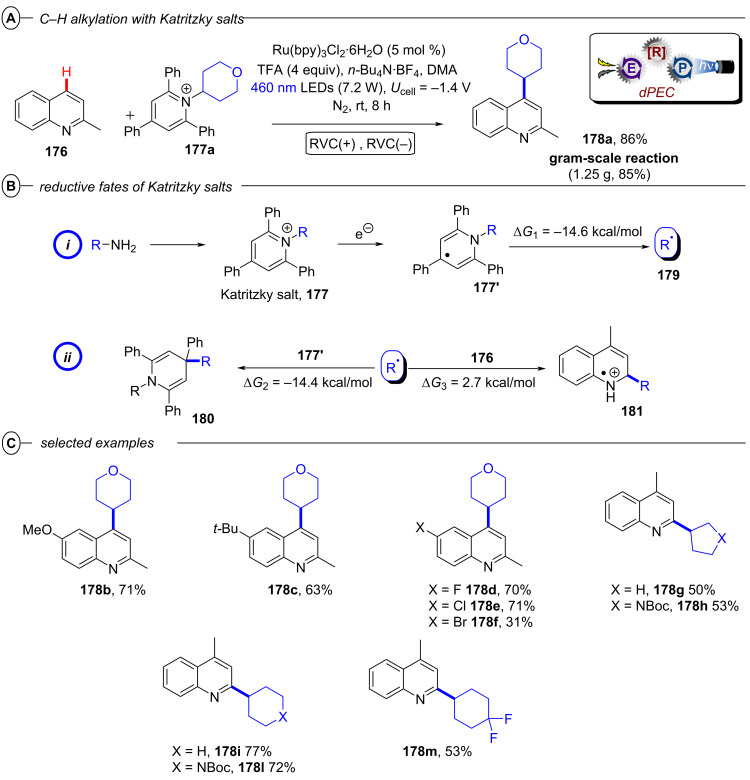
A) Decoupled photoelectrochemical C*–*H alkylation of heteroarenes through deamination of Katritzky salts. B) Alkyl radical generation and competing pathways for the downstream radical chemistry. C) Selected examples from the substrate scope.

Finally, a C(sp^3^)−N bond cleavage was achieved by the group of Chen and co-workers, complementing the well-explored field of PEC alkylation of heteroarenes (vide supra, section 3.1.2). The authors employed *d*PEC deamination (Figure 75A) in a reductive process [337] that cleaved Katritzky salts **177** [338–340] to generate alkyl radicals. Broadly speaking, SET reduction of **177** produces species **177’**, followed by exothermic fragmentation that provides alkyl species **179** (Figure 75B-*i*). However, compared to endothermic addition of radicals to heterocycles, exothermic radical coupling with precursor **177’** is often observed thus inhibiting the target reaction (Figure 75B-*ii*) [341–342]. Despite the less than encouraging premises [343], Chen and co-workers managed to develop a deaminative *d*PEC C–H alkylation method for quinoline **176** using Ru(bpy)_3_∙6H_2_O as a photocatalyst, obtaining the products in good yields with minimum quantities of byproduct **180** (Figure 75B). According to the authors, constant potential conditions were indispensable to selectively reduce the Katritzky salts and not the quinolines and/or the products.

**Figure 76:**
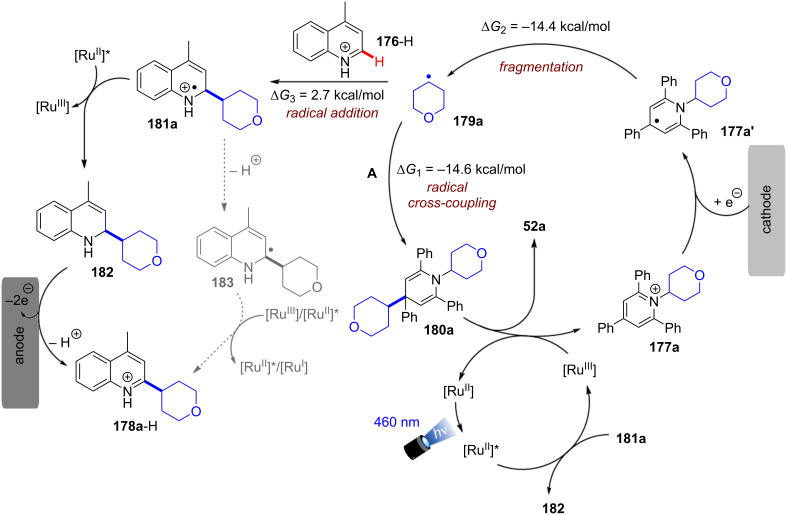
Proposed mechanism by Chen and co-workers.

As shown in Figure 75C, when **176** was substituted at the 2-position, alkylation selectively occurred at the 4-position (such as in **178a**), while 2-position was regioselectivity obtained starting from 4-position substituted starting materials (such as **178g**). Regarding the quinoline derivatives scope, electron-donating groups such as methoxy or *tert*-butyl worked well with different acceptors (**178b**,**c** in Figure 75C). Weakly electron-withdrawing functionalities, for example, fluoro- and chloro-substituted substrates, worked smoothly (**178d** and **178e**) and bromo-substituted substrates provided the corresponding product in moderate yields accompanied by a debromination byproduct (**178f**). Then, several alkyl partners were tested, resulting in products such as **178g**–**m** using cyclopentyl- and cyclohexyl- derivatives. Finally, the authors successfully scaled the process in a batch set-up for the model substrate, obtaining **178a** on a 6.5 mmol scale with 85% yield. Through detailed investigations (CV analysis, spectrometric analysis, UV–vis and quenching studies) the authors proposed a *d*PEC mechanism (Figure 76).

Firstly, cathodic reduction of the Katritzky salt **177a** produces the persistent radical species **177a’**, followed by its exothermic fragmentation to generate alkyl radical **179a**, whose BHT complex was observed in GC–MS analysis by the authors. The latter incurred endothermic radical addition to the protonated quinoline **176**-H, leading to the radical **181a**, whose SET reduction by excited state *[Ru^II^(bpy)_3_] delivers the intermediate **182** (detected by HRMS) and regenerates **[Ru^III^(bpy)_3_]**. The protonated product **178a**-H is obtained through anodic oxidation of **182** (black path). Alternatively, the authors proposed deprotonation of **181a** would afford radical **183**, which could be oxidized by *[Ru^II^(bpy)_3_] or **[Ru^III^(bpy)_3_]** (grey-dashed path). Lastly, although during the electrolysis considerable quantities of radical exergonic cross-coupling product **180a** are obtained, which can be oxidized by the **[Ru^III^]** species to regenerate **177a**.

## Summary and Outlook

The Review reported consecutive photoinduced electron transfer and synthetic photoelectrochemistry (with a focus on electro-activated photoredox catalysis) as two promising fields that offer unique advantages and challenges to overcome the energetic limitations of photoredox catalysis. While both approaches involve SET reactions of the excited state of a photocatalyst and organic substrates, they differ in the generation of the active catalyst species and the reaction mechanism. In particular, we propose the most significant difference between the two techniques is *the steady-state concentration of in situ-generated radical ion catalyst* (generally lower for conPET due to the less efficient PET generation and the propensity for back-electron transfer). This heavily influences the chemoselectivity and ability of the catalyst to preassemble with target substrates.

Also, the profile of catalyst voltammograms might have interesting implications on the choice of conPET/e-PRC and the resulting reactivity. Catalysts with fully reversible voltammograms are generally required for e-PRC, permitting high steady-state concentrations of the active photocatalyst. On the other hand, catalysts exhibiting irreversible (or quasi-reversible) voltammograms might be employable in conPET due to the regulation of a lower steady-state concentration of the active photocatalyst by competing PET generation/BET reversion processes. The peculiarities of each method make them perfectly complementary: conPET in the oxidative direction is less explored than in the reductive direction, probably due to the lack of colored precursors to oxidative photocatalysts. On the other hand, e-PRC boasts a lot of examples for oxidative activations of organic functionalities – likely owing to the hydrogen evolution reaction (HER) as the ideal reductive counter reaction – yet less so for the reductive direction (possibly due to complications derived from sacrificial anode materials or the competing oxygen evolution reaction).

Regarding the initial ‘inhibition barrier’ for experimental application in academia and industry, conPET appears more easily accessible and scalable. However, considering that i) photons cost more than electrons and ii) the cost of sacrificial chemical additives, PEC is ultimately the superior technology for industrial applications once standardized (flow) reactors can be developed to i) bring light and current/potential into the same flow path, ii) eliminate supporting electrolyte and iii) render robust, reproducible processes. Although the lack of tailor-made reactors has hampered the advancement of ‘single pass flow’ PEC protocols, several examples of gram-scale reactions in batch or recirculated flow are already present.

In conclusion, further developments of both techniques are desirable. These include for conPET i) the expansion of oxidative activations pool, ii) the exploration of neutral radical photocatalysts, and iii) the development of ‘redox neutral’ conPET reactions that consolidate both redox-activated partners into a single product (surprisingly, this is yet to be achieved). Regarding e-PRC, cutting-edge technologies to establish truly scalable processes are required. Of high priority in both e-PRC and conPET reactions is conducting further investigations into the mechanism. In particular, noncovalent aggregations of photocatalysts and substrates is a topic receiving increasing attention in the literature [344–345], and – specifically for radical ion photocatalysis – further investigations into catalyst-substrate pre-association are urgently needed to i) explain unexpected wavelength dependencies and ii) to rationalize how ultrashort-lived excited states can provide productive photochemistry. Transformation of the species designated ‘catalyst’ (to another photoactive catalyst) and how this is influenced by reaction conditions/concentration (conPET/e-PRC) is also a priority topic for investigation. Such investigations will provide better understanding of these techniques and could potentially lead to the discovery of more efficient and selective catalysts.
